# Development of locally relevant clinical guidelines for procedure-related neonatal analgesic practice in Kenya: a systematic review and meta-analysis

**DOI:** 10.1016/S2352-4642(20)30182-6

**Published:** 2020-10

**Authors:** Cian Wade, John Scott Frazer, Evelyn Qian, Lien M Davidson, Suzanne Dash, Anna te Water Naudé, Rema Ramakrishan, Jalemba Aluvaala, Kokila Lakhoo, Mike English

**Affiliations:** a Medical Sciences Division, University of Oxford, Oxford, UK; b Nuffield Department of Women's and Reproductive Health, University of Oxford, Oxford, UK; c Nuffield Department of Surgical Sciences, University of Oxford, Oxford, UK; d Centre for Tropical Medicine and Global Health, Nuffield Department of Medicine, University of Oxford, Oxford, UK; e Oxford University Hospitals NHS Foundation Trust, Oxford, UK; f University of New South Wales, Faculty of Medicine, Sydney, NSW, Australia; g KEMRI Wellcome Trust, Nairobi, Kenya; h Department of Paediatrics, University of Nairobi, Nairobi, Kenya

## Abstract

Background

Increasing numbers of neonates are undergoing painful procedures in low-income and middle-income countries, with adequate analgesia seldom used. In collaboration with a multi-disciplinary team in Kenya, we aimed to establish the first evidence-based guidelines for the management of routine procedure-related neonatal pain that consider low-resource hospital settings.

**Methods:**

We did a systematic review by searching MEDLINE, Embase, CINAHL, and CENTRAL databases for studies published from Jan 1, 1953, to March 31, 2019. We included data from randomised controlled trials using heart rate, oxygen saturation (SpO_2_), premature infant pain profile (PIPP) score, neonatal infant pain scale (NIPS) score, neonatal facial coding system score, and douleur aiguë du nouveau-né scale score as pain outcome measures. We excluded studies in which neonates were undergoing circumcision or were intubated, studies from which data were unextractable, or when pain was scored by non-trained individuals. We did a narrative synthesis of all studies, and meta-analysis when data were available from multiple studies comparing the same analgesics and controls and using the same outcome measures. 17 Kenyan health-care professionals formed our clinical guideline development panel, and we used the Grading of Recommendations, Assessment, Development and Evaluation framework and the panel's knowledge of the local health-care context to guide the guideline development process. This study is registered with PROSPERO, CRD42019126620.

**Findings:**

Of 2782 studies assessed for eligibility, data from 149 (5%) were analysed, with 80 (3%) of these further contributing to our meta-analysis. We found a high level of certainty for the superiority of breastfeeding over placebo or no intervention (standardised mean differences [SMDs] were −1·40 [95% CI −1·96 to −0·84] in PIPP score and −2·20 [–2·91 to −1·48] in NIPS score), and the superiority of oral sugar solutions over placebo or no intervention (SMDs were −0·38 [–0·61 to −0·16] in heart rate and 0·23 [0·04 to 0·42] in SpO_2_). We found a moderate level of certainty for the superiority for expressed breastmilk over placebo or no intervention (SMDs were −0·46 [95% CI −0·87 to −0·05] in heart rate and 0·48 [0·20 to 0·75] in SpO_2_). Therefore, the panel recommended that breastfeeding should be given as first-line analgesic treatment, initiated at least 2 min pre-procedure. Given contextual factors, for neonates who are unable to breastfeed, 1–2 mL of expressed breastmilk should be given as first-line analgesic, or 1–2 mL of oral sugar (≥10% concentration) as second-line analgesic. The panel also recommended parental presence during procedures with adjunctive provision of skin-to-skin care, or non-nutritive sucking when possible.

**Interpretation:**

We have generated Kenya's first neonatal analgesic guidelines for routine procedures, which have been adopted by the Kenyan Ministry of Health, and have shown a framework for clinical guideline development that is applicable to other low-income and middle-income health-care settings.

**Funding:**

Wellcome Trust Research Programme, and the Africa-Oxford Initiative.

## Introduction

Global efforts to reduce neonatal mortality have led to substantial increases in the numbers of neonates being treated as inpatients. Observational studies have shown that neonates often undergo more than a dozen painful procedures per day while on neonatal intensive care units.^1^ Furthermore, untreated pain is associated with significant neurophysiological and developmental consequences.^2^ Ethics boards, the neonatology community, and parents have emphasised the need to minimise pain,^3^ but the paucity of clear guidelines, busy clinical environments, and difficulty in reliably measuring pain in neonates have resulted in an ongoing and substantial burden of unaddressed neonatal pain.^4^

In resource-poor settings, periprocedural analgesia is rarely administered. A national cross-sectional survey done in Kenya found that over a single day, no neonate received analgesia for any of the 404 routine procedures that were done.^5^ Untreated neonatal pain, therefore, represents a huge global source of short-term and potentially long-term clinical morbidity. In view of increasing attention paid to patients' health-care experiences, especially in vulnerable groups and their families, inadequate treatment of pain is also an important quality-of-care concern.^6^

**Research in context**

**Evidence before this study**

Generic global guidance for low-income and middle-income countries (LMICs) on neonatal (aged ≤28 days) pain management is lacking. Local practice or guidance varies substantially and might not be based on the best available evidence, and recommendations for high-income countries are not suitable for all health-care settings. In Kenya specifically, no evidence-based neonatal analgesic guidelines for non-tertiary hospitals exist, which probably contributes substantially to the observed paucity of provision of any form of analgesia during routine, painful procedures. We searched MEDLINE, Embase, CINAHL, and the Cochrane Central Register of Controlled Trials for studies or guidelines published between Jan 1, 1953, and March 31, 2019, with search terms relevant to a range of analgesics and procedures commonly seen in the non-tertiary LMIC setting. This search did not identify any analgesic guidelines specifically for neonates undergoing routine procedures in the LMIC setting.

**Added value of this study**

This study describes specific recommendations generated by a local panel of neonatal experts for the management of routine procedure-related pain, which have subsequently been adopted nationally in Kenya. These recommendations were generated after discussion of the findings of a rigorous systematic review and meta-analysis, application of the Grading of Recommendations, Assessment, Development and Evaluation framework to this evidence, and local context-specific considerations. We have shown a framework for clinical guideline development, which can be applied to other clinical contexts in LMICs.

**Implications of all the available evidence**

Based on our findings, when possible, breastfeeding should be promoted above all other analgesic strategies during routine procedures in district and county hospitals. For neonates who are unable to breastfeed, expressed breastmilk is preferred, but oral sugar solutions are an adequate alternative. Periprocedural parental presence should be encouraged, and adjunctive provision of skin-to-skin care or non-nutritive sucking should be provided to all neonates when appropriate. Clinical care in LMICs can be improved by efficient use of high-quality systematic reviews linked to structured decision making processes to generate recommendations, which place local context at the heart of the process.

Previous work has mostly been studies of single, discrete interventions,^7^, ^8^, ^9^ and many existing clinical guidelines do not account sufficiently for variation in local resources and patient demographics.^10^, ^11^ Clinicians, therefore, face difficulties in making good, evidence-based choices for particular analgesic strategies in specific patient populations.

We did a systematic review and meta-analysis of the evidence relating to a range of analgesics and procedures appropriate to populations at the first-referral hospital level prioritised by WHO.^12^ This non-tertiary health-care setting is represented by the county hospitals in Kenya, and by the district hospitals in other low-income and middle-income countries. We aimed to translate the existing evidence and generate new national guidelines for managing neonatal pain during routine procedures in Kenya.

## Methods

### Search strategy and selection criteria

In this systematic review and meta-analysis, a group consisting of experts in global neonatal and paediatric care determined the inclusion and exclusion criteria, and the various population, intervention, control, outcome (PICO) questions under consideration. Procedures and analgesics included those that are most widely seen in our target setting of non-tertiary Kenyan hospitals (panel 1).

Panel 1 **Procedures, analgesics, and outcome measures included in the systematic review**

**Procedures**

• Heel prick
• Intramuscular or subcutaneous injections
• Venepuncture or venous cannulation
• Arterial puncture
• Continuous positive airway pressure prongs insertion
• Lumbar puncture
• Urinary catheterisation

**Analgesics**

• Breastfeeding
• Expressed breastmilk
• Oral sugar solutions (sucrose, glucose, dextrose, sweetener, or fructose)
• Skin-to-skin care (including kangaroo mother care)
• Non-nutritive sucking
• Swaddling
• Music
• Topical local anaesthetic
• Paracetamol
• Ibuprofen
• Morphine
• Ketamine

**Outcome measures**

• Heart rate
• Transcutaneous oxygen saturation
• Premature infant pain profile score
• Neonatal infant pain scale score
• Neonatal facial coding system score
• Douleur aiguë du nouveau-né scale score

We searched MEDLINE, Embase, CINAHL, and the Cochrane Central Register of Controlled Trials databases for articles published between Jan 1, 1953, and March 31, 2019 (appendix p 1), without any language restrictions. We identified additional studies by screening references found in systematic reviews identified in our original search. Following the literature search, we retrospectively excluded studies on neonates undergoing circumcision because circumcision was felt to represent a severe pain stimulus over a prolonged time period, and risked confounding results when combined with procedures inducing more acute pain.

Two authors (CW and JSF) independently screened titles, abstracts, and full texts for eligibility. Any discrepancies were resolved through consensus discussion with an additional third author. We included only randomised and quasi-randomised controlled trials done on neonates (mean postnatal age 0–28 days). Our study focused on the physiological and behavioural measures of neonatal pain most commonly used in the experimental setting. These measures were heart rate, oxygen saturation (SpO_2_), premature infant pain profile (PIPP) score,^13^ neonatal infant pain scale (NIPS) score,^14^ neonatal facial coding system (NFCS),^15^ and douleur aiguë du nouveau-né (DAN) scale.^16^ In the absence of clear evidence of the superiority of any of these measures over the others, all six were deemed critical outcomes from a Grading of Recommendations, Assessment, Development and Evaluation (GRADE)^17^ perspective. These six measurements could not be combined into a single outcome measure of pain and were therefore analysed separately. The exclusion of other potential measures of neonatal pain, such as respiratory rate and crying duration, was based upon inferior evidence of the validity of these measures, and their less frequent use precluding meaningful meta-analysis.^6^, ^14^ Additional exclusion criteria were studies in which neonates were intubated, studies from which data were unextractable, or when pain was scored by non-trained individuals. We combined no intervention and placebo groups for the purposes of our analysis, having considered that the well described placebo analgesic effect seen in adults is significantly less pronounced in neonates, and on the basis of sensitivity analyses of our own data (appendix pp 50–55).^18^, ^19^

Data were extracted in duplicate from eligible studies by two authors (CW and JSF) using Covidence systematic review software. When data were presented only graphically, the two authors discussed and generated a consensus estimate, excluding studies when representation was ambiguous. Two authors (EQ and AtWN) independently assessed the quality of the included studies and the potential risk of bias using the Cochrane risk of bias tool.^20^ Any discrepancies between authors in data extraction or quality assessment were resolved through consensus discussion with an additional third author. The protocol is registered with PROSPERO, CRD42019126620.

### Data analysis

Pre-piloted documents developed by the authors were used to summarise the data from every included study within a given PICO question. The data encapsulated within these documents enabled the review group to compose a narrative synthesis of the qualitative information captured by all studies matching eligibility criteria in our review, and to supplement meta-analysis findings.

Meta-analysis could only be done when data were available from multiple studies comparing the same analgesics and controls and using the same outcome measures (appendix p 2). For each study, mean, SD, and sample size were extracted or calculated for each comparison group's pain outcome measure. We used the DerSimonian and Laird random-effects model^21^ to compute standardised mean difference (SMD; Hedges' *g*) and pool estimates for each exposure comparison and outcome. We assessed heterogeneity between the studies included in the analysis using the *I*^2^ statistic. For studies included in the meta-analysis, we used funnel plot and Egger's regression asymmetry test to assess publication bias and small-study effects for comparisons with ten or more studies for each outcome.

### Clinical guideline development

17 Kenyan health-care professionals formed the Neonatal Pain Guideline Group (NPGG), which constituted our clinical guideline development panel. This group included four neonatologists, seven paediatricians, two pharmacists, two neonatal nurses, one lecturer in neonatology, and one representative from the Kenyan Clinical Officers Council. NPGG members were selected for their multi-disciplinary representation of important stakeholder groups, on the basis of the members' expertise in non-tertiary Kenyan neonatal care and engagement in previous clinical guideline development processes. NPGG members declared no competing interests. We were unable to include representatives of patients' families or do formal cost-effectiveness analyses.

Based on the findings of the systematic review, the key clinical question that the NPGG sought to answer was: in neonates, which analgesics should be recommended to reduce pain while undergoing routine procedures? This broad question was handled by considering individual PICO questions within each analgesic category using the GRADE framework. This is a transparent framework for guideline developers to make specific clinical recommendations on the basis of certainty in the available evidence. Elements of the GRADE approach include risk of bias of studies, inconsistency of findings, indirectness of the evidence, imprecision of the effect estimate, and publication bias. Several NPGG members were familiar with the GRADE process, and the NPGG meeting began with a presentation on use of the framework. Following this, detailed findings of the meta-analysis and narrative synthesis were presented by CW and discussed by the NPGG. This process was repeated, with the NPGG reaching consensus on whether certainty in the evidence of the observed effect was high, moderate, low, or very low for each specific PICO question to produce the final GRADE summary of findings tables.

With these tables as the starting point, the NPGG discussed how these should inform recommendations for context-appropriate clinical guidelines. Discussions considered the balance of benefits and harms of each intervention, local feasibility, compatibility with broader policy agendas, and likely staff and family preferences. At the end of the 1-day NPGG meeting, consensus recommendations were agreed. These recommendations were circulated to all NPGG members in written form for further clarification and review over a period of 3 weeks. All NPGG members approved the final recommendations.

### Role of the funding source

The funder of the study had no role in study design, data collection, data analysis, data interpretation, or writing of the report. The corresponding author had full access to all the data in the study and had final responsibility for the decision to submit for publication.

## Results

**Figure 1:**
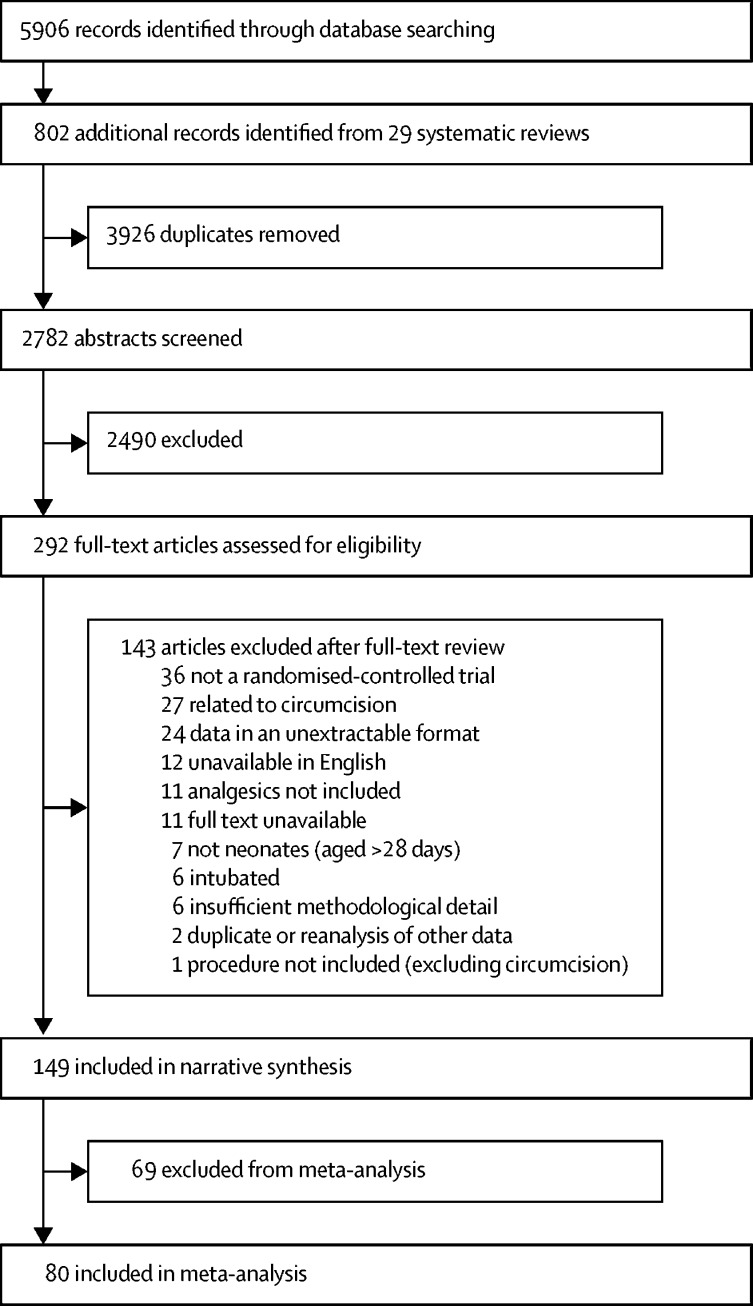
Study selection

We retrieved 5906 records, with 802 additionally found through screening systematic reviews identified in our original search. After removal of duplicates, 2782 (41%) abstracts were screened, with 292 (10%) deemed eligible for full-text screening. Of these, 149 (5%) studies were included in the narrative synthesis (n=13 169), and 80 (54%) of these 149 in the meta-analysis (n=5869; figure 1; appendix pp 3–6).

**Figure 2:**
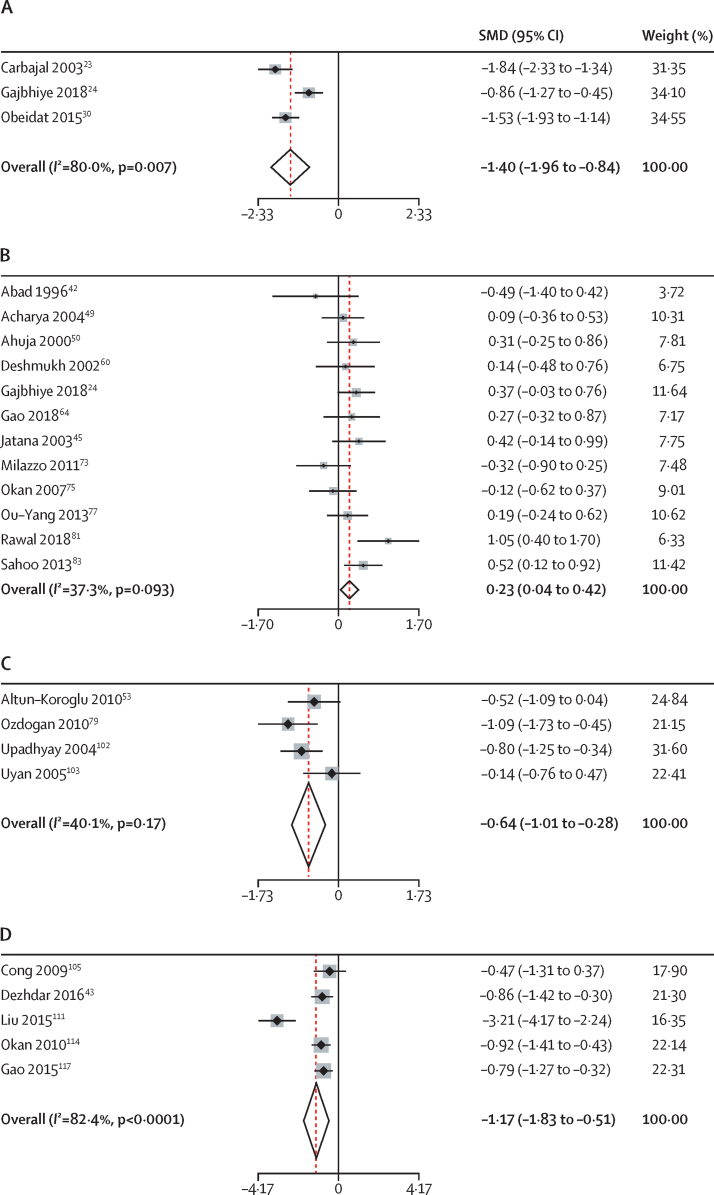
Selected random-effects meta-analyses of main findings (A) Breastfeeding *vs* placebo or no intervention for premature infant pain profile score. (B) Oral sugar *vs* placebo or no intervention for oxygen saturation. (C) Expressed breastmilk *vs* placebo or no intervention for neonatal facial coding system score. (D) Skin-to-skin care *vs* placebo or no intervention for heart rate. SMD=standardised mean difference.

PICO question comparisons that were prioritised by the NPGG in their discussions were those involving breastfeeding, oral sugar, expressed breastmilk, skin-to-skin care, and non-nutritive sucking.^22^, ^23^, ^24^, ^25^, ^26^, ^27^, ^28^, ^29^, ^30^, ^31^, ^32^, ^33^, ^34^, ^35^, ^36^, ^37^, ^38^, ^39^, ^40^, ^41^, ^42^, ^43^, ^44^, ^45^, ^46^, ^47^, ^48^, ^49^, ^50^, ^51^, ^52^, ^53^, ^54^, ^55^, ^56^, ^57^, ^58^, ^59^, ^60^, ^61^, ^62^, ^63^, ^64^, ^65^, ^66^, ^67^, ^68^, ^69^, ^70^, ^71^, ^72^, ^73^, ^74^, ^75^, ^76^, ^77^, ^78^, ^79^, ^80^, ^81^, ^82^, ^83^, ^84^, ^85^, ^86^, ^87^, ^88^, ^89^, ^90^, ^91^, ^92^, ^93^, ^94^, ^95^, ^96^, ^97^, ^98^, ^99^, ^100^, ^101^, ^102^, ^103^, ^104^, ^105^, ^106^, ^107^, ^108^, ^109^, ^110^, ^111^, ^112^, ^113^, ^114^, ^115^, ^116^, ^117^, ^118^, ^119^, ^120^, ^121^, ^122^ Complete results from meta-analysis relevant to these PICO questions involving breastfeeding, oral sugar, expressed breastmilk, skin-to-skin care, and non-nutritive sucking^22^, ^23^, ^24^, ^25^, ^26^, ^27^, ^28^, ^29^, ^30^, ^31^, ^32^, ^33^, ^34^, ^35^, ^36^, ^37^, ^38^, ^39^, ^40^, ^41^, ^42^, ^43^, ^44^, ^45^, ^46^, ^47^, ^48^, ^49^, ^50^, ^51^, ^52^, ^53^, ^54^, ^55^, ^56^, ^57^, ^58^, ^59^, ^60^, ^61^, ^62^, ^63^, ^64^, ^65^, ^66^, ^67^, ^68^, ^69^, ^70^, ^71^, ^72^, ^73^, ^74^, ^75^, ^76^, ^77^, ^78^, ^79^, ^80^, ^81^, ^82^, ^83^, ^84^, ^85^, ^86^, ^87^, ^88^, ^89^, ^90^, ^91^, ^92^, ^93^, ^94^, ^95^, ^96^, ^97^, ^98^, ^99^, ^100^, ^101^, ^102^, ^103^, ^104^, ^105^, ^106^, ^107^, ^108^, ^109^, ^110^, ^111^, ^112^, ^113^, ^114^, ^115^, ^116^, ^117^, ^118^, ^119^, ^120^, ^121^, ^122^ are presented in figure 2 and in the appendix (pp 12–64).

The NPGG's GRADE certainty in the evidence for these prioritised comparisons is shown in the appendix (p 7). The appendix (p 8) presents a summary of the narrative synthesis described here and the associated references for each PICO question. GRADE summaries for other PICO questions considered by the NPGG but not discussed here due to the NPGG deprioritising them on the basis of local feasibility and analgesic efficacy are shown in the appendix (pp 9, 68–69). Within the 149 studies, heel prick was the most commonly observed procedure (88 [59%] studies), oral sugar the most commonly used analgesic (78 [52%] studies), and heart rate the most commonly used pain outcome measure (76 [51%] studies). Full tallies are presented in the appendix (p 10).

12 studies (n=991) compared breastfeeding with placebo or no intervention.^22^, ^23^, ^24^, ^25^, ^26^, ^27^, ^28^, ^29^, ^30^, ^31^, ^32^, ^33^ Meta-analysis showed evidence for a reduction in PIPP score (SMD −1·40 [95% CI −1·96 to −0·84]; n=327) and NIPS score (−2·20 [–2·91 to −1·48]; n=225; figure 2A; appendix p 14), although not for heart rate (−1·49 [–3·44 to 0·46]; n=210) or SpO2 (0·59 [–1·26 to 2·43]; n=150; appendix pp 12, 13). Narrative synthesis further supported this finding with all 12 studies finding a superiority of breastfeeding compared with placebo or no intervention (appendix p 8). Therefore, the NPGG had a high level of certainty in the superiority of breastfeeding over placebo or no intervention when initiated at least 2 min before the painful procedure (appendix p 7). However, the NPGG had a low certainty in the superiority of breastfeeding over oral sugar (appendix p 7). Narrative synthesis (n=670) revealed inconsistencies, with five (63%) of eight studies with direct comparisons suggesting breastfeeding was superior to oral sugar,^24^, ^34^, ^35^, ^36^, ^37^ two (25%) showing equivalence,^38^, ^39^ and one (13%) inferiority to oral sugar (appendix p 8).^40^ Meta-analysis suggested no difference using PIPP score (−0·21 [–0·78 to 0·35]; n=346), but superiority of breastfeeding using NIPS score (−1·51 [–2·48 to −0·53]; n=182; appendix pp 15, 16). Based upon results from three studies (n=136), the NPGG had a moderate certainty in the superiority of breastfeeding over giving expressed breastmilk (appendix p 7), with two studies showing superiority with a large effect size,^27^, ^34^ and one showing inferiority (appendix p 8).^39^ Finally, the NPGG found a moderate certainty in the superiority of breastfeeding over skin-to-skin care (appendix p 7), with both studies (n=160) showing breastfeeding's superiority for NIPS score (−1·52 [–2·82 to −0·22]; n=160; appendix p 17).^37^, ^41^

Oral sugar solutions ranged in volume from 0·05 mL to 5 mL, concentration from 5% to 50% (median 25%), and solution type included sucrose (52 studies), glucose (33 studies), dextrose (eight studies), sweetener (two studies), and fructose (one study). When considering the six studies (n=453) directly comparing the efficacy of various concentrations of oral sugar, the NPGG had a moderate certainty in the superiority of solutions with a concentration of 24% or more over solutions with a concentration below 24% (appendix p 7).^42^, ^43^, ^44^, ^45^, ^46^, ^47^ However, the NPGG found insufficient evidence to draw any conclusions regarding whether increasing the solution concentration above 24% showed an analgesic benefit (appendix p 7). Based on indirect comparisons across studies, the NPGG had a moderate certainty that increasing the volume of oral sugar solutions above 2 mL showed no benefit (appendix p 7). When sufficient numbers of studies for a given PICO question allowed, sensitivity analyses did not suggest any difference in the analgesic efficacy of various types of sugar solutions for measures of heart rate (p=0·28) or PIPP score (p=0·33) but suggested possible superiority of dextrose for SpO_2_ (p=0·03; appendix pp 41–49). On the basis of these findings, subsequent analysis of the overall efficacy of oral sugar considered all solutions together irrespective of solution type, concentration, or volume.

58 studies (n=3948) directly compared oral sugar solutions with placebo or no intervention.^24^, ^42^, ^44^, ^45^, ^46^, ^47^, ^48^, ^49^, ^50^, ^51^, ^52^, ^53^, ^54^, ^55^, ^56^, ^57^, ^58^, ^59^, ^60^, ^61^, ^62^, ^63^, ^64^, ^65^, ^66^, ^67^, ^68^, ^69^, ^70^, ^71^, ^72^, ^73^, ^74^, ^75^, ^76^, ^77^, ^78^, ^79^, ^80^, ^81^, ^82^, ^83^, ^84^, ^85^, ^86^, ^87^, ^88^, ^89^, ^90^, ^91^, ^92^, ^93^, ^94^, ^95^, ^96^, ^97^, ^98^, ^99^ 50 (86%) studies showed sugar to be superior in narrative synthesis,^24^, ^42^, ^44^, ^45^, ^46^, ^47^, ^48^, ^49^, ^50^, ^51^, ^52^, ^53^, ^54^, ^55^, ^56^, ^57^, ^58^, ^59^, ^60^, ^61^, ^62^, ^63^, ^64^, ^65^, ^66^, ^67^, ^68^, ^69^, ^70^, ^71^, ^72^, ^73^, ^74^, ^75^, ^76^, ^77^, ^78^, ^79^, ^80^, ^81^, ^82^, ^83^, ^84^, ^85^, ^86^, ^87^, ^88^, ^89^, ^90^, ^91^ and eight (14%) showed equivalence (appendix p 8).^92^, ^93^, ^94^, ^95^, ^96^, ^97^, ^98^, ^99^ This finding was supported by meta-analysis showing superiority of sugar for all outcome measures: heart rate (SMD=–0·38 [95% CI −0·61 to −0·16]; n=813); SpO_2_ (0·23 [0·04 to 0·42]; n=650); PIPP score (−1·00 [–1·58 to −0·41]; n=835); NIPS score (−1·01 [–1·69 to −0·32]; n=623); NFCS score (−0·77 [–1·36 to −0·17]; n=559), and DAN score (−0·96 [–1·42 to −0·50]; n=307; figure 2B; appendix pp 18–22). Therefore, the NPGG had a high certainty in this estimate of effect (appendix p 7). 14 studies (n=920) compared oral sugar directly with expressed breastmilk. We found some variation in the narrative synthesis, with nine (64%) studies suggesting the superiority of oral sugar,^45^, ^46^, ^76^, ^77^, ^79^, ^81^, ^83^, ^91^, ^100^ and five (36%) suggesting equivalence (appendix p 8).^34^, ^39^, ^53^, ^72^, ^99^ Meta-analysis did not show a significant difference between oral sugar and expressed breastmilk for heart rate (0·16 [–0·07 to 0·39]; n=293) SpO_2_, (−0·11 [–0·48 to 0·27]; n=283); PIPP score (0·55 [–0·03 to 1·12]; n=309), and NFCS score (0·93 [–0·29 to 2·15]; n=93; appendix pp 23–26). Therefore, the NPGG had a moderate certainty of the superiority of oral sugar over expressed breastmilk (appendix p 7). 11 studies (n=645) compared oral sugar with non-nutritive sucking, with two studies (18%) suggesting superiority of oral sugar,^91^, ^101^ six (55%) equivalence,^61^, ^64^, ^70^, ^88^, ^92^, ^98^ and three (27%) inferiority (appendix p 8).^58^, ^71^, ^72^ Meta-analysis revealed no difference between non-nutritive sucking and oral sugar for heart rate (−0·16 [–0·76 to 0·45]; n=151), SpO_2_ (−0·01 [–0·36 to 0·35]; n=122), NIPS score (−0·37 [–2·29 to 1·54]; n=188), or DAN score (−0·21 [–0·65 to 0·24]; n=77; appendix pp 27–30). Therefore, the NPGG had a low certainty in the superiority of oral sugar over non-nutritive sucking (appendix p 7).

14 studies (n=863) compared giving expressed breastmilk with placebo or no intervention, with nine (64%) showing superiority,^45^, ^53^, ^76^, ^77^, ^81^, ^83^, ^91^, ^99^, ^102^ and five (36%) equivalence (appendix p 8).^27^, ^46^, ^72^, ^79^, ^103^ We found evidence for a reduction in pain for heart rate (SMD=–0·46 [95% CI −0·87 to −0·05]; n=431), SpO_2_ (0·48 [0·20 to 0·75]; n=421); PIPP score (−1·47 [–2·48 to −0·46]; n=172), and NFCS score (−0·64 [–1·01 to −0·28]; n=217), but not DAN scores (−2·24 [–5·82 to 1·35]; n=132; figure 2C; appendix pp 31–34). Therefore, the NPGG had a moderate certainty in the superiority of expressed breastmilk over placebo or no intervention (appendix p 7).

The NPGG had a high certainty in the superiority of skin-to-skin care over placebo or no intervention (appendix p 7), with 15 (94%) studies suggesting the superiority of skin-to-skin care,^43^, ^94^, ^104^, ^105^, ^106^, ^107^, ^108^, ^109^, ^110^, ^111^, ^112^, ^113^, ^114^, ^115^, ^116^ and one (6%) suggesting equivalence (n=1054; appendix p 8).^116^ This finding was supported by comparisons of heart rate (SMD=–1·17 [95% CI −1·83 to −0·51]; n=264), although not SpO_2_ (0·41 [–0·06 to 0·88]; n=233) or NFCS score (−0·57 [–1·50 to 0·36]; n=392; figure 2D; appendix pp 35, 36).

Of the 16 studies (n=932) assessing non-nutritive sucking versus placebo or no intervention, 15 (94%) suggested the superiority of non-nutritive sucking,^29^, ^56^, ^58^, ^61^, ^64^, ^70^, ^71^, ^72^, ^88^, ^91^, ^98^, ^118^, ^119^, ^120^, ^121^ and one (6%) suggested equivalence (appendix p 8).^122^ Meta-analysis revealed evidence of pain reduction for PIPP score (SMD=–1·06 [95% CI −2·03 to −0·08]; n=137) and DAN score (−2·33 [–4·46 to −0·20]; n=124), but not for heart rate (−0·23 [–0·71 to 0·26]; n=306) or SpO_2_ (0·59 [–0·02 to 1·19]; n=104; appendix pp 37–40). Therefore, the NPGG found a moderate certainty in the superiority of non-nutritive sucking over placebo or no intervention (appendix p 7).

We found a high degree of heterogeneity (*I*^2^≥75%) for 22 (67%) of the 33 meta-analyses we were able to do for the analgesics outlined in panel 1 that the NPGG had prioritised (figure 2; appendix pp 12–40). Sensitivity analysis of oral sugar versus no intervention compared with versus placebo did not reveal a contribution to this observed heterogeneity. We found no significant interactions between oral sugar versus no intervention and oral sugar versus placebo for heart rate (p=0·36), SpO_2_, (p=0·72), and PIPP score (p=0·76; appendix pp 50–55). These findings also supported our having combined placebo and no intervention control groups for analysis. Further sensitivity analysis of oral sugar versus placebo or no intervention comparisons did not find that the timing of measuring pain outcomes during experiments contributed to heterogeneity. We found no significant interactions between pain scores measured 1 min or less and more than 1 min after the procedure commenced for heart rate (p=0·47), SpO_2_ (p=0·09), and PIPP (p=0·63; appendix pp 56–61). Prematurity (birth at <37 weeks' gestational age) might have contributed to heterogeneity in these comparisons with significant interaction between premature and full-term neonates for heart rate (p=0·002), but not SpO_2_ (p=0·65) or PIPP score (p=0·48; appendix pp 62–64). We also did sensitivity analyses according to the type of sugar solution used (appendix pp 41–49). Further investigations of sources of heterogeneity were limited by the requirement of a sufficient number of studies using the same comparisons and outcome measures to do these analyses.

Only comparisons of oral sugar versus placebo or no intervention could be assessed for publication bias because these groups were the only ones with ten or more studies for each outcome. We did not find evidence of publication bias or small-study effects in studies that reported differences in heart rate (p=0·13) or SpO_2_, (p=0·46; appendix pp 65, 66). However, we found possible publication bias or small-study effects for this comparison using PIPP scores (p=0·03; appendix p 67). We found that a substantial number of studies had a high overall risk of bias. 69 (46%) were deemed to have a low overall risk of bias, compared with 80 (54%) having a high overall risk of bias (appendix p 11).

After discussion of the findings of the narrative synthesis, meta-analysis, the final GRADE summary of findings tables, and the local health-care context to which these guidelines would be applied, the NPGG made the consensus recommendations detailed in panel 2. Despite low certainty of its superiority over giving oral sugar, breastfeeding was promoted as first-line analgesic because of an absent side-effect profile, and specific contextual factors such as free cost to the state, the promotion of maternal participation in care, and strong policy preference for exclusive breastfeeding in Kenya. Despite having a moderate certainty that expressed breastmilk was inferior to oral sugar, the group had concerns that use of oral sugar by health-care professionals might inadvertently be viewed as supporting the undesirable practice of offering glucose-water to newborn babies at home.^123^ Further concerns raised included the possible infection risk from reuse or shared use of bottles of sugar solution, and that neonates undergoing multiple procedures would receive large amounts of daily sugar. The NPGG were also concerned that recommending the potentially more effective sugar solutions with a concentration of 24% or more might result in health-care workers having to prepare this from the more readily available 10% and 50% solutions, and the potential harm caused from diluting with unsterile water. Therefore, for neonates who are unable to breastfeed, the NPGG recommended expressed breastmilk administered via syringe into the mouth as first-line analgesic, and oral sugar as second-line. In recommending the adjunctive provision of skin-to-skin care, or encouraging non-nutritive sucking in all possible cases, the NPGG considered the evidence of an enhanced analgesic effect from the combination of these analgesics compared with their use in isolation. The group was particularly keen to formally recommend these options because doing so would further encourage periprocedural parental involvement, something Kenyan health-care professionals have been aiming to promote.^124^

Panel 2 **Final recommendations for analgesia in neonates undergoing routine procedures in non-tertiary hospitals, made by the Neonatal Pain Guideline Group**

**If able to breastfeed**

• Breastfeeding should be the first-line analgesic, initiated at least 2 min before the procedure and, when possible, continued throughout
• When possible, the neonate should be held skin-to-skin with the mother
• Second-line and third-line analgesics should follow recommendations for neonates who are unable to breastfeed

**If unable to breastfeed**

• Expressed breastmilk is the first-line analgesic, with oral sugar a second-line option
• 1–2 mL of expressed breastmilk should be given via syringe into the mouth at least 2 min before the procedure, or 1–2 mL of any oral sugar solution (≥10% concentration) via syringe into the mouth at least 2 min before the procedure
• If dilution is required to reach the target concentration, then do so with sterile water
• When possible, position the neonate to prevent choking and stop administration if choking occurs
• Nasogastric or orogastric tubes should not be used for administration of these solutions for analgesic purposes; syringing of solutions into the mouth is still an option in most of these cases
• Caution should be taken not to administer too much sugar over a 24-h period when neonates are undergoing multiple procedures
• Skin-to-skin care or non-nutritive sucking of a neonate's own fist or a parent's cleaned finger after administration of expressed breastmilk or oral sugar should be promoted when appropriate

**General considerations specified by the Neonatal Pain Guideline Group**

• Emphasis should be placed on predicting the need for a procedure ahead of time and encouraging periprocedural parental presence
• Health-care professionals should make efforts to routinely measure neonatal pain around procedures by use of a validated pain measure score
• Pacifiers or dummies are generally not recommended for use for non-nutritive sucking

An updated literature search of the MEDLINE database (from April 1, 2019, to May 15, 2020) yielded a further 11 studies that would have met inclusion criteria. Having reviewed these studies, we do not feel that they reveal any unexpected new findings in the field beyond those already described in our original review. These studies support the superiority of breastfeeding, oral sugar, and non-nutritive sucking over other interventions, and further suggest that a combination of these interventions enhances analgesic efficacy in some instances. Therefore, we feel that the inclusion of these further studies was unlikely to have significantly altered the guideline decisions made by the NPGG.

## Discussion

We found that breastfeeding was the preferred analgesic option for neonates undergoing routine procedures in non-tertiary health-care settings in Kenya. Second-line options include expressed breastmilk and oral sugar, and the adjunctive provision of skin-to-skin care and non-nutritive sucking is also recommended. Our study focused upon the routine procedures and analgesics that are most widely seen in the non-tertiary Kenyan setting, and therefore we are cautious about the applicability of our recommendations to the higher-income setting. Moreover, we had important methodological concerns for many of our included studies. The high risk of bias in many of our included studies reflects a general need for more rigorous experimental standards in neonatal pain studies. Furthermore, the neonatal pain research community is increasingly aware of the limitations of the highly subjective pain scoring systems used in experimental and clinical settings; whether these tools better quantify distress in neonates as a distinct entity to nociception is increasingly being discussed.^6^, ^125^ Nevertheless, a reduction in neonatal distress is of obvious intrinsic value. The NPGG therefore regarded data showing meaningful improvements in these experimental measures of pain to also be of clinical significance.

The systematic review had some inherent limitations. Our inclusion and exclusion criteria were dictated by our aim of developing guidelines on procedure-related pain in non-tertiary settings in low-income and middle-income countries. These criteria might have therefore resulted in the omission of certain studies relating to particular analgesic strategies, as reflected by some differences in the number of included studies in our and others' reviews.^8^, ^9^ Furthermore, only 14 studies assessed pharmacological analgesic options. Given the adverse side-effect profiles of some of these interventions, scarcity of evidence from our review for their benefit, as well as frequent lack of availability in low-income and middle-income countries, the NPGG chose not to recommend these pharmacological analgesics for use during routine procedures. In high-income health-care settings, guidelines might be able to more safely recommend pharmacological analgesics, when increased resources facilitate safer use of such interventions. Additionally, we were limited in our ability to include all studies in our meta-analysis. However, we adhered to a thorough and transparent method of presenting our integrated narrative and quantitative findings to the guideline development panel. We found that certain procedures were done significantly more frequently than others were, with heel prick being the most frequently observed. However, we and others^87^ contest that the pain stimulus generated by a heel prick is similar to that of our other included procedures, meaning that our findings are generalisable to similar acutely painful procedures. We also detected a high degree of heterogeneity in some meta-analyses. However, we were limited in our ability to fully investigate possible sources of this because of the small number of studies within many comparisons. A final limitation of our review might be an underappreciated publication bias for certain comparisons. Our analyses for small-study effects were limited to comparisons of oral sugar versus placebo or no intervention, in which we identified a possible bias in those studies using PIPP as an outcome measure.

Despite increasing attention devoted to the study of pain in neonates, existing clinical guidelines are rarely employed successfully in clinical practice.^126^ This disparity might be due to the poor methodological quality of many guidelines, lack of interprofessional collaboration, and insufficient consideration for the context to which the guidelines are being applied.^127^ We have provided a rigorous systematic review, using both a narrative and a meta-analytical approach, of the evidence relating to a wide range of routine procedures and analgesics, and described the practical translation of the findings into a pragmatic and workable clinical guideline. Effective change to clinical practice in the low-income and middle-income health-care setting requires consideration of local social, financial, and organisational factors.^128^ We, and others,^129^ have shown the effectiveness of the GRADE framework in making these context-specific considerations explicit, thereby increasing the transparency of the translation of evidence to clinical recommendations. The NPGG were able to consider the likely values and preferences of Kenyan families and medical staff, local feasibility of interventions, and knowledge of how to obtain meaningful change in service delivery in the local health-care system. This process might lead to different national groups reaching different conclusions that are based on the same evidence, emphasising the importance of global progress in evidence synthesis and translation through nationally led guideline development processes.

The strength of our guideline development process lay in empowering a panel of local experts to make evidence-based recommendations for the local health-care system. The recommendations stemming from our study have since been adopted and included in new national guidelines by the Ministry of Health and partners in Kenya, thereby addressing a national gap in clinical recommendations, and are likely to lead to substantial improvements in neonatal care throughout the country. The efficient use of high-quality systematic reviews linked to structured and transparent processes to develop local guidelines represents an effective model for achieving meaningful improvements in the care of patients in the low-income and middle-income setting.
