# Supplementary material for: Development of locally relevant clinical guidelines for procedure-related neonatal analgesic practice in Kenya: a systematic review and meta-analysis
Source: Lancet Child Adolesc Health. 2020 Oct;4(10):750–60. doi: 10.1016/S2352-4642(20)30182-6 (PMC7507957; doi:10.1016/S2352-4642(20)30182-6)
Supplement: Supplementary appendix [file mmc1.pdf]

THE LANCET
Child & Adolescent Health

Supplementary appendix

This appendix formed part of the original submission and has been peer reviewed. We post it as supplied by the authors.

Supplement to: Wade C, Frazer JS, Qian E, et al. Development of locally relevant clinical guidelines for procedure-related neonatal analgesic practice in Kenya: a systematic review and meta-analysis. *Lancet Child Adolesc Health* 2020; published online July 28. http://dx.doi.org/10.1016/S2352-4642(20)30182-6.

Supplementary Table 1. Search strategy for MEDLINE*

1	INFANT,NEWBORN/
2	INFANT,PREMATURE/
3	(neonate* or newborn* or infant* or baby or babies).ab,ti.
4	1 or 2 or 3
5	ANALGESIA/
6	ANALGESICS/
7	CONSCIOUS SEDATION/
8	DEEP SEDATION/
9	PAIN MANAGEMENT/
10	PAIN,PROCEDURAL/
11	PAIN,POSTOPERATIVE/
12	(pain or analges* or sedat*).ab,ti.
13	("oral sucrose" or "oral dextrose" or "oral glucose").ab,ti.
14	("breast milk" or breastfeed*).ab,ti.
15	(sucking or pacifier*).ab,ti.
16	("skin to skin" or Kangaroo).ab,ti.
17	"swaddl*".ab,ti.
18	(music or singing or lullaby).ab,ti.
19	(topical and local).ab,ti.
20	(paracetamol or opioid* or ketamine or ibuprofen).ab,ti.
21	("dorsal penile nerve block*" or DPNB*)
22	5 or 6 or 7 or 8 or 9 or 10 or 11 or 12 or 13 or 14 or 15 or 16 or 17 or 18 or 19 or 20 or 21
23	(procedure* or procedural or intervention*).ab,ti.
24	(invasive or painful).ab,ti.
25	23 and 24
26	("heel prick*" or "heel stick*" or lancing).ab,ti.
27	(venepuncture* or venipuncture*).ab,ti.
28	"cannulation*".ab,ti.
29	"injection*".ab,ti.
30	(intramuscular or subcutaneous).ab,ti.
31	29 and 30
32	(catheterisation or catheterization).ab,ti.
33	"lumbar puncture* ".ab,ti.
34	prongs.ab,ti.
35	(abscess* and drain*).ab,ti.
36	("chest drain*" and insert*).ab,ti.
37	("chest drain*" and remov*).ab,ti.
38	(clubfoot or "club foot" or talipes).ab,ti.
39	(manipulat* or casting or Ponseti).ab,ti.
40	38 and 39
41	"circumcision*".ab,ti.
42	("gastric suction*" or "nasogastric suction*").ab,ti.
43	("total parenteral " or TPN).ab,ti.
44	"central venous catheter* ".ab,ti.
45	"necroti* enterocolitis ".ab,ti.
46	42 or 43 or 44
47	45 and 46
48	25 or 26 or 27 or 28 or 31 or 32 or 33 or 34 or 35 or 36 or 37 or 40 or 41 or 47
49	4 and 22 and 48
50	limit 49 to animals
51	49 not 50

*Search strategies for Embase, CENTRAL and CINAHL were adapted from this strategy

Supplementary Table 2. List of comparisons included in meta-analysis

Analgesic	Comparator
Heart Rate*	
Breastfeeding	Placebo or no intervention
Expressed breast milk	Placebo or no intervention
Local anaesthesia	Placebo or no intervention
Music	Placebo or no intervention
Non-nutritive sucking	Placebo or no intervention
Non-nutritive sucking	Oral Sugar
Skin-to-skin	Skin-to-skin plus breastfeeding
Skin-to-skin	Placebo or no intervention
Oral Sugar	Placebo or no intervention
Swaddling	Placebo or no intervention
Oxygen Saturation*	
Breastfeeding	Placebo or no intervention
Expressed breast milk	Placebo or no intervention
Local anaesthesia	Placebo or no intervention
Music	Placebo or no intervention
Non-nutritive sucking	Placebo or no intervention
Non-nutritive sucking	Oral Sugar
Skin-to-skin	Placebo or no intervention
Oral Sugar	Placebo or no intervention
Premature Infant Pain Profile (PIPP)*	
Breastfeeding	Placebo or no intervention
Breastfeeding	Oral Sugar
Expressed breast milk	Placebo or no intervention
Expressed breast milk	Oral Sugar
Local anaesthesia	Placebo or no intervention
Non-nutritive sucking	Placebo or no intervention
Skin-to-skin	Placebo or no intervention
Skin-to-skin	Oral Sugar
Skin-to-skin	Swaddling
Oral Sugar	Placebo or no intervention
Neonatal Infant Pain Scale (NIPS)*	
Breastfeeding	Placebo or no intervention
Breastfeeding	Skin-to-skin
Breastfeeding	Oral Sugar
Non-nutritive sucking	Oral Sugar
Skin-to-skin	Oral Sugar
Oral Sugar	Placebo or no intervention
Swaddling	Placebo or no intervention
Neonatal Facial Coding System (NFCS)*	
Expressed breast milk	Placebo or no intervention
Expressed breast milk	Oral Sugar
Skin-to-skin	Placebo or no intervention
Oral Sugar	Placebo or no intervention
Local anaesthesia	Placebo or no intervention
Douleur Aiguë du Nouveau-né scale (DAN)*	
Expressed breast milk	Placebo or no intervention
Non-nutritive sucking	Placebo or no intervention
Non-nutritive sucking	Oral Sugar

* Outcome measure used for these comparisons

Supplementary Table 3. Study characteristics table of all 149 included studies

Study	n	Country	Methods	Procedure	Groups	Outcome measure	Age (days) at procedure (mean[SD])*
Abad 1996[42]	28	Spain	RCT, PG	VP	OS, Placebo	HR, SpO_2	6·5[3·1]
Abad 2001[48]	47	Spain	RCT, PG	VP	OS, LA, OS+LA, Placebo	HR, SpO_2	1·9[0·5]
Acharya 1998[130]	19	UK	RCT, PG	VP	LA, Placebo	HR, SpO_2, NFCS	27·5[15·5]
Acharya 2004[49]	39	UK	RCT, XG	VP	OS, Placebo	HR, SpO_2, NFCS	27·2[24·4]
Ahuja 2000[50]	25	India	RCT, XG	IMI	OS, Placebo, No intervention	HR, SpO_2, NFCS	3·5
Akcam 2004[51]	34	Turkey	RCT, XG	HP	OS, Placebo	DAN	4[2·75]
Akcam 2004[52]	60	Turkey	RCT, XG	HP	OS, Placebo	DAN	4·5[4·5]
Altun-Köroğlu 2010[53]	75	Turkey	RCT, PG	HP	OS, EBM, Placebo	NFCS	6·4
Aydin 2019[22]	100	Turkey	RCT, PG	HP	BF, No intervention	NIPS	..
Badiee 2009[131]	72	Iran	RCT, PG	HP	PCM, Placebo	HR, SpO_2, PIPP	3·0[0·6]
Basnet 2010[54]	50	Nepal	RCT, PG	VP	OS, No intervention	DAN	..
Bauer 2004[55]	58	Germany	RCT, PG	VP	OS, Placebo	HR, PIPP	3·2[0·8]
Beken 2014[132]	25	Turkey	RCT, PG	VP	OS+NNS, NNS+Placebo	NIPS	..
Bellieni 2001[56]	17	Italy	RCT, XG	HP	OS, OS+NNS, Placebo, No intervention	PIPP	..
Bellieni 2002[92]	79	Italy	RCT, PG	HP	OS, NNS, OS+NNS, No intervention	DAN	..
Bellieni 2013[133]	62	Italy	RCT, XG	IMI	OS, LA	DAN	..
Bembich 2018[34]	80	Italy	RCT, PG	HP	OS, BF, EBM, OS+Holding	NIPS	..
Biran 2011[134]	76	France	RCT, PG	VP	OS+LA, OS+Placebo	PIPP, DAN	15·7[9·8]
Blass 1999[57]	40	USA	RCT, PG	HP	OS, NNS+Placebo, NNS+OS, Placebo	HR	..
Brovedani 2007[38]	146	Italy	RCT, PG	HP	OS, BF, SW	PIPP	..
Bucher 1995[135]	16	Switzerland	RCT, XG	HP	OS, Placebo	HR, SpO_2	..
Bueno 2012[100]	88	Brazil	RCT, PG	HP	OS, EBM	PIPP	..
Butt 2000[136]	14	Canada	RCT, XG	HP	M, No intervention	HR, SpO_2	8·2[3·1]
Campos 1989[137]	32	USA	RCT, PG	HP	NNS, SW	HR	..
Carbajal 1999[58]	150	France	RCT, PG	VP	OS, NNS, OS+NNS, Placebo, No intervention	DAN	3·6[0·4]
Carbajal 2002[59]	39	France	RCT, XG	SC	OS, OS+NNS, Placebo	DAN	26·2[6·1]
Carbajal 2003[23]	179	France	RCT, PG	VP	OS+NNS, BF, Placebo, No intervention	NIPS, DAN	3[0·4]
Cardoso 2014[93]	80	Brazil	RCT, PG	AP	OS, M, OS+M	PIPP	4
Castral 2008[104]	60	Brazil	RCT, PG	HP	SS, No intervention	HR, NFCS	..
Chermont 2009[94]	640	Brazil	RCT, PG	IMI	OS, SS, OS+SS, Placebo	PIPP, NIPS, NFCS	1·2[0·3]
Chiabi 2016[35]	100	Cameroon	RCT, PG	HP	OS, BF	NIPS	2·3[0·3]
Codipietro 2008[36]	101	Italy	RCT, PG	HP	OS, BF	HR, SpO_2, PIPP	3·4[0·5]
Collados-Gomez 2018[138]	66	Spain	RCT, XG	VP	OS+NNS+SW, EBM+NNS+SW	PIPP	9·1[2·0]
Cong 2009[105]	14	USA	RCT, XG	HP	SS, No intervention	HR	6·0[1·0]
Cong 2011[106]	28	USA	RCT, XG	HP	SS (30 minutes, 80 minutes), No intervention	PIPP	5·2[0·9]
Cong 2012[107]	26	USA	RCT, XG	HP	SS (15 minutes, 30 minutes), No intervention	HR	13·9[5·8]
Cook 2017[95]	40	USA	RCT, PG	PICC	OS, Placebo	HR, SpO_2, PIPP	..
Corbo 2000[122]	24	Italy	RCT, XG	HP	NNS, No intervention	HR	4·7[1·9]
De Bernardo 2019[139]	66	Italy	RCT, PG	VP	OS (10%, 24%)+NNS	HR, SpO_2, NIPS	22·9[4·9]
De Melo 2017[140]	28	Brazil	RCT, PG	AP	OS, M	NFCS	..
Deshmukh 2002[60]	60	India	RCT, PG	VP	OS (10%, 25%), Placebo	HR, SpO_2	7·1[1·8]
Dezhdar 2016[43]	82	Iran	RCT, PG	VP	SS, SW, No intervention	HR, PIPP	..
Elserafy 2009[61]	36	Saudi Arabia	RCT, PG	VP	OS, NNS, NNS+Placebo (water), NNS+OS, Placebo (water), No intervention	HR, SpO_2, PIPP	..
Eriksson 1999[62]	120	Sweden	RCT, PG	VP	OS, No intervention	PIPP	..
Eriksson 2004[63]	43	Sweden	RCT, PG	HP	OS, Placebo	PIPP	..

Erkut 2017[141]	74	Turkey	RCT, PG	HP	SW, No intervention	HR, SpO_2, PIPP, NIPS	‥
Fallah 2017[41]	120	Iran	RCT, PG	IMI	BF, SS, SW	NIPS	‥
Field 1984[118]	96	USA	RCT, PG	HP	NNS, No intervention	HR	7·0[3·4]
Gajbhiye 2018[24]	150	India	RCT, PG	IMI	OS, BF, No intervention	HR, SpO_2, PIPP	1·8[0·2]
Gao 2015[117]	76	China	RCT, PG	HP	SS, No intervention	HR	4·3[0·2]
Gao 2018[64]	86	China	RCT, PG	HP	OS, NNS, OS+NNS, No intervention	HR, SpO_2, PIPP	5·1[0·2]
Gerull 2013[142]	25	Switzerland	RCT, PG	HP	OS, SW, OS+SW	HR	‥
Gharehbaghi 2007[65]	60	Iran	RCT, PG	VP	OS, Placebo	HR	5·6[1·1]
Gibbins 2002[143]	190	Canada	RCT, PG	HP	OS, OS+NNS, NNS+Placebo(water)	PIPP	‥
Golestan 2007[96]	60	Iran	RCT, PG	IMI	OS, Placebo, No intervention	HR	0·6[0·1]
Gormally 2001[97]	85	Canada	RCT, PG	HP	OS, OS+Holding, Placebo (water)+Holding, No intervention	HR	2·3[0·3]
Gradin 2002[144]	196	Sweden	RCT, PG	VP	OS+Placebo (cream), LA+Placebo (water)	HR, PIPP	4·3[3·3]
Gradin 2004[66]	79	Sweden	RCT, PG	VP	OS, OS+BF, BF+Placebo, Placebo	PIPP	‥
Gray 2000[108]	30	USA	RCT, PG	HP	SS, No intervention	HR	‥
Gray 2002[25]	30	USA	RCT, PG	HP	BF, No intervention	HR	‥
Gray 2012[98]	45	USA	RCT, PG	VC	OS, NNS, No intervention	HR	‥
Haouari 1995[44]	60	United Kingdom	RCT, PG	HP	OS (12.5%, 25%, 50%), Placebo	HR	3·5[0·6]
Harrison 2003[67]	128	Australia	RCT, PG	HP	OS, Placebo	NFCS	18·8[13·3]
Hashemi 2016[26]	131	Iran	RCT, PG	IMI	BF, SW, BF+SW, No intervention	HR, SpO_2, NFCS	1·7[0·8]
Hatami Bavarsad 2018[27]	75	Iran	RCT, PG	IMI	BF, EBM, No intervention	HR, SpO_2, DAN	‥
Ho 2016[145]	54	Hong Kong	RCT, PG	HP	SW, No intervention	HR, SpO_2, PIPP	9·9[7·6]
Holsti 2011[146]	57	Canada	RCT, PG	HP	BF, NNS	HR	9
Hsieh 2018[99]	20	Taiwan	RCT, XG	HP	OS, EBM, Placebo, No intervention	PIPP	‥
Huang 2004[147]	32	Taiwan	RCT, XG	HP	SW, No intervention	HR, SpO_2, PIPP	7·5[7·1]
Jain 2000[148]	39	United Kingdom	RCT, PG	VP	LA, Placebo	NFCS	7·0[3·8]
Jatana 2003[45]	125	India	RCT, PG	HP	OS, EBM, Placebo	HR, SpO_2	‥
Johnston 1997[68]	87	Canada	RCT, PG	HP	OS, SW+Rocking, OS+SW+Rocking, Placebo (water)	HR	5·7[1·2]
Johnston 1999[69]	47	Canada	RCT, PG	HP	OS, Placebo	PIPP	6·5[1·6]
Johnston 2003[109]	74	Canada	RCT, XG	HP	SS, No intervention	HR, SpO_2, PIPP	‥
Johnston 2008[149]	61	Canada	RCT, XG	HP	SS, SW	PIPP	‥
Kashaninia 2008[110]	100	Iran	RCT, PG	IMI	SS, No intervention	NIPS	0·08
Kaur 2003[150]	60	India	RCT, PG	LP	LA, Placebo	HR, SpO_2, NFCS	3·4
Kristoffersen 2018[151]	53	Norway, South Africa	RCT, XG	VP	OS (0.2 mL, 0.5 mL)	PIPP	‥
Kurdahi Badr 2017[152]	126	Lebanon	RCT, XG	HP	M, No intervention	HR, SpO_2, PIPP	‥
Larsson 1998[153]	111	Sweden	RCT, PG	VP	LA, No intervention	NFCS	5·0[0·8]
Leite 2009[28]	60	Brazil	RCT, PG	HP	BF, No intervention	HR, SpO_2, NFCS	‥
Lemyre 2007[154]	137	Canada	RCT, PG	VP	OS+LA+NNS+SW, OS+Placebo (cream)+NNS+SW	PIPP	6·7[2·2]
Leng 2016[155]	671	China	RCT, PG	HP	OS, OS+NNS, OS+SW, OS+NNS+SW	HR, SpO_2, NIPP, NFCS	‥
Liaw 2010[119]	104	Taipei , USA	RCT, PG	HP	NNS, No intervention	PIPP	6·4[2·0]
Liaw 2011[70]	165	Taiwan	RCT, PG	IMI	OS, NNS, No intervention	HR, NFCS	2·5[0·2]
Liaw 2012[120]	34	Taiwan	RCT, XG	HP	NNS, SW, No intervention	PIPP	7·0[5·0]
Lima 2013[29]	64	Brazil	RCT, PG	VP	BF, NNS, No intervention	NIPS	‥
Lima 2017[101]	78	Brazil	RCT, PG	IMI	OS, NNS	HR, SpO_2, NIPS	0·8[0·2]
Lindh 2000[156]	56	Sweden	RCT, PG	VP	LA, Placebo	HR	3·4[0·4]
Liu 2010[71]	105	Taipei	RCT, PG	VP	OS, NNS, No intervention	NIPS	3·0[0·6]
Liu 2015[111]	40	China	RCT, PG	HP	SS, No intervention	HR, SpO_2	‥

Long 2003[157]	32	Northern Ireland	RCT, PG	VP	LA, Placebo	NFCS	6·9[1·2]
Ludington-Hoe 2005[112]	23	USA	RCT, XG	HP	SS, No intervention	HR, SpO_2	22[11·4]
Marcatto 2011[158]	30	Brazil	RCT, PG	PICC	OS+LA, OS+Placebo (cream), LA+Placebo (water)	HR, NIPS	..
Marin Gabriel 2013[159]	127	Spain	RCT, PG	HP	OS, SS, OS+SS, BF+SS	HR, SpO_2, NIPS	..
Marofi 2015[160]	50	Iran	RCT, PG	HP	M, No intervention	HR, SpO_2, PIPP	..
Mathai 2006[72]	70	India	RCT, PG	HP	OS, EBM, NNS, Placebo	DAN	1·9[0·01]
Milazzo 2011[73]	47	USA	RCT, PG	AP	OS, No intervention	HR, SpO_2, NIPS	..
Mirzarahimi 2013[121]	60	Iran	RCT, PG	HP	NNS, No intervention	HR, SpO_2, PIPP	..
Morrow 2010[161]	42	USA	RCT, PG	HP	SW, No intervention	NIPS	..
Mosayebi 2014[162]	64	Iran	RCT, XG	HP	SS, SS+SW	PIPP	7·3[3·7]
Nimbalkar 2013[113]	47	India	RCT, XG	HP	SS, No intervention	PIPP	..
Noori Shadkam 2008[163]	220	Iran	RCT, PG	VP	OS+Placebo (cream), LA+Placebo (water)	NIPS	4·1
Obeidat 2015[30]	128	Jordan	RCT, PG	HP	BF, No intervention	PIPP	5·8[0·3]
Ogawa 2005[74]	50	Japan	RCT, PG	VP, HP	OS, Placebo	NFCS	..
Okan 2007[75]	93	Turkey	RCT, XG	HP	OS, Placebo	HR, SpO_2, NFCS	..
Okan 2010[114]	107	Turkey	RCT, PG	HP	SS, BF+SS, Placebo	HR, SpO_2, NFCS	1·4[0·1]
Olsson 2016[115]	10	Sweden	RCT, XG	VP	SS, No intervention	HR, SpO_2, PIPP	6·6[4·7]
Ors 1999[76]	102	Turkey	RCT, PG	HP	OS, EBM, Placebo	HR	1·4[1·6]
Ou-Yang 2013[77]	123	Taiwan	RCT, PG	HP	OS, EBM, Placebo	HR, SpO_2, PIPP	..
Overgaard 1999[78]	96	Denmark	RCT, PG	HP	OS, Placebo	NIPS	..
Ozdogan 2010[79]	142	Turkey	RCT, PG	HP	OS (single, double dose), EBM (single, double dose), Placebo (double, single dose)	NFCS	2·2[0·2]
Patel 2003[164]	10	Canada	RCT, XG	HP	LA, Placebo	PIPP, NIPS	2·8[1·4]
Peng 2018[165]	109	Taiwan	RCT, PG	HP	NNS+EBM, NNS+EBM+SW, No intervention	PIPP	13·1[6·6]
Ramenghi 1996[47]	60	UK	RCT, XG	HP	OS (25%, 50%, sweetener), Placebo	HR	0·3[0·2]
Ramenghi 1996[80]	15	United Kingdom	RCT, XG	HP	OS, Placebo	HR	8·7[6·5]
Rawal 2018[81]	63	India	RCT, PG	HP	OS, EBM, Placebo	HR, SpO_2, PIPP	3·1[0·7]
Rioualen 2018[40]	102	France	RCT, PG	VP	OS, BF	NFCS	2·9[0·5]
Rogers 2006[82]	33	USA	RCT, PG	BC	OS, Placebo	DAN	..
Rossi 2018[166]	120	Italy	RCT, PG	IMI, HP	M (Mozarts, Beethoven, heartbeat sounds), No intervention	HR, SpO_2, NIPS	..
Saeidi 2011[116]	60	Iran	RCT, PG	IMI	SS, No intervention	SpO2, NIPS	..
Sahoo 2013[83]	160	India	RCT, PG	VP	OS, EBM, Placebo	HR, SpO_2, PIPP	3·3[0·8]
Sajedi 2006[84]	40	Iran	RCT, PG	IMI	OS, Placebo	HR, NIPS	..
Shabani 2016[167]	20	Iran	RCT, XG	AP	M, No intervention	HR, SpO_2, NFCS	..
Shah 2017[168]	35	Australia	RCT, XG	HP	OS, M, OS+M	HR, SpO_2, PIPP	4·0[6·0]
Shu 2014[169]	50	Taiwan	RCT, PG	HP	SW, No intervention	HR, SpO_2, PIPP, NIPS	1·9[1·0]
Shukla 2018[170]	100	India	RCT, PG	HP	OS, SS	PIPP	14·04[11·10]
Shukla 2018[171]	200	India	RCT, PG	HP	EBM, SS+EBM, SS+EBM+M, M+EBM	PIPP	8·2[7·4]
Simonse 2012[39]	70	Netherlands	RCT, PG	HP	OS, BF, EBM	PIPP	..
Singh 2017[31]	80	India	RCT, PG	HP	BF, No intervention	HR, SpO_2	..
Skogsdal 1997[46]	120	Sweden	RCT, PG	HP	OS, EBM, No intervention	HR, SpO_2	5·4[4·9]
Slater 2010[85]	44	United Kingdom	RCT, PG	HP	OS, Placebo	PIPP	3·0[2·0]
Soliman 2016[172]	60	Egypt	RCT, PG	CPAP	LA, No intervention	PIPP	4·3[2·9]
Soltani 2018[37]	161	Iran	RCT, PG	HP	OS, BF, SS, LA	NIPS	..
Stevens 1999[173]	122	Canada	RCT, XG	HP	OS+NNS, NNS+placebo (water), No intervention	PIPP	..
Stevens 1999[174]	106	Canada	RCT, XG	HP	LA, Placebo	HR, SpO_2, PIPP	4·1[0·6]
Stevens 2018[175]	236	Canada	RCT, PG	HP	OS (0.1 mL, 0.5 mL, 1.0 mL)+NNS	PIPP	8·3

Suhrabi 2014[86]	90	Iran	RCT, PG	IMI	OS (sucrose, glucose), No intervention	NIPS	..
Sujatha 2017[176]	155	India	RCT, PG	IMI	SW, OS+SW	SpO_2, NIPS	..
Taddio 2008[87]	240	Canada	RCT, PG	IMI, VP, HP	OS, Placebo	PIPP	0·04[0·01]
Taddio 2011[177]	321	Canada	RCT, PG	VP	OS+LA, LA+Placebo (water), OS+Placebo (cream)	HR, SpO_2	..
Thakkar 2016[88]	180	India	RCT, PG	HP	OS, NNS, OS+NNS, No intervention	PIPP	..
Tutag Lehr 2015[89]	56	USA	RCT, PG	HP	OS, Placebo	NIPS	..
Upadhyay 2004[102]	81	India	RCT, PG	VP	EBM, Placebo	HR, SpO_2, NFCS	8·8[5·0]
Uyan 2005[103]	62	Turkey	RCT, PG	HP	EBM (foremilk, hind milk), Placebo	HR	6·3[1·1]
Uzelli 2015[90]	80	Turkey	RCT, PG	IMI	OS, No intervention	SpO2, NIPS	22·3[0·7]
Yilmaz 2011[91]	120	Turkey	RCT, PG	HP	OS, EBM, NNS, No intervention	HR, NIPS	3·4[0·4]
Zargham-Boroujeni 2017[32]	75	Iran	RCT, PG	VP	BF, No intervention	NIPS	..
Zhu 2015[33]	250	China	RCT, PG	HP	BF, M, BF+M, No intervention	NIPS	3·3[0·3]

PG = parallel groups, XG - crossover groups, RCT = randomised controlled trials, PG = parallel groups, XG = crossover groups, VP = venepuncture, HP = heel prick, IMI = IM injection, LP = lumbar puncture, PICC = PICC line insertion, CPAP = CPAP prongs insertion, BC = bladder catheterisation, SC = subcutaneous injection, VC = vaccination, OS = oral sugar, BF = breastfeeding, EBM = expressed breast milk, NNS = non-nutritive sucking, SS = skin-to-skin, SW = swaddling, LA = topical local anaesthetic, PCM = paracetamol, M = music

*Reported where available, or where available data could be converted to a mean and standard deviation for the study population using previously described methodology.[1–3]

1. Hozo S.P., Djulbegovic B, Hozo I. Estimating the mean and variance from the median, range, and the size of a sample. BMC Med Res Methodol. 2005;**5,**13

2. Wan X, Wang W, Liu J, Tong T. Estimating the sample mean and standard deviation from the sample size, median, range and/or interquartile range. BMC Med Res Methodol. 2014;**14**:135

3. Bornstein M, Hedges L.V, Higgins J.P.T, Rothstein H. (2009) "Introduction to Meta-Analysis" Print ISBN:9780470057247 Copyright © 2009 John Wiley & Sons, Ltd

Supplementary Table 4: Panel's GRADE summary of evidence table for PICO questions discussed in Results

**PICO:**<br>**In neonates ≤ 28 days, which of the following is superior in reducing pain during routine procedures?**	**Studies**	**Neonates**	**Bias**	**Inconsistency**	**Indirectness**	**Imprecision**	**Summary**	**Certainty†**
BF versus placebo/no intervention	12	991					BF is superior to placebo/no intervention**	⊕⊕⊕⊕
BF versus OS	8	670					BF is superior to OS**	⊕⊕◯◯
BF versus EBM	3	136					BF is superior to EBM	⊕⊕⊕◯
BF versus SS	2	160					BF is superior to SS**	⊕⊕⊕◯
EBM versus placebo/no intervention	14	863					EBM is superior to placebo/no intervention **	⊕⊕⊕◯
EBM versus OS	14	920					EBM is inferior to OS ≥10%.	⊕⊕⊕◯
OS versus placebo/no intervention	58	3948					OS is superior to placebo/no intervention**	⊕⊕⊕⊕
OS versus NNS	11	645					OS is superior to NNS	⊕⊕◯◯
OS versus OS with NNS	10	837					OS is inferior to OS with NNS	⊕⊕⊕◯
OS: concentration ≥24% versus <24%[1]	6*	453					OS ≥24% is superior to <24% solution	⊕⊕⊕◯
OS: concentration 24-25% versus 50%[1]	3*	110					OS 24-25% may not be inferior OS 50%	⊕◯◯◯
OS: volume ≤2 mL versus >2 mL[2]	*	..					Volumes >2 mL do not increase efficacy	⊕⊕⊕◯
Sucrose, glucose, dextrose, fructose versus sweetener[3]	*	..					None are clearly superior to the others	⊕⊕⊕◯
SS versus placebo/no intervention	16	1054					SS is superior to placebo/no intervention**	⊕⊕⊕⊕
NNS versus placebo/no intervention	16	932					NNS is superior to placebo/no intervention **	⊕⊕⊕◯
NNS versus NNS with OS	4	192					NNS is inferior to NNS with OS**	⊕⊕⊕⊕

BF: Breastfeeding; OS: Oral sugar; EBM: Expressed breastmilk; NNS: Non-nutritive sucking; SS: Skin-to-skin

Notes:
1. Oral sugar concentration: range = 5-50%, median: 25%. The concentrations directly compared varied. We grouped according to <24%, ≥24% and 50%.
2. Volume: range= 0.05-5 mL, median=2 mL
3. Sucrose (number of studies (#) = 52), glucose (#=33), dextrose (#=8), sweetener (#=2) fructose (#=1))

* No/few direct comparative studies, therefore indirect analysis across whole data set performed.
** Quantitative analysis of any one of the outcome measures supports conclusion with 95% confidence

† The GRADE framework also allows for "Other Considerations" to determine the overall certainty in effect estimate.

	Risk of bias			Certainty of conclusion using GRADE analysis			
KEY	Not serious	Serious	Very serious	⊕⊕⊕⊕ **High**	⊕⊕⊕◯ **Moderate**	⊕⊕◯◯ **Low**	⊕◯◯◯ **Very low**

Supplementary Table 5. Summary of narrative synthesis findings with associated references showing superiority, equivalence or inferiority of the analgesic versus the comparator as described in the Results

PICO		Analgesic versus Comparator		
**Analgesic**	**Comparator**	**Superior**	**Equivalence**	**Inferior**
Breastfeeding	Placebo or no intervention	22,23,24,25,26,27,28,29,30,31,32,33	..	..
Breastfeeding	Oral sugar	24,34,35,36,37	38,39	40
Breastfeeding	Expressed breast milk	27,34	..	39
Breastfeeding	Skin-to-skin	37,41	..	..
Oral sugar ≥24%	Oral sugar <24%	42,43,44,45,46	..	47
Oral sugar	Placebo or no intervention	24,42,44,45,46,47,48,49,50,51,52,53,54,55,56,57,58, 59,60,61,62,63,64,65,66,67,68,69,70,71,72,73,74,75, 76,77,78,79,80,81,82,83,84,85,86,87,88,89,90,91	92,93,94,95, 96,97,98,99	..
Oral sugar	Expressed breast milk	45,46,76,77,79,81,83,91,100	34,39,53,72,99	
Oral sugar	Non-nutritive sucking	91,101	61,64,70,88,92,98	58,71,72
Expressed breast milk	Placebo or no intervention	45,53,76,77,81,83,91,99,102	27,46,72,79,103	..
Skin-to-skin	Placebo or no intervention	43,94,104,105,106,107,108,109,110,111,112,113,114 ,115,116,	117	..
Non-nutritive sucking	Placebo or no intervention	29,56,58,61,64,70,71,72,88,91,98,118,119,120,121	122	..

Supplementary Table 6. Panel's GRADE summary of evidence table for PICOs not discussed in Results

**PICO:** **In neonates ≤ 28 days, which of the following is superior in reducing pain during routine, acutely painful procedures?**	**Studies**	**Neonates**	**Bias**	**Inconsistency**	**Indirectness**	**Imprecision**	**Summary**	**Certainty†**
BF versus swaddling	3	243					BF may not be inferior to swaddling	⊕○○○
BF versus BF with music	1	126					BF is not inferior to BF with music	⊕⊕○○
Sugar versus skin to skin	4	561					Sugar may be inferior to skin-to-skin**	⊕○○○
Sugar versus LA	7	880					Sugar is superior to LA	⊕⊕⊕⊕
Sugar versus sugar with skin-to-skin	2	387					Sugar is inferior to sugar with skin-to-skin	⊕⊕○○
Sugar versus sugar with LA	4	335					Sugar is not inferior to sugar with LA	⊕⊕⊕○
Sugar versus sugar with music	2	115					Sugar is inferior to sugar with music	⊕⊕○○
Skin-to-skin versus swaddling	3	255					Skin-to-skin is no different than swaddling**	⊕○○○
NNS versus swaddling	2	101					NNS is no different than swaddling	⊕○○○
LA versus placebo/no intervention	11	656					LA is not superior to placebo/no intervention**	⊕⊕○○
LA versus LA with sugar	3	256					LA is not inferior to LA with sugar	⊕⊕⊕○
Swaddling versus placebo/no intervention	8	410					Swaddling is not superior to placebo/no intervention	⊕⊕○○
Swaddling versus swaddling with BF or NNS	2	197					Swaddling is not inferior to swaddling with BF or NNS	⊕○○○
Music versus placebo/no intervention	6	487					Music is not superior to placebo/no intervention**	⊕⊕○○

BF = breastfeeding, LA = topical local anaesthetics, NNS = non-nutritive sucking.
** Quantitative analysis of any of the outcome measures supports conclusion with 95% confidence threshold

† The GRADE framework also allows for "Other Considerations" to determine the overall certainty in effect estimate.

	Risk of bias			Certainty of conclusion using GRADE analysis			
KEY	Not serious	Serious	Very serious	⊕⊕⊕⊕ **High**	⊕⊕⊕○ **Moderate**	⊕⊕○○ **Low**	⊕○○○ **Very low**

Supplementary Table 7. Table counting the number of the 149 studies, which studied the following procedures, analgesics and outcome measures

**Procedures**	**n**	**Analgesics**	**n**	**Outcome measures**	**n**
Heel prick	88	Oral sugar	78	Heart rate	76
Venipuncture/cannula	40	Skin-to-skin	22	Transcutaneous oxygen saturation	49
IM/SC injection	19	Breast feeding	21	Premature infant pain profile (PIPP)	57
Arteripuncture	4	Expressed breast milk	19	Neonatal facial coding system (NFCS)	22
CPAP prongs insertion	1	Non-nutritive sucking	19	Neonatal infant pain scale (NIPS)	32
Urinary catheterisation	1	Topical local anaesthesia	13	Douleur Aigue du Nouveau-ne scale (DAN)	12
Lumbar puncture	1	Swaddling	15		
. .	. .	Music	9		
. .	. .	Paracetamol	1		

Supplementary Table 8. Table of each component judged for Cochrane risk of bias for each study included in analysis

Study	Sequence Generation	Allocation concealment	Blinding of participants and personnel	Blinding of outcome assessors	Incomplete outcome data	Selective outcome reporting	Other sources of bias	Overall judgement of study risk of bias
Abad 1996[42]	Low	Unclear	Unclear	Low	Low	Low	Low	Low
Abad 2001[48]	Low	Low	Low	Low	Low	Low	Low	Low
Acharya 1998[130]	Unclear	Unclear	Low	Low	Low	High	High	High
Acharya 2004[49]	Low	Low	Low	Low	Low	Low	Low	Low
Ahuja 2000[50]	High	High	High	Low	High	High	High	High
Akcam 2004[51]	Low	Low	Low	Low	Low	Unclear	Low	Low
Akcam 2004[52]	Low	Low	Low	Low	Low	Low	Low	Low
Altun-Koroglu 2010[53]	Low	Low	Low	Low	High	Low	Low	Low
Aydin 2019[22]	Low	Low	High	High	Low	Low	Low	Low
Badiee 2009[131]	Low	Low	Low	Low	Low	High	Low	Low
Basnet 2010[54]	Low	Low	Unclear	Low	Low	Low	Low	Low
Bauer 2004[55]	Low	Low	Low	Low	Low	High	High	Low
Beken 2014[132]	Low	Low	Low	Low	Low	Unclear	Low	Low
Bellieni 2001[56]	High	High	High	High	Unclear	High	Unclear	High
Bellieni 2002[92]	Low	Unclear	Unclear	Unclear	Low	Low	Low	Low
Bellieni 2013[133]	High	High	High	High	High	High	High	High
Bembich 2018[34]	Low	Unclear	High	High	Unclear	High	Low	High
Biran 2011[134]	Low	Low	Unclear	Low	Low	Low	Low	Low
Blass 1999[57]	High	High	High	Unclear	Unclear	Unclear	Unclear	High
Brovedani 2007[38]	High	High	High	High	High	High	High	High
Bucher 1995[135]	Low	Low	Low	Low	Unclear	Low	Low	Low
Bueno 2012[100]	Low	Unclear	Low	Low	Unclear	Low	Unclear	Low
Butt 2000[136]	Unclear	Unclear	High	Unclear	Low	Unclear	Unclear	High
Campos 1989[137]	High	High	High	Low	High	High	High	High
Carbajal 1999[58]	Low	Low	High	High	Low	Low	Low	Low
Carbajal 2002[59]	Low	Low	Low	Low	Low	High	High	High
Carbajal 2003[23]	Low	Low	Unclear	Low	Low	Low	Low	Low
Cardoso 2014[93]	Unclear	Low	High	Low	Low	High	Low	High
Castral 2008[104]	Low	Unclear	High	Low	High	Low	Low	High
Chermont 2009[94]	High	High	High	High	Low	Low	Low	High
Chiabi 2016[35]	High	High	High	High	Low	Low	High	High
Codipietro 2008[36]	Low	Low	High	High	Low	High	Low	High
Collados-Gomez 2018[138]	Low	Low	High	Low	Low	Unclear	Low	Low
Cong 2009[105]	Low	High	High	High	Low	Low	Low	High
Cong 2011[106]	High	Low	High	Unclear	High	Low	Low	High
Cong 2012[107]	Low	Low	High	Unclear	High	High	Low	High
Cook 2017[95]	Low	Low	Low	Low	Low	Low	High	Low
Corbo 2000[122]	Low	High	High	High	Low	Low	High	High
DeBernardo 2019[139]	Low	Unclear	Low	Low	Low	High	High	Low
DeMelo 2017[140]	High	Unclear	High	Low	Low	Low	High	High
Deshmukh 2002[60]	Unclear	Unclear	Low	Low	Low	Unclear	Low	Low
Dezhdar 2016[43]	Low	Unclear	High	Low	Low	Unclear	High	High
Elserafy 2009[61]	Unclear	Low	Low	Unclear	High	Low	Unclear	Low
Eriksson 1999[62]	Low	Low	High	Low	Low	Low	High	Low
Eriksson 2004[63]	Low	Low	Unclear	Low	High	Low	High	High
Erkut 2017[141]	Low	High	High	High	Low	Low	Low	High
Fallah 2017[41]	Low	Low	High	High	Low	Low	Low	High
Field 1984[118]	Unclear	Unclear	High	High	Low	High	High	High
Gajbhiye 2018[24]	Unclear	Unclear	High	High	Low	Low	Low	High
Gao 2015[117]	Low	Low	High	Low	Low	Low	Low	Low
Gao 2018[64]	Low	Unclear	High	Low	Low	Low	High	Low
Gerull 2013[142]	Low	Low	High	High	Low	High	High	High
Gharehbaghi 2007[65]	Unclear	Unclear	Unclear	Low	Low	Unclear	Low	Low
Gibbins 2002[143]	Low	Low	Low	Low	Low	Low	Unclear	Low
Golestan 2007[96]	High	Unclear	Unclear	Unclear	Unclear	Unclear	Unclear	High
Gormally 2001[97]	Unclear	Unclear	High	Low	Low	Low	Low	Low
Gradin 2002[144]	Low	Low	Low	Low	Low	Low	Low	Low
Gradin 2004[66]	Unclear	Low	Low	Low	High	Low	High	Low
Gray 2000[108]	High	Unclear	High	Low	Low	Low	Low	Low
Gray 2002[25]	Unclear	Unclear	High	Low	Low	Unclear	Low	High
Gray 2012[98]	High	Low	High	High	Low	Low	Low	High
Haouari 1995[44]	Unclear	Low	Low	Low	Low	Unclear	Low	Low
Harrison 2003[67]	Low	Low	Low	Low	Low	High	Low	Low
Hashemi 2016[26]	High	High	High	High	Low	High	High	High
HatamiBavarsad 2018[27]	High	High	High	High	Low	Low	High	High
Ho 2016[145]	Low	Low	High	High	Low	Low	Low	Low
Holsti 2011[146]	Low	Low	High	Low	Low	Low	High	Low
Hsieh 2018[99]	Unclear	Unclear	Unclear	Unclear	Low	Unclear	Unclear	High
Huang 2004[147]	Unclear	Unclear	Unclear	Unclear	High	Low	Low	High
Jain 2000[148]	Unclear	Low	Low	Low	Low	Unclear	Low	Low
Jatana 2003[45]	Unclear	Unclear	High	High	Low	High	Unclear	High
Johnston 1997[68]	Low	Unclear	Low	Unclear	Low	High	Low	Low
Johnston 1999[69]	Low	Low	Low	Low	Low	Unclear	Low	Low
Johnston 2003[109]	Low	Unclear	High	Low	Low	Low	Low	Low
Johnston 2008[149]	Low	Unclear	High	Low	Low	High	High	High
Kashaninia 2008[110]	Low	High	High	High	High	High	High	High
Kaur 2003[150]	Low	High	High	Low	Low	High	Low	Low
Kristoffersen 2018[151]	Low	Unclear	Unclear	Low	High	High	High	High
KurdahiBadr 2017[152]	High	Unclear	High	Low	High	High	High	High
Larsson 1998[153]	Unclear	Unclear	Low	Low	Low	High	Low	High
Leite 2009[28]	Low	Low	High	High	High	High	High	High
Lemyre 2007[154]	Low	Low	Low	Low	High	High	High	High
Leng 2016[155]	Low	Low	High	High	Low	Low	Low	Low
Liaw 2010[119]	Low	Unclear	High	Unclear	Low	Low	Low	Low
Liaw 2011[70]	Low	Low	High	High	Low	High	Low	High
Liaw 2012[120]	Low	Unclear	Low	Low	High	Unclear	High	High
Lima 2013[29]	Unclear	Unclear	High	Unclear	High	High	High	High
Lima 2017[101]	High	High	High	High	Low	High	Low	High
Lindh 2000[156]	Unclear	Unclear	Unclear	Unclear	High	Unclear	Unclear	High
Liu 2010[71]	Low	Unclear	Low	Low	Low	Low	Low	Low
Liu 2015[111]	Low	High	High	High	Low	Low	Low	Low
Long 2003[157]	Unclear	Low	Low	Low	Low	Low	Low	Low
Ludington-Hoe 2005[112]	Low	Low	High	Low	Low	Low	High	Low
Marcatto 2011[158]	Low	Unclear	Unclear	Low	Low	Low	High	Low
Marin Gabriel 2013[159]	Low	Unclear	Unclear	Unclear	High	Low	Low	Low
Marofi 2015[160]	High	High	High	Low	High	High	High	High
Mathai 2006[72]	Low	Unclear	Unclear	Unclear	Low	Unclear	Low	Low
Milazzo 2011[73]	Low	Low	High	Low	Low	High	High	High
Mirzarahimi 2013[121]	High	High	High	High	Low	Low	High	High
Morrow 2010[161]	High	High	High	High	High	High	High	High
Mosayebi 2014[162]	Unclear	Unclear	High	Low	Low	Low	High	High
Nimbalkar 2013[113]	Low	Low	High	Low	Unclear	Unclear	Low	Low
NooriShadkam 2008[163]	High	Unclear	Unclear	Unclear	Low	Low	Unclear	High
Obeidat 2015[30]	Unclear	Unclear	High	Low	Low	High	Low	High
Ogawa 2005[74]	Unclear	Low	Low	Low	Low	Unclear	Low	Low
Okan 2007[75]	Low	Low	Low	Low	Low	Low	High	Low
Okan 2010[114]	Unclear	Unclear	Low	Low	High	Low	Unclear	High
Olsson 2016[115]	High	High	High	High	Low	High	Unclear	High
Örs 1999[76]	Unclear	Unclear	Unclear	Low	High	High	Low	High
Ou-Yang 2013[77]	Low	Low	Low	Low	Low	Low	High	Low
Overgaard 1999[78]	Unclear	Low	Low	Low	Low	Low	Unclear	Low
Ozdogan 2010[79]	Unclear	Unclear	Unclear	Low	Low	Unclear	Unclear	High
Patel 2003[164]	Unclear	Unclear	Low	Low	Low	High	High	High
Peng 2018[165]	Low	Unclear	High	Low	Low	High	Low	High
Ramenghi 1996[47]	High	High	High	High	High	High	Low	High
Ramenghi 1996[80]	Unclear	Unclear	Unclear	Low	Low	Low	Unclear	Low
Rawal 2018[81]	Low	Low	Low	Low	Low	Low	Low	Low
Rioualen 2018[40]	Low	Unclear	High	Low	Low	Low	Low	Low
Rogers 2006[82]	High	Low	Low	Low	High	High	High	High
Rossi 2018[166]	Unclear	High	High	Unclear	Low	High	High	High
Saeidi 2011[116]	High	High	High	High	High	High	High	High
Sahoo 2013[83]	Low	Low	Low	Low	Unclear	Low	Unclear	Low
Sajedi 2006[84]	High	High	High	Low	High	High	Low	High
Shabani 2016[167]	High	High	High	Low	High	Low	High	High
Shah 2017[168]	Low	Low	High	Low	Low	Low	High	High
Shu 2014[169]	High	High	High	High	Low	High	Low	High
Shukla 2018[170]	Low	Low	High	Low	Low	Unclear	Unclear	Low
Shukla 2018[171]	Low	Low	High	Low	Low	Low	Unclear	Low
Simonse 2012[39]	Low	Low	High	High	Low	High	High	High
Singh 2017[31]	Unclear	Low	High	Unclear	High	High	High	High
Skogsdal 1997[46]	Low	Unclear	High	Low	Low	Low	Unclear	Low
Slater 2010[85]	Low	Low	Low	Low	Low	Low	Low	Low
Soliman 2016[172]	Low	Low	High	Low	Low	Low	Low	Low
Soltani 2018[37]	Unclear	Low	Low	Low	Low	Low	Low	Low
Stevens 1999[173]	Unclear	Unclear	Unclear	Unclear	Low	Low	High	High
Stevens 1999[174]	Low	High	High	Unclear	Low	Low	High	High
Stevens 2018[175]	Low	Low	High	Low	High	Low	High	High
Suhrabi 2014[86]	Unclear	Unclear	High	High	Low	Low	High	High
Sujatha 2017[176]	High	High	High	Low	High	High	High	High
Taddio 2008[87]	Low	Low	Low	Low	High	Low	High	Low
Taddio 2011[177]	Low	Low	Low	Low	Unclear	Unclear	Low	Low
Thakkar 2016[88]	Low	Low	High	High	Low	Low	Unclear	Low
Tutag Lehr 2015[89]	Low	Low	Low	Low	Low	Low	Low	Low
Upadhyay 2004[102]	Low	Low	Low	Low	Low	Low	Low	Low
Uyan 2005[103]	Unclear	Unclear	Unclear	Unclear	Low	Low	Low	High
Uzelli 2015[90]	Low	High	High	High	High	High	High	High
Yilmaz 2011[91]	Unclear	Unclear	High	Unclear	Low	Low	Unclear	High
Zargham-Boroujeni 2017[32]	Low	Unclear	High	High	High	High	Low	High
Zhu 2015[133]	Low	Unclear	Unclear	Unclear	Low	Low	Low	High

Legend for Cochrane risk of bias assessment

High	(red)
Low	(green)
Unclear	(yellow)

Supplementary Figure 1: Standardised mean differences and their confidence intervals for the comparison of breastfeeding versus placebo or no intervention for heart rate

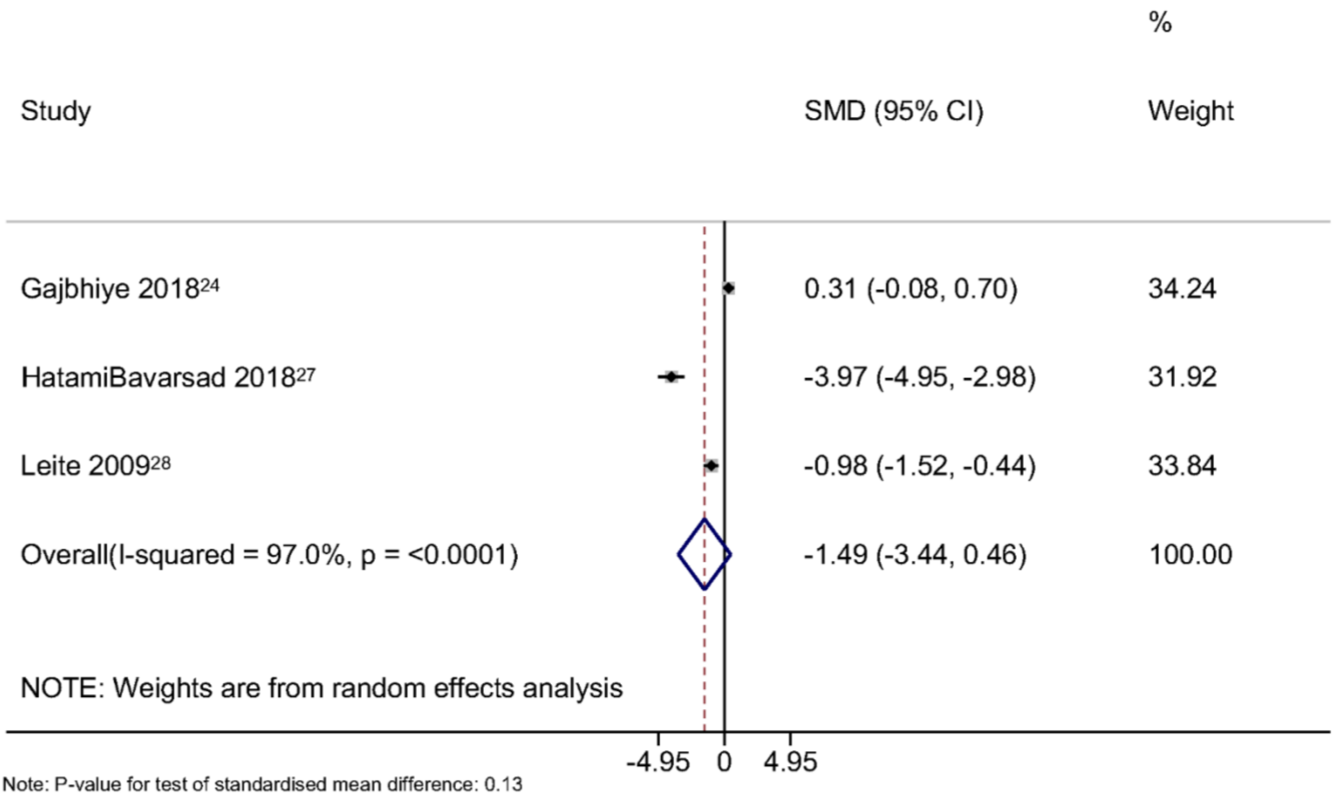

Note: P-value for test of standardised mean difference: 0.13

Abbreviations: CI=confidence interval, SMD=standardised mean difference

Supplementary Figure 2: Standardised mean differences and their confidence intervals for the comparison of breastfeeding versus placebo or no intervention for oxygen saturation

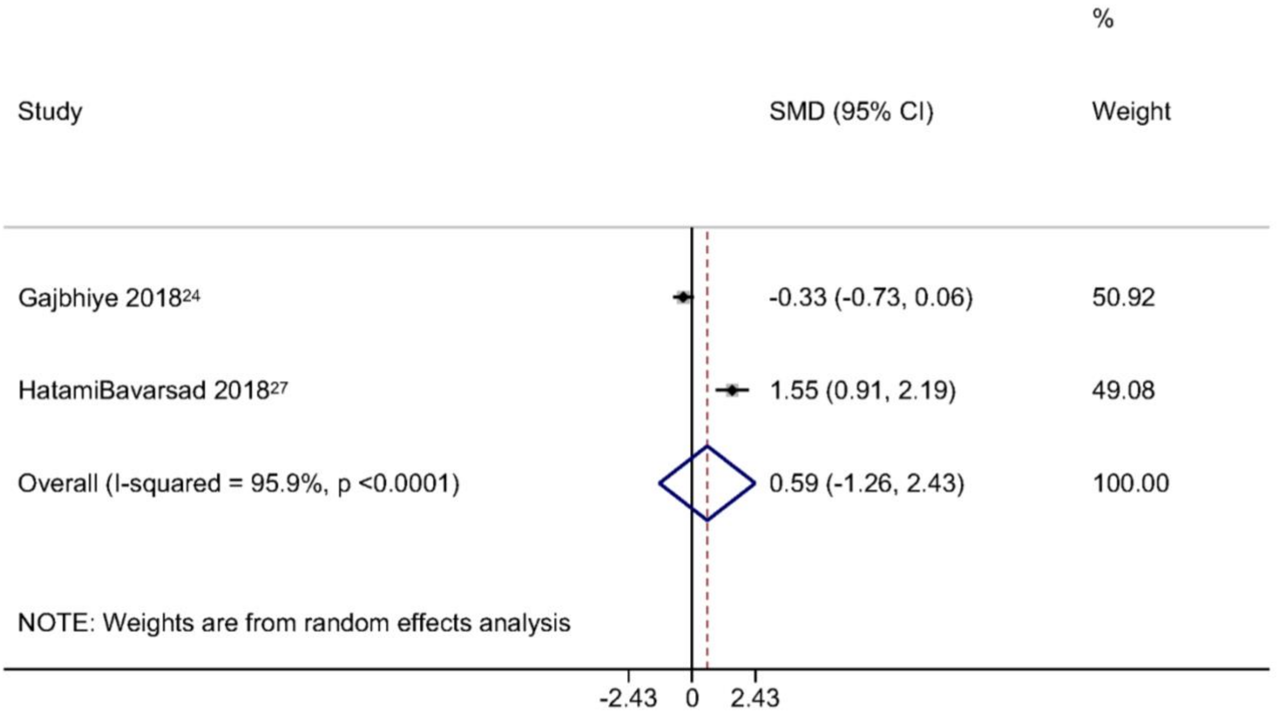

Note: P-value for test of standardised mean difference: 0.53
Abbreviations: CI=confidence interval, SMD=standardised mean difference

Supplementary Figure 3: Standardised mean differences and their confidence intervals for the comparison of breastfeeding versus placebo or no intervention for NIPS

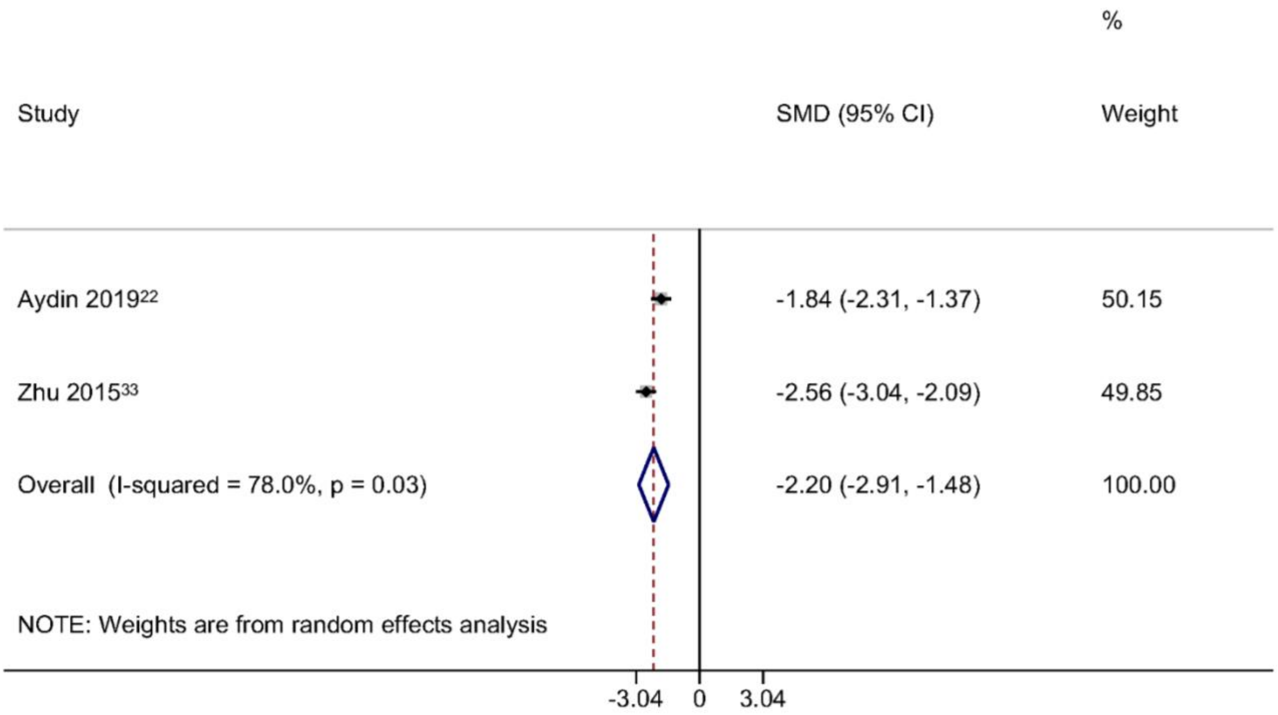

Note: P-value for test of standardised mean difference: < 0.001

Abbreviations: NIPS=Neonatal Infant Pain Scale. CI=confidence interval. SMD=standardised mean difference

Supplementary Figure 4: Standardised mean differences and their confidence intervals for the comparison of breastfeeding versus oral sugar for PIPP

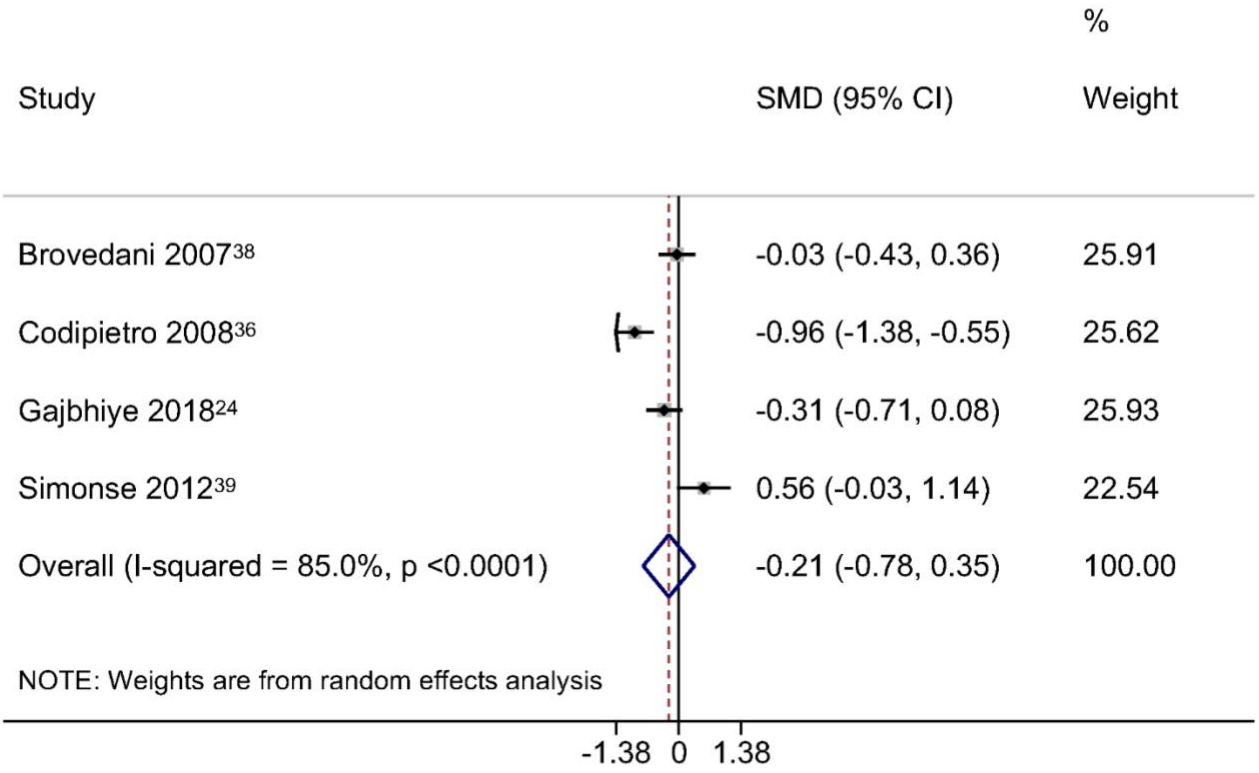

Note: P-value for test of standardised mean difference: 0.46

Abbreviations: CI=confidence interval. SMD=standardised mean difference. PIPP=Premature Infant Pain Profile

Supplementary Figure 5: Standardised mean differences and their confidence intervals for the comparison of breastfeeding versus oral sugar for NIPS

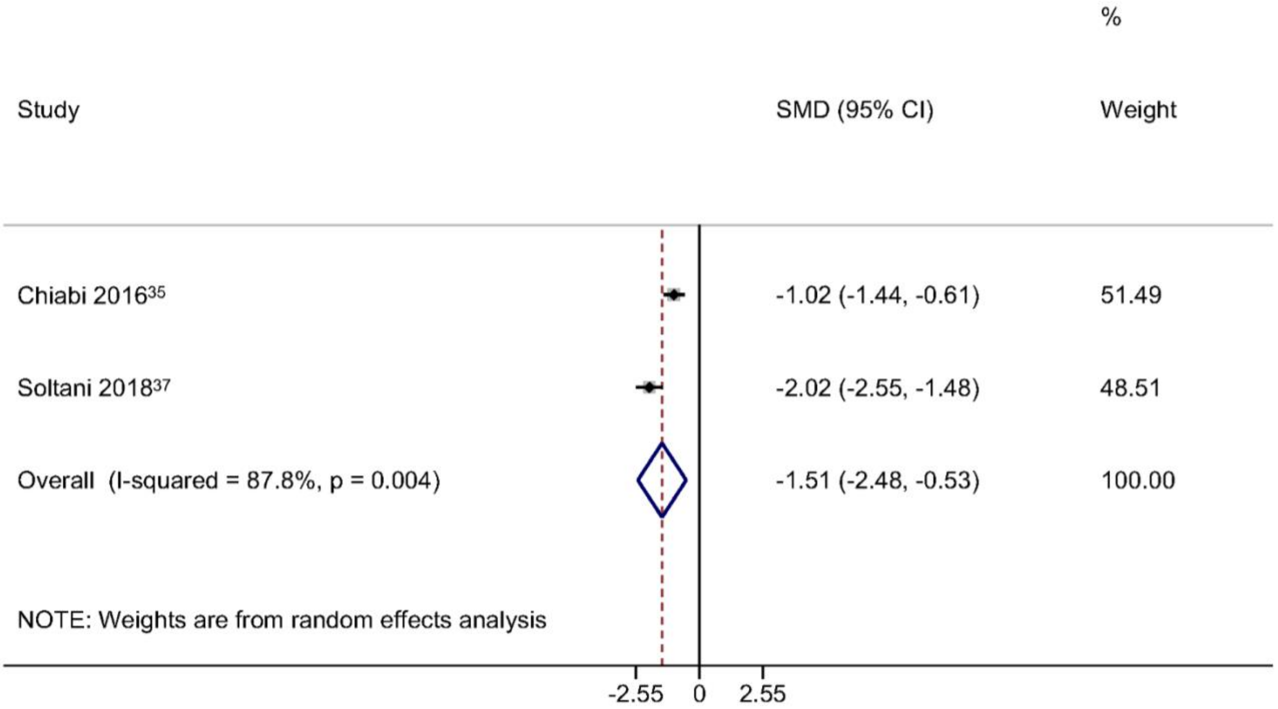

Note: P-value for test of standardised mean difference: 0.002

Abbreviations: CI=confidence interval, SMD=standardised mean difference, NIPS=Neonatal Infant Pain Scale

Supplementary Figure 6: Standardised mean differences and their confidence intervals for the comparison of breastfeeding versus skin-to-skin for NIPS

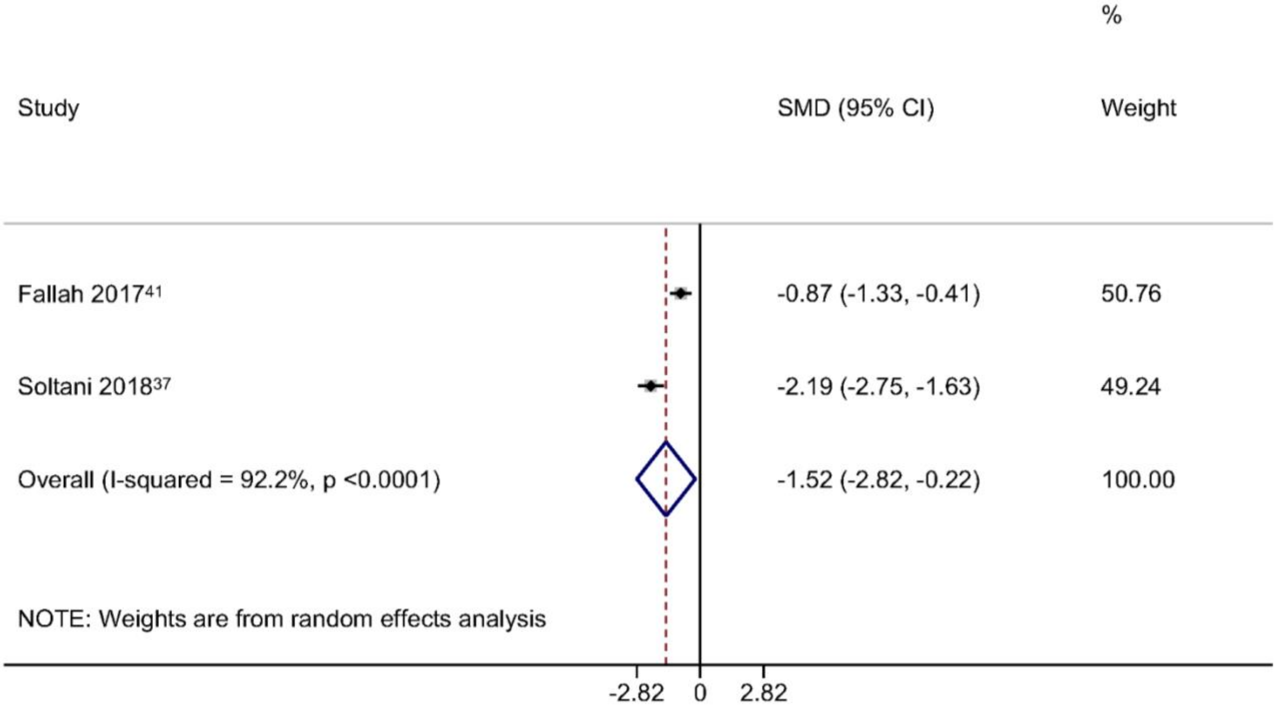

Note: P-value for test of standardised mean difference: 0.02

Abbreviations: CI=confidence interval. SMD=standardised mean difference. NIPS=Neonatal Infant Pain Scale

Supplementary Figure 7. Standardised mean differences and their confidence intervals for the comparison of oral sugar versus placebo or no intervention for heart rate

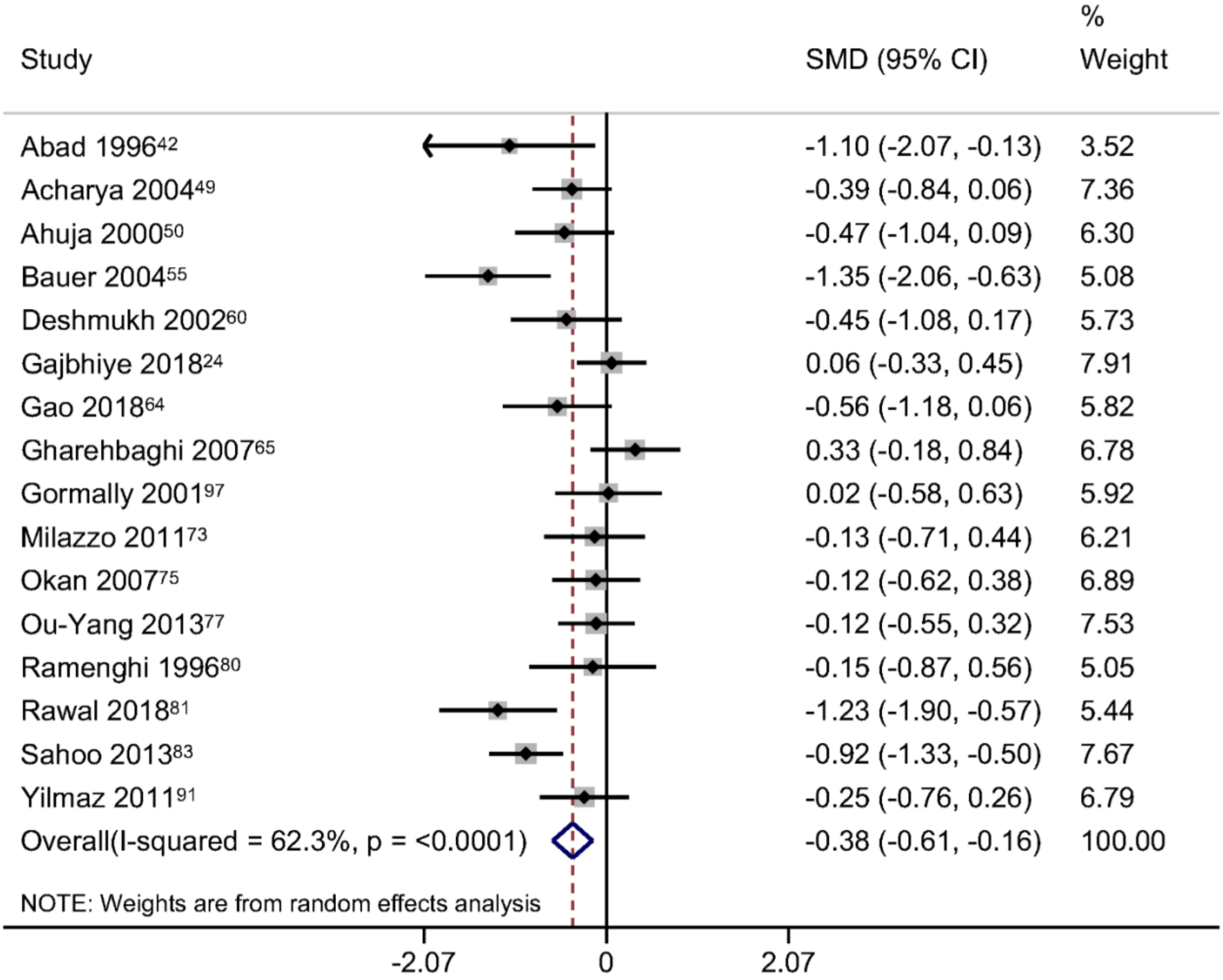

Note: P-value for test of standardised mean difference: 0.001
Abbreviations: CI=confidence interval, SMD=standardised mean difference

Supplementary Figure 8. Standardised mean differences and their confidence intervals for the comparison of oral sugar versus placebo or no intervention for PIPP

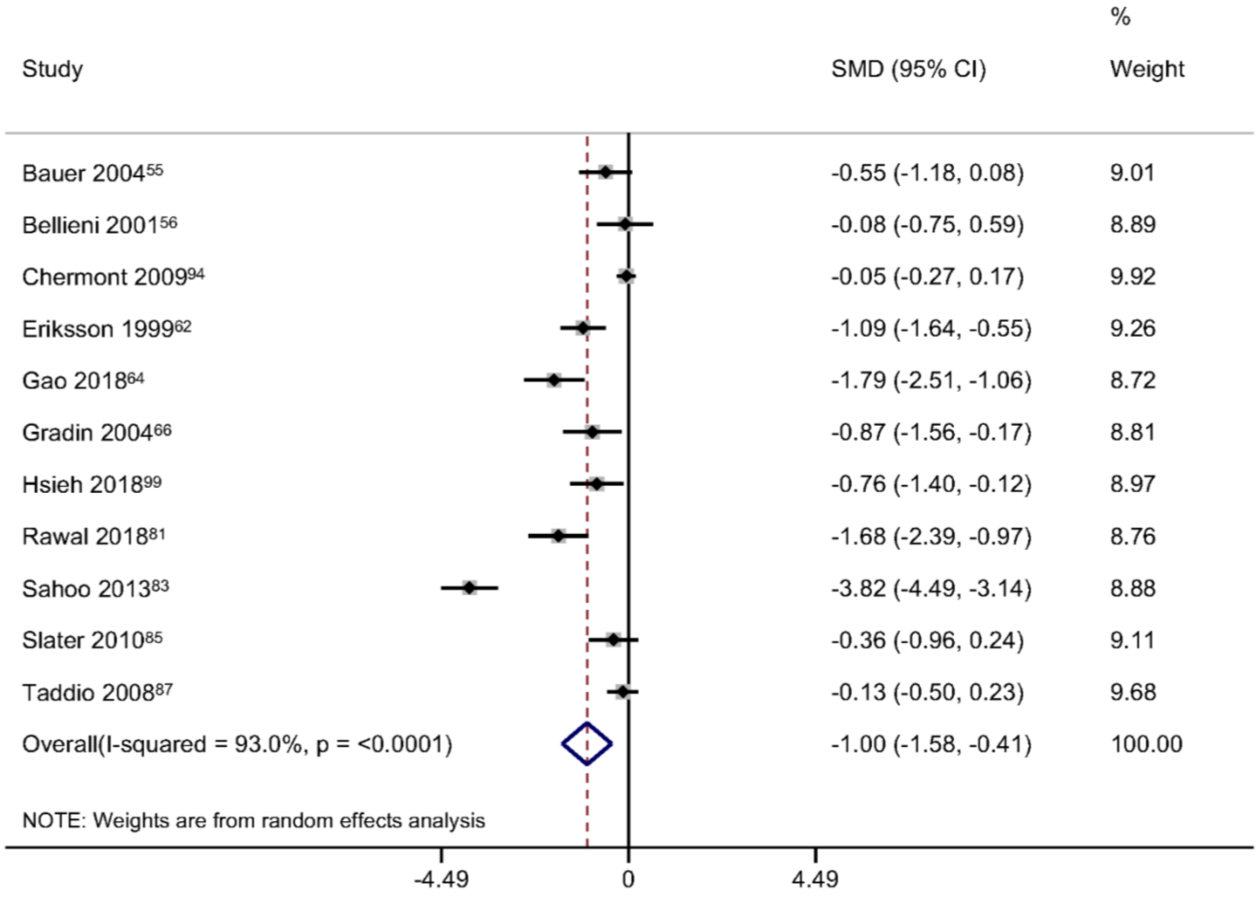

Note: P-value for test of standardised mean difference: 0.001

Abbreviations: CI=confidence interval, SMD=standardised mean difference, PIPP=Premature Infant Pain Profile

Supplementary Figure 9. Standardised mean differences and their confidence intervals for the comparison of oral sugar versus placebo or no intervention for NIPS

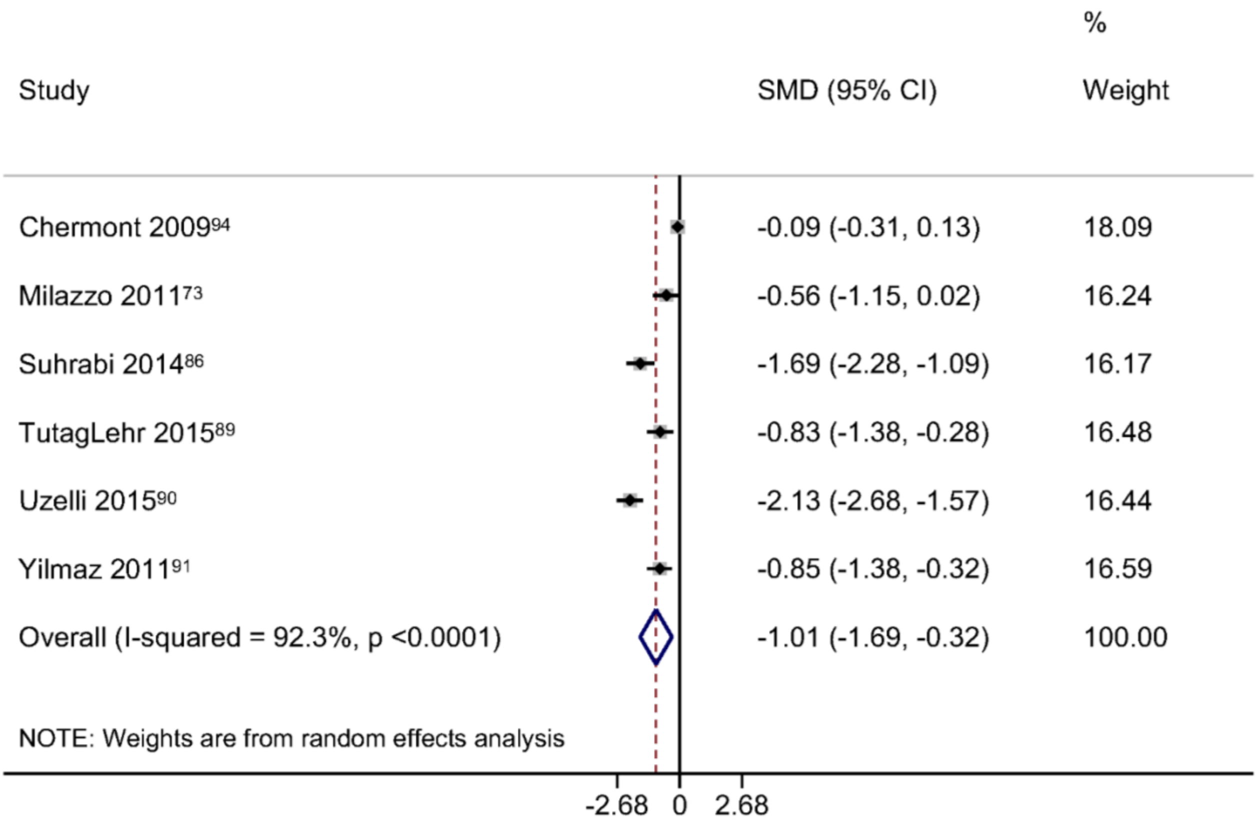

Note: P-value for test of standardised mean difference: 0.004

Abbreviations: CI=confidence interval, SMD=standardised mean difference, NIPS=Neonatal Infant Pain Scale

Supplementary Figure 10. Standardised mean differences and their confidence intervals for the comparison of oral sugar versus placebo or no intervention for NFCS

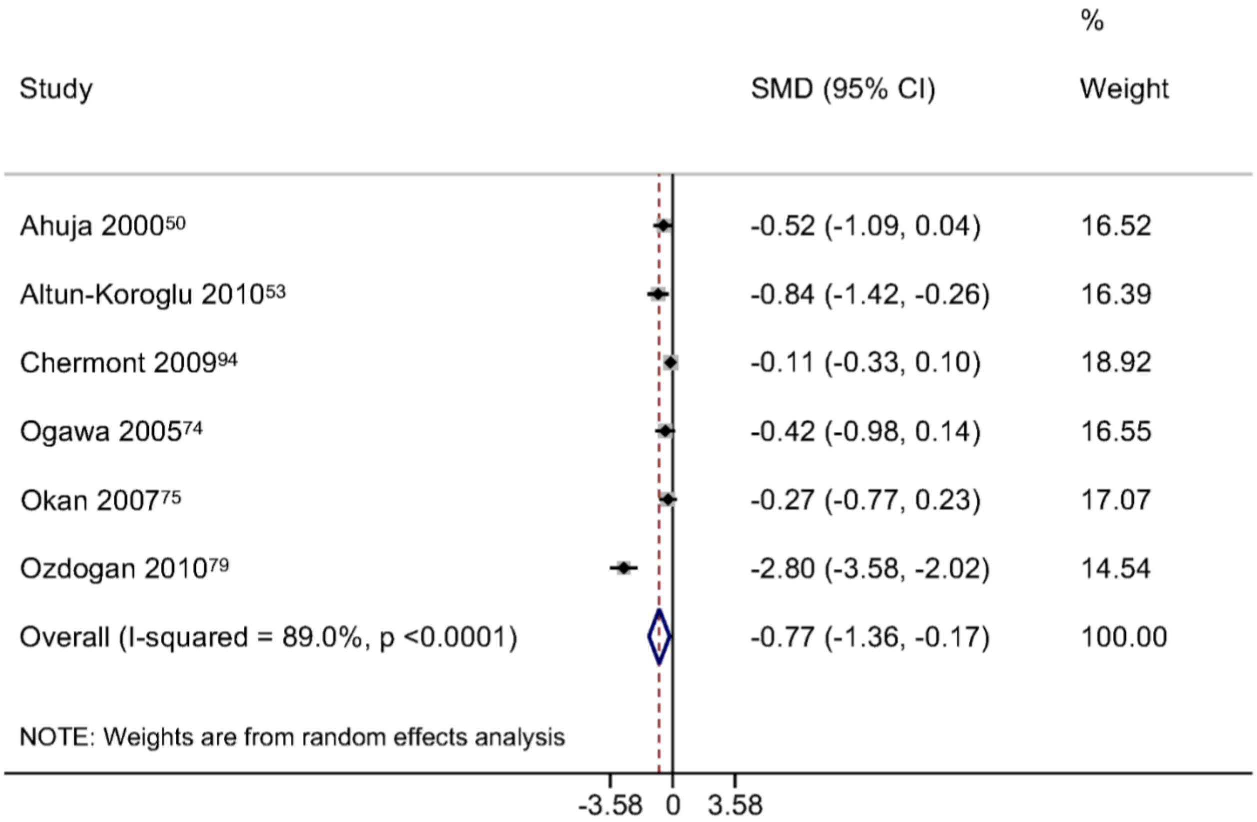

Note: P-value for test of standardised mean difference: 0.01

Abbreviations: CI=confidence interval, SMD=standardised mean difference, NCFS= Neonatal Facial Coding System

Supplementary Figure 11. Standardised mean differences and their confidence intervals for the comparison of oral sugar versus placebo or no intervention for DAN

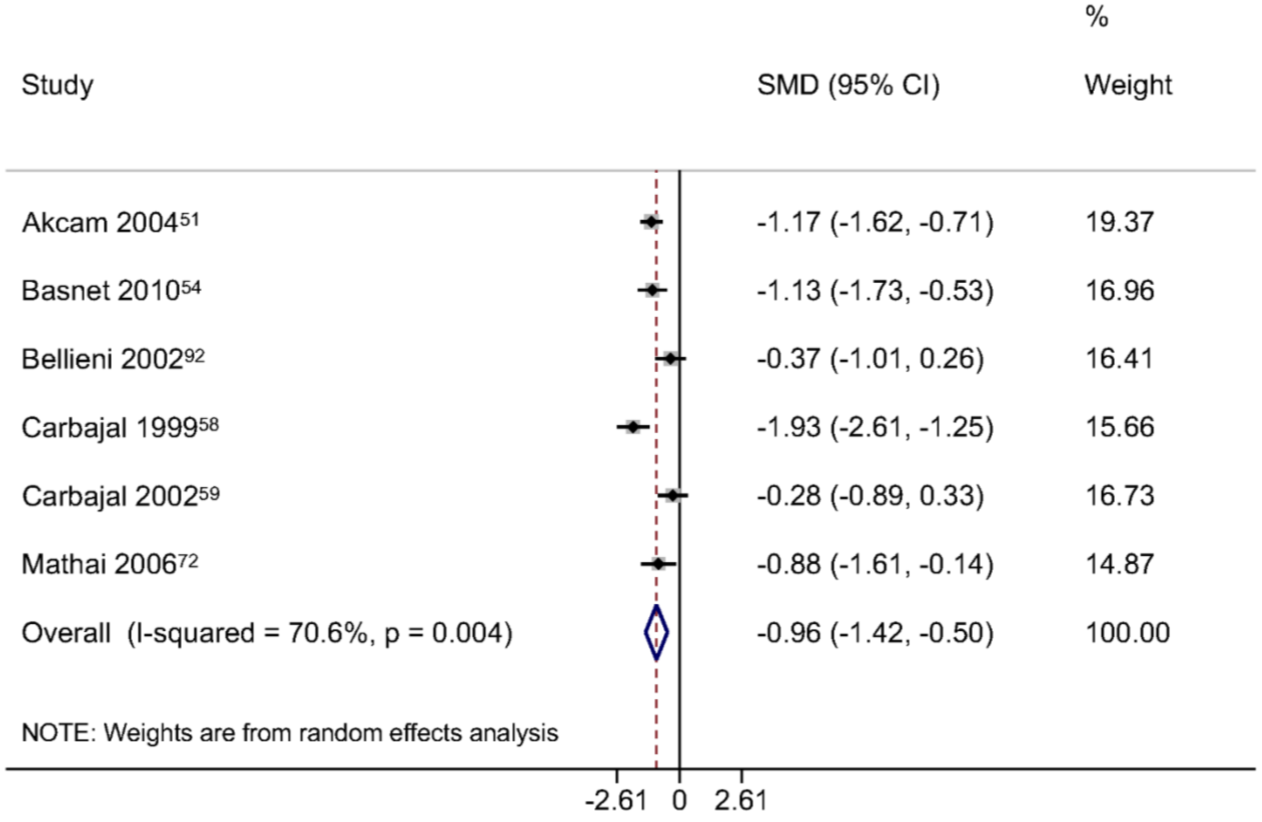

Note: P-value for test of standardised mean difference: p <0.001

Abbreviations: CI=confidence interval, SMD=standardised mean difference, DAN= Douleur Aigue du Nouveau-né

Supplementary Figure 12. Standardised mean differences and their confidence intervals for the comparison of expressed breast milk versus oral sugar for heart rate

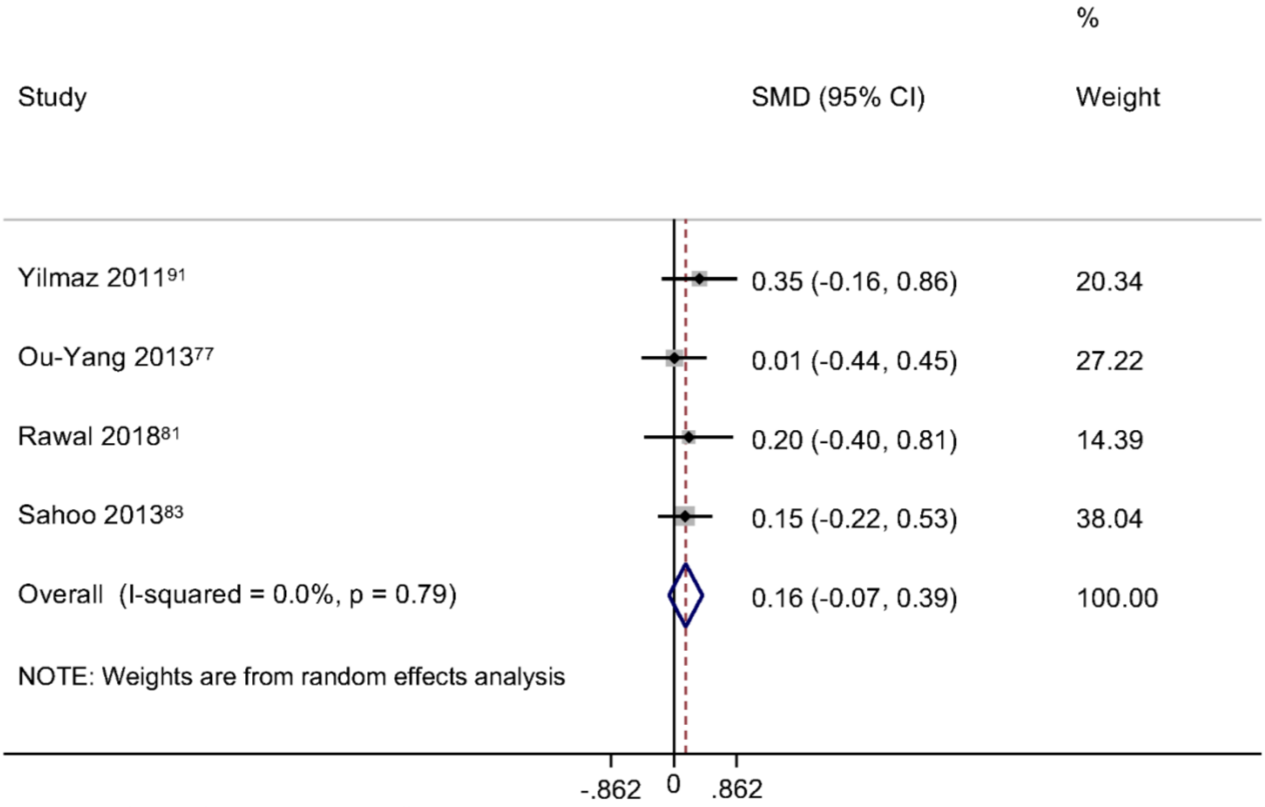

Note: P-value for test of standardised mean difference: 0.17

Abbreviations: CI=confidence interval, SMD=standardised mean difference

Supplementary Figure 13. Standardised mean differences and their confidence intervals for the comparison of expressed breast milk vs oral sugar for oxygen saturation

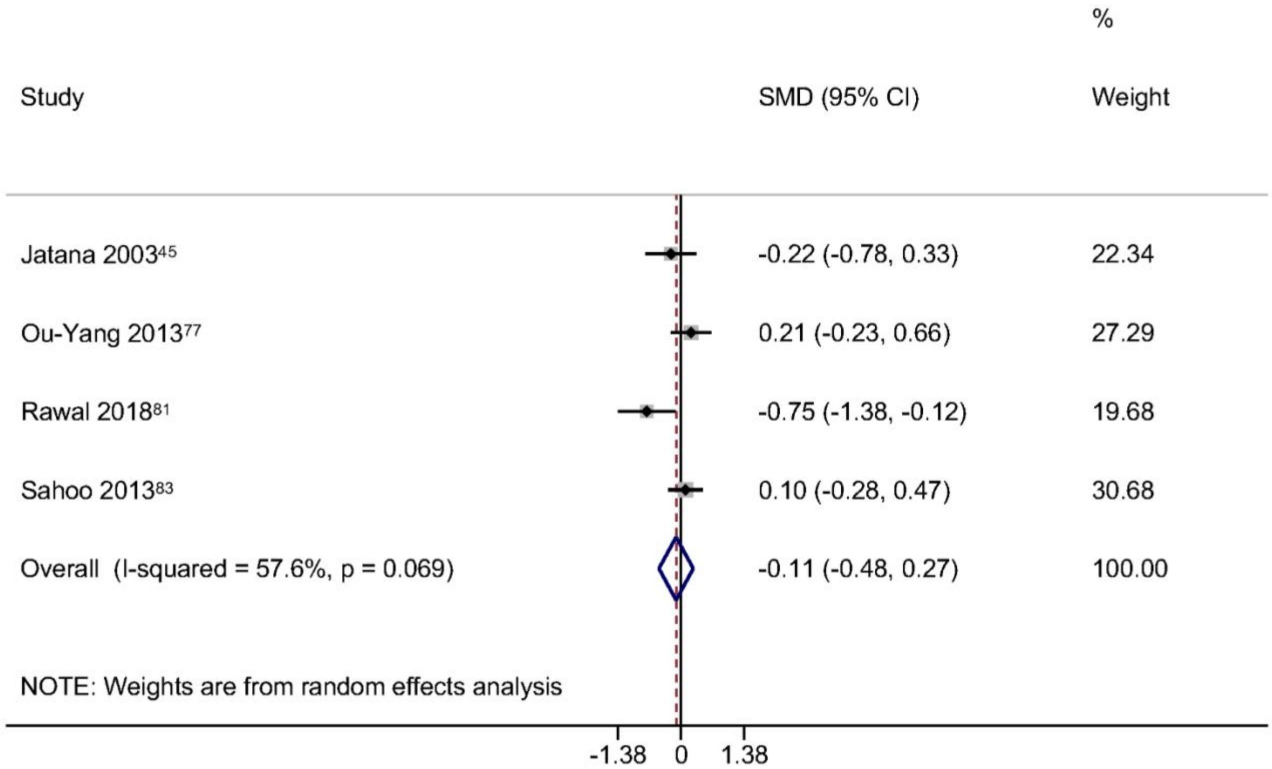

Note: P-value for test of standardised mean difference: 0.57

Abbreviations: CI=confidence interval, SMD=standardised mean difference

Supplementary Figure 14. Standardised mean differences and their confidence intervals for the comparison of expressed breast milk versus oral sugar for PIPP

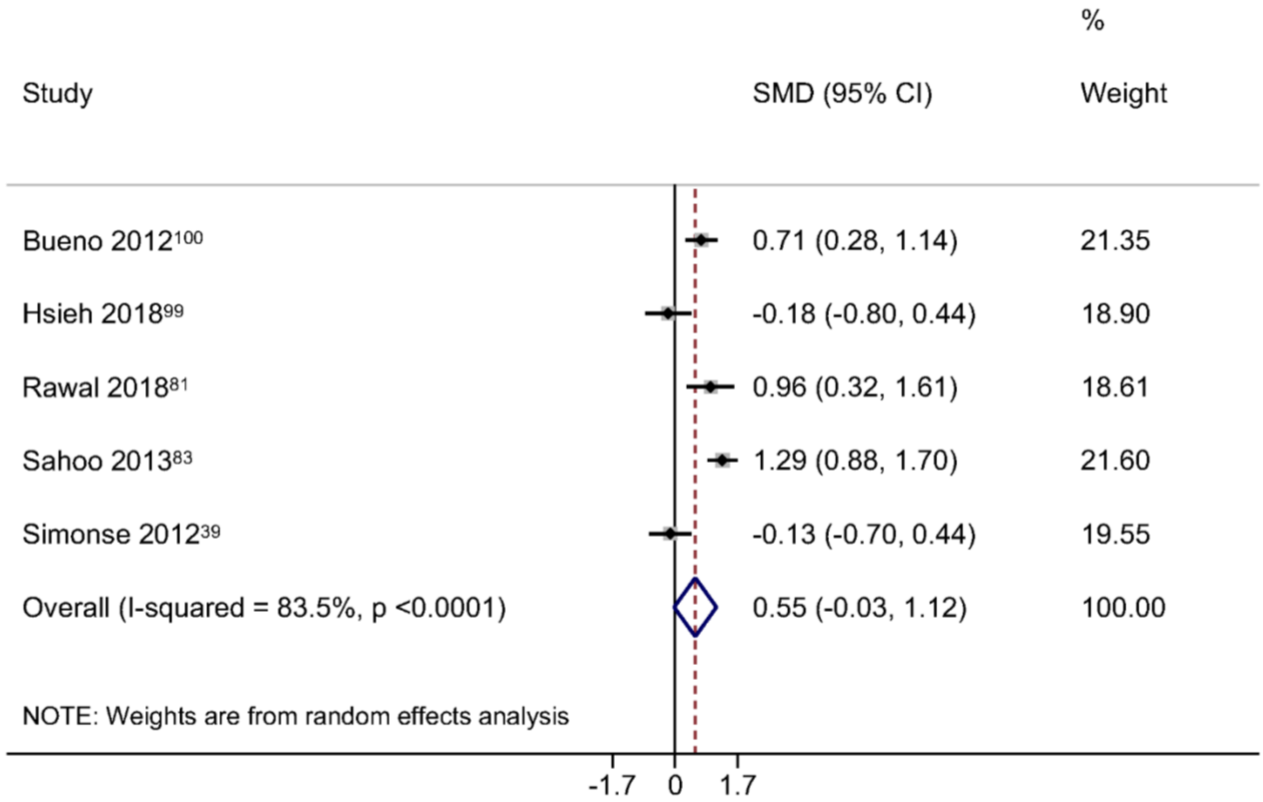

Note: P-value for test of standardised mean difference: 0.061

Abbreviations: CI=confidence interval, SMD=standardised mean difference, PIPP=Premature Infant Pain Profile

Supplementary Figure 15. Standardised mean differences and their confidence intervals for the comparison of expressed breast milk versus oral sugar for NFCS

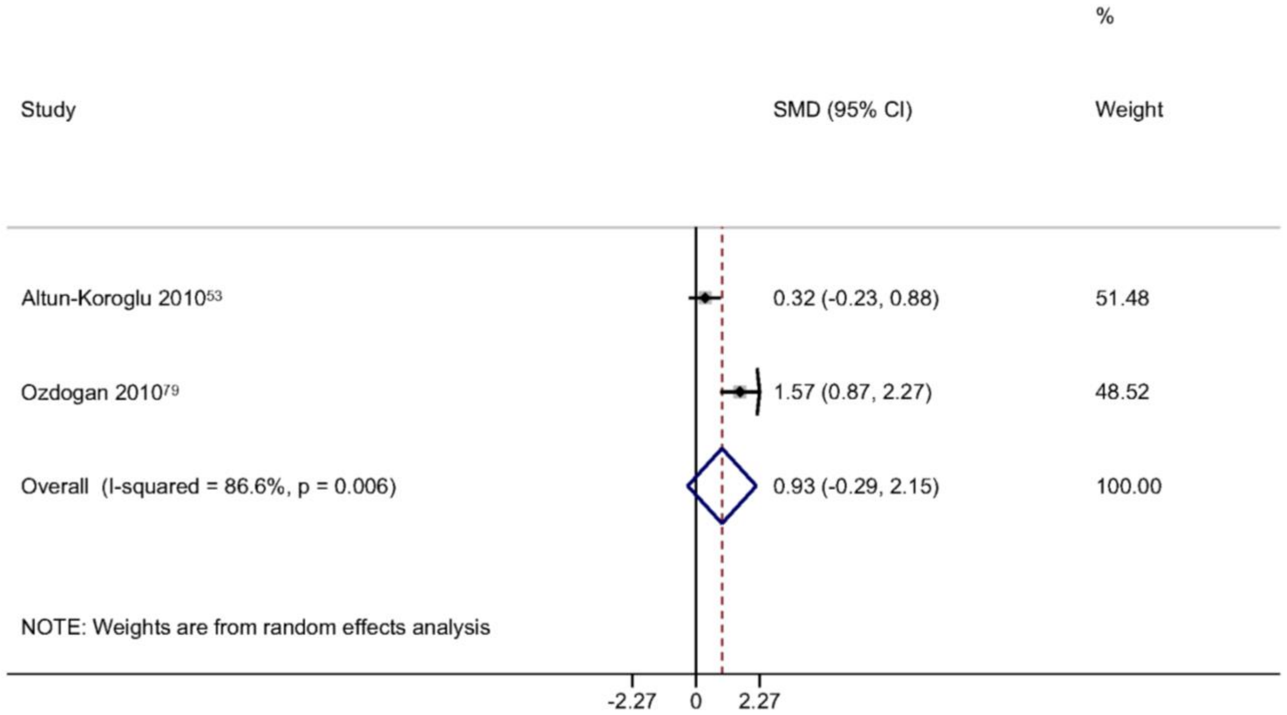

Note: P-value for test of standardised mean difference: 0.14

Abbreviations: CI=confidence interval, SMD=standardised mean difference, NCFS= Neonatal Facial Coding System

Supplementary Figure 16. Standardised mean differences and their confidence intervals for the comparison of non-nutritive sucking versus oral sugar for heart rate

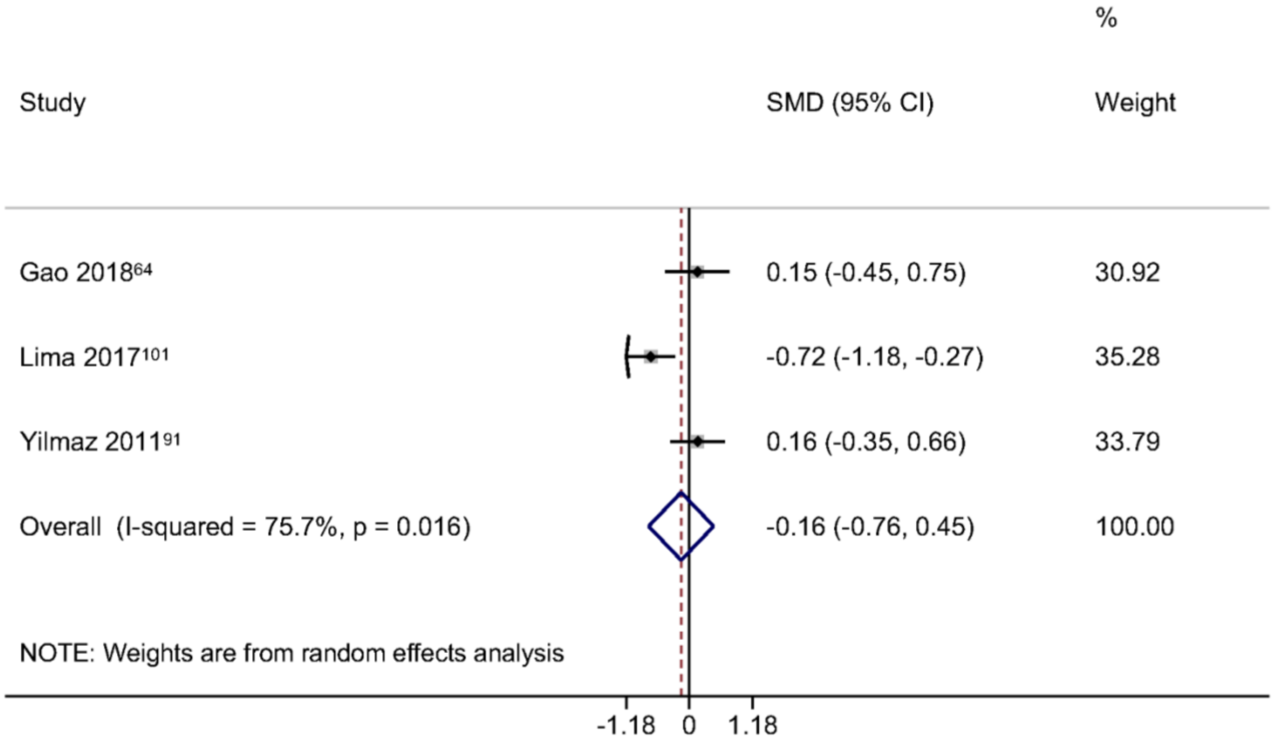

Note: P-value for test of standardised mean difference: 0.62
Abbreviations: CI=confidence interval, SMD=standardised mean difference

Supplementary Figure 17. Standardised mean differences and their confidence intervals for the comparison of non-nutritive sucking vs oral sugar for oxygen saturation

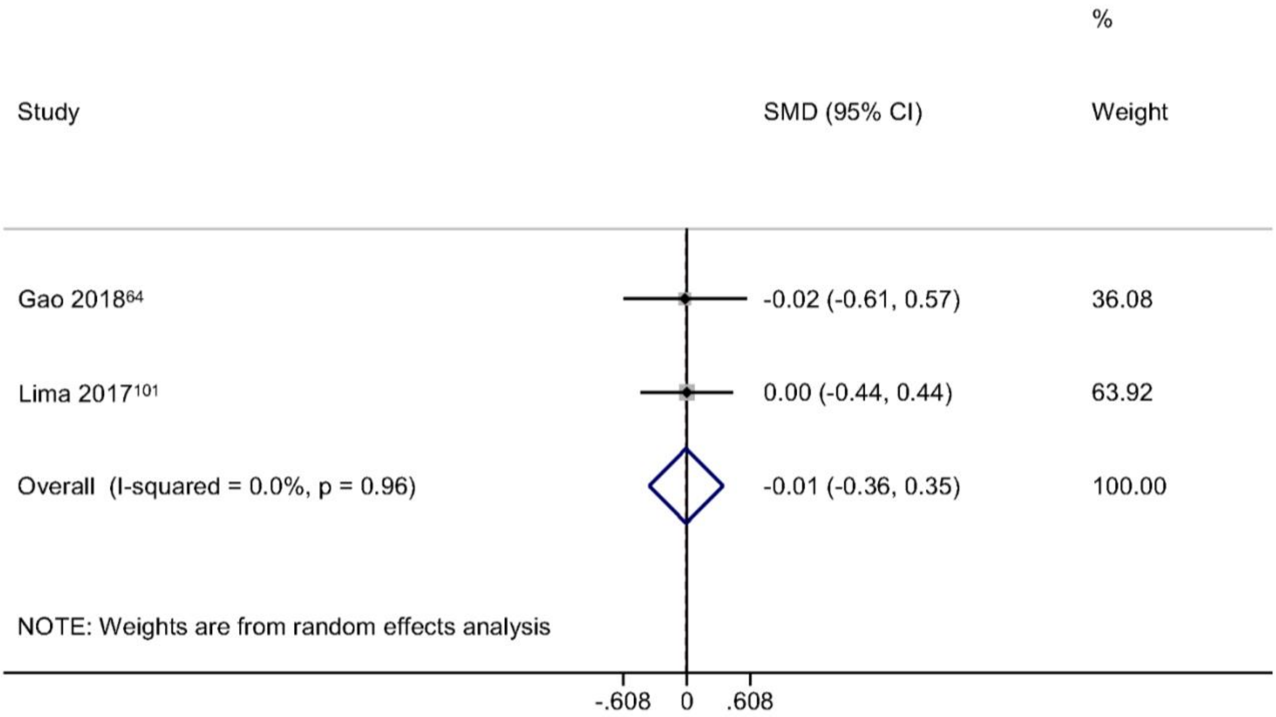

Note: P-value for test of standardised mean difference: 0.97

Abbreviations: CI=confidence interval, SMD=standardised mean difference

Supplementary Figure 18. Standardised mean differences and their confidence intervals for the comparison of non-nutritive sucking versus oral sugar for NIPS

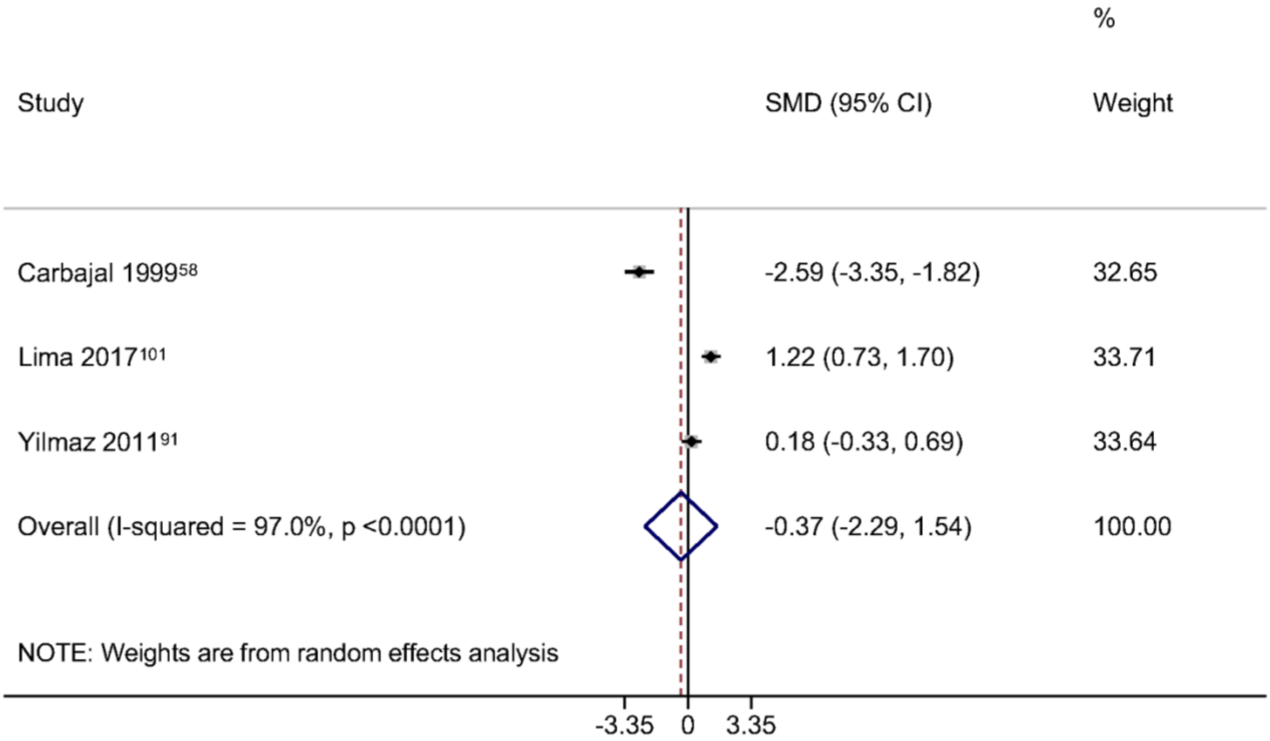

Note: P-value for test of standardised mean difference: 0.70

Abbreviations: CI=confidence interval, SMD=standardised mean difference, NIPS=Neonatal Infant Pain Scale

Supplementary Figure 19. Standardised mean differences and their confidence intervals for the comparison of non-nutritive sucking versus oral sugar for DAN

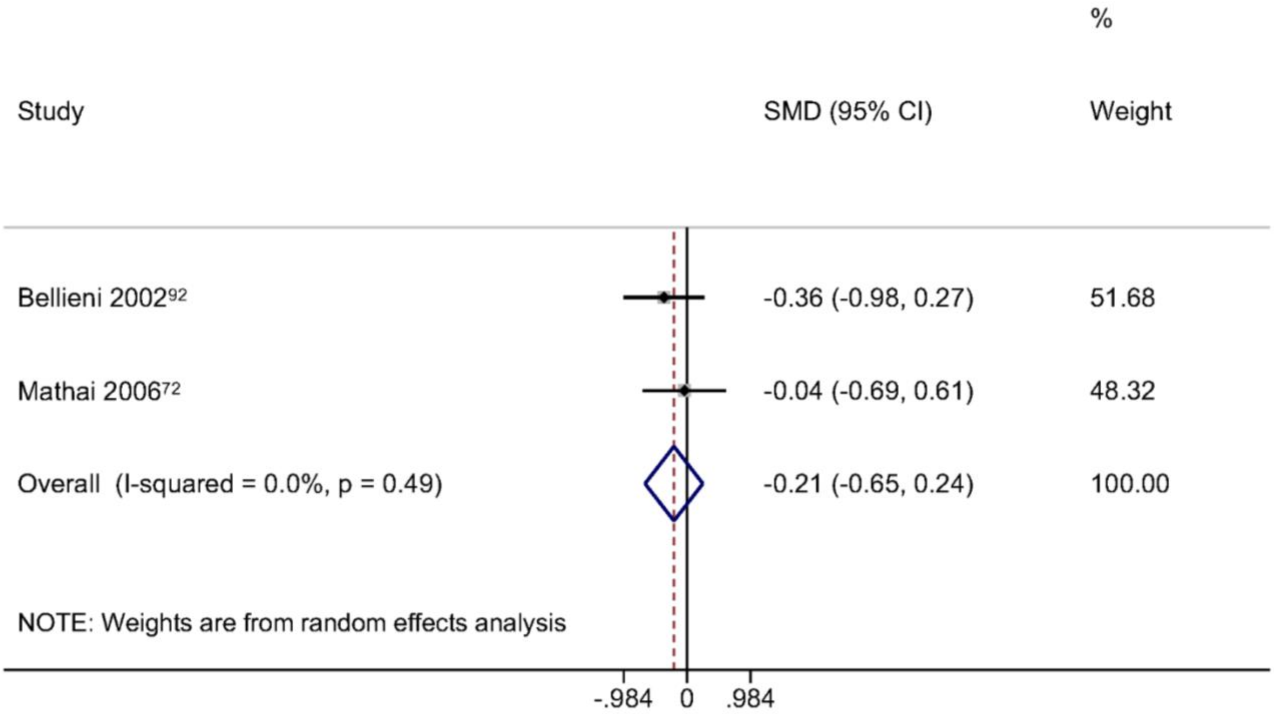

Note: P-value for test of standardised mean difference: 0.37

Abbreviations: CI=confidence interval, SMD=standardised mean difference, DAN= Douleur Aigue du Nouveau-né

Supplementary Figure 20. Standardised mean differences and their confidence intervals for the comparison of expressed breast milk versus placebo or no intervention for heart rate

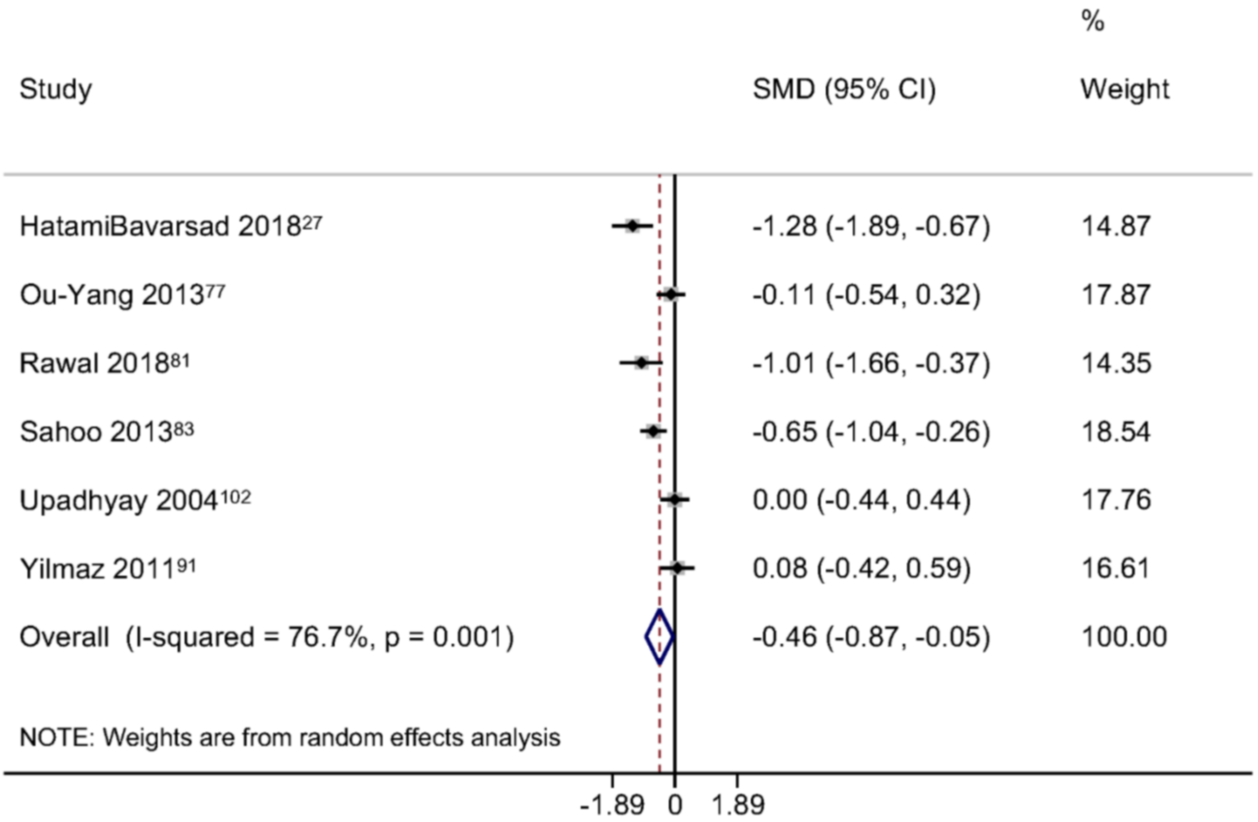

Note: P-value for test of standardised mean difference: 0.028

Abbreviations: CI=confidence interval, SMD=standardised mean difference

Supplementary Figure 21. Standardised mean differences and their confidence intervals for the comparison of expressed breast milk vs placebo or no intervention for oxygen saturation

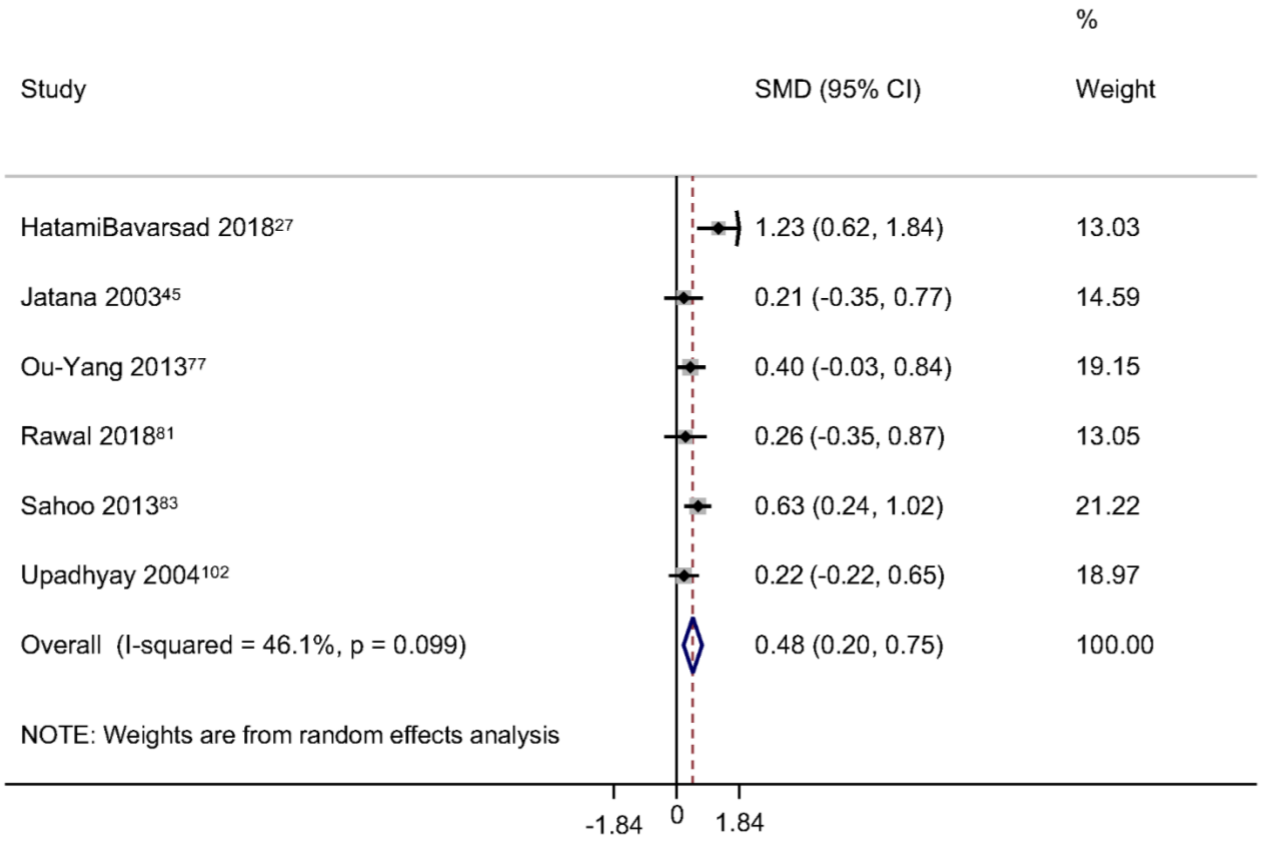

Note: P-value for test of standardised mean difference: <0.001

Abbreviations: CI=confidence interval, SMD=standardised mean difference

Supplementary Figure 22. Standardised mean differences and their confidence intervals for the comparison of expressed breast milk versus placebo or no intervention for PIPP

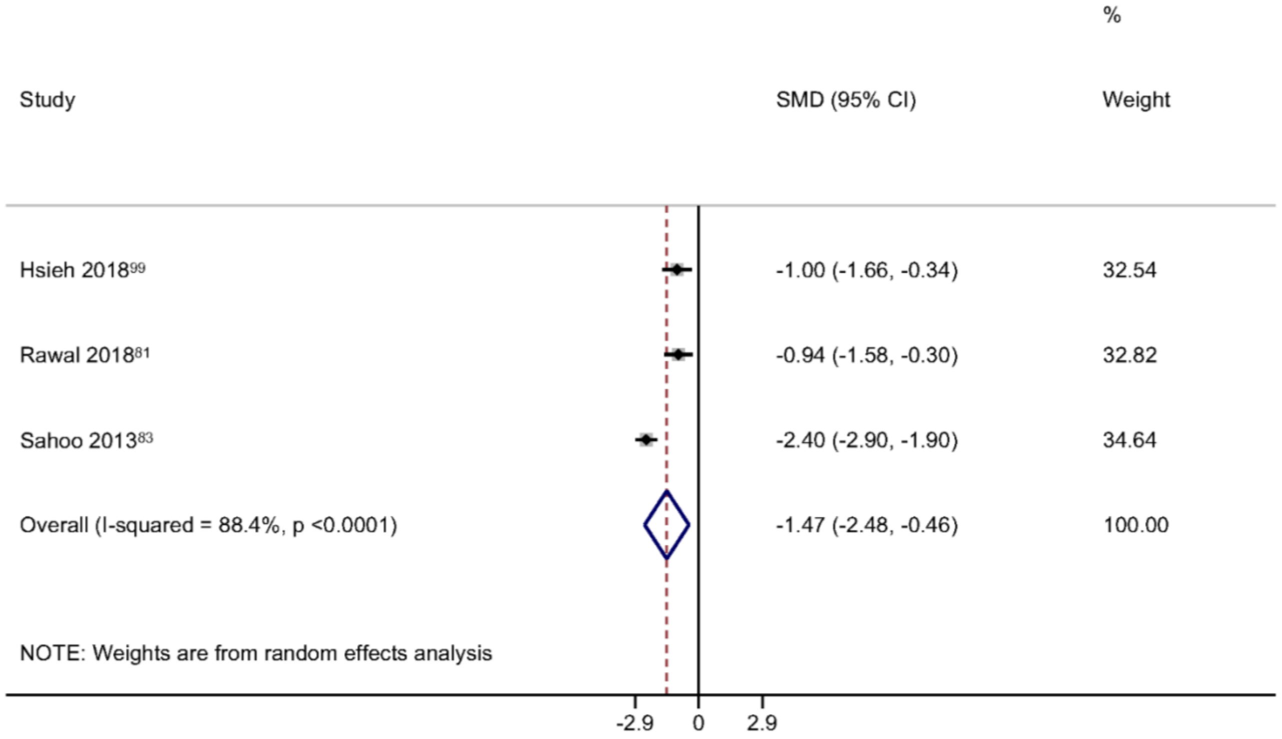

Note: P-value for test of standardised mean difference: 0.004

Abbreviations: CI=confidence interval, SMD=standardised mean difference, PIPP=Premature Infant Pain Profile

Supplementary Figure 23. Standardised mean differences and their confidence intervals for the comparison of expressed breast milk versus placebo or no intervention for DAN

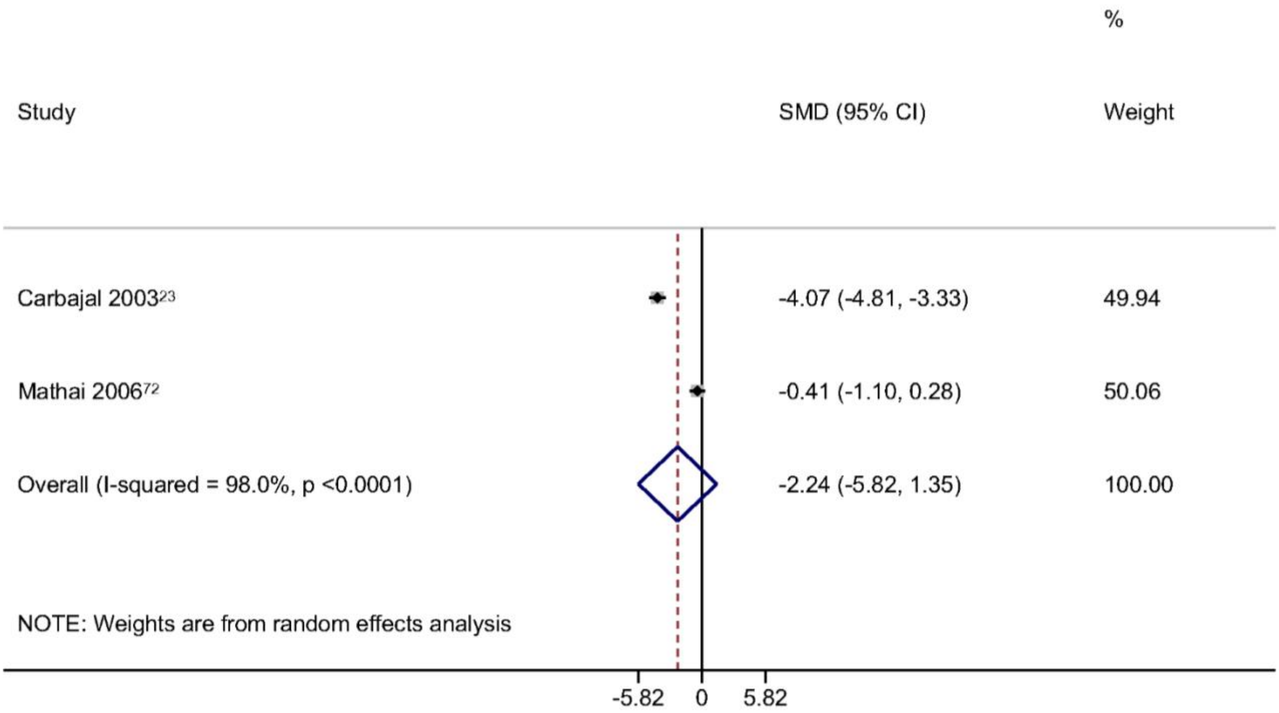

Note: P-value for test of standardised mean difference: 0.21

Abbreviations: CI=confidence interval, SMD=standardised mean difference, DAN= Douleur Aigue du Nouveau-né

Supplementary Figure 24. Standardised mean differences and their confidence intervals for the comparison of skin-to-skin versus placebo or no intervention for oxygen saturation

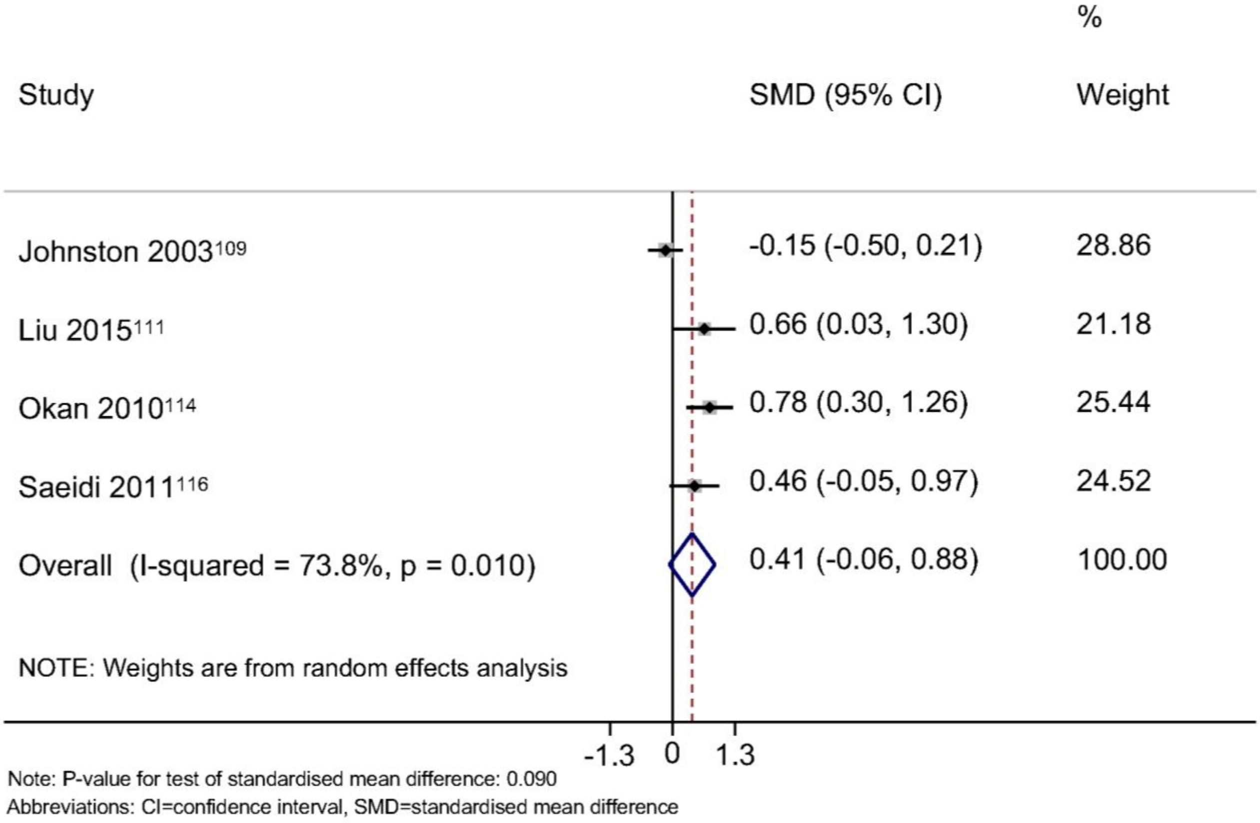

Supplementary Figure 25. Standardised mean differences and their confidence intervals for the comparison of skin-to-skin versus placebo or no intervention for NFCS

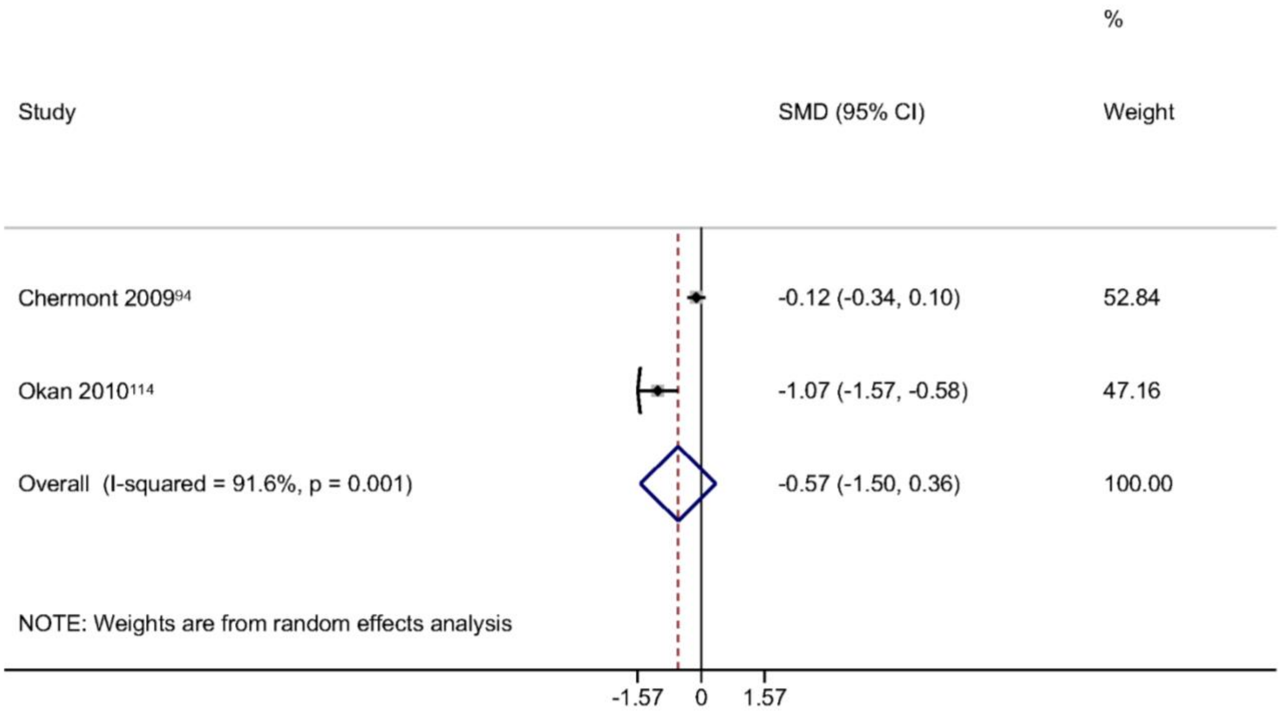

Note: P-value for test of standardised mean difference: 0.23

Abbreviations: CI=confidence interval, SMD=standardised mean difference, NCFS= Neonatal Facial Coding System

Supplementary Figure 26. Standardised mean differences and their confidence intervals for the comparison of non-nutritive sucking versus placebo or no intervention for heart rate

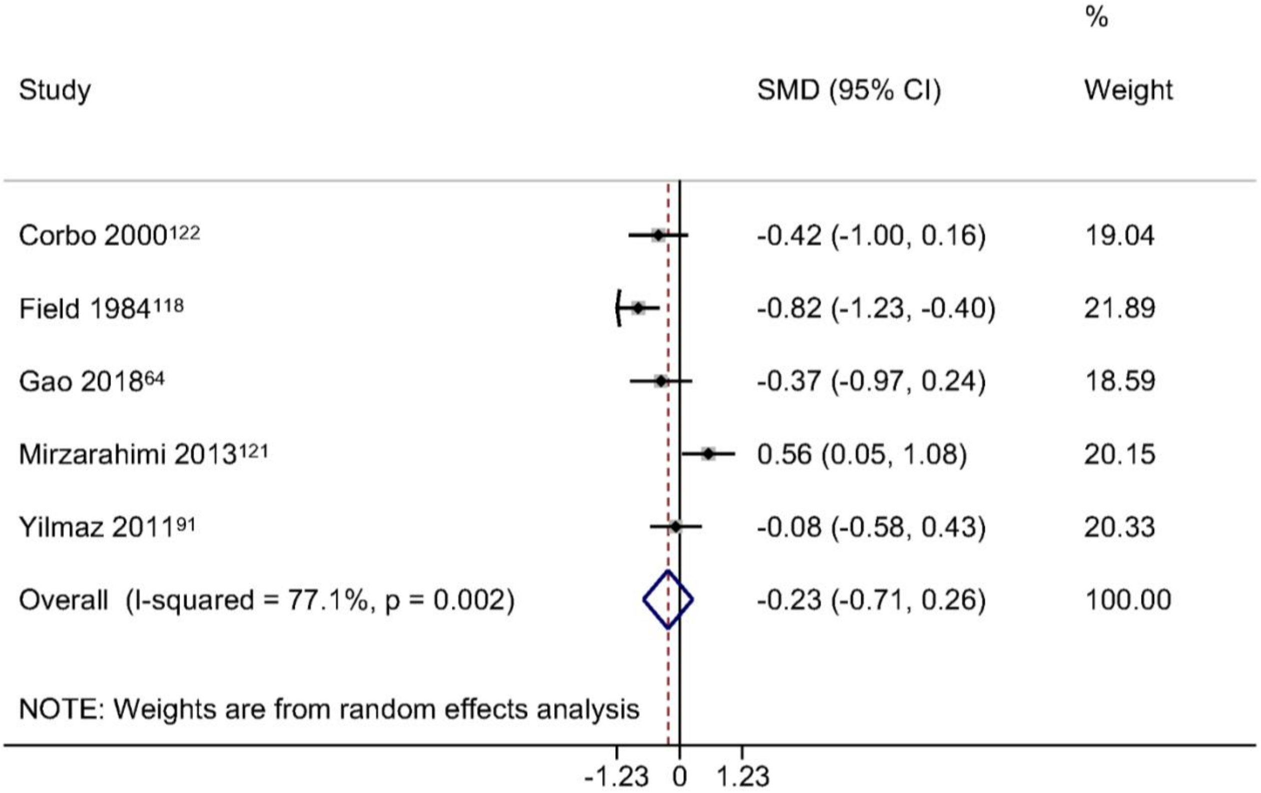

Note: P-value for test of standardised mean difference: 0.35

Abbreviations: CI=confidence interval, SMD=standardised mean difference

Supplementary Figure 27. Standardised mean differences and their confidence intervals for the comparison of non-nutritive sucking versus placebo or no intervention for oxygen saturation

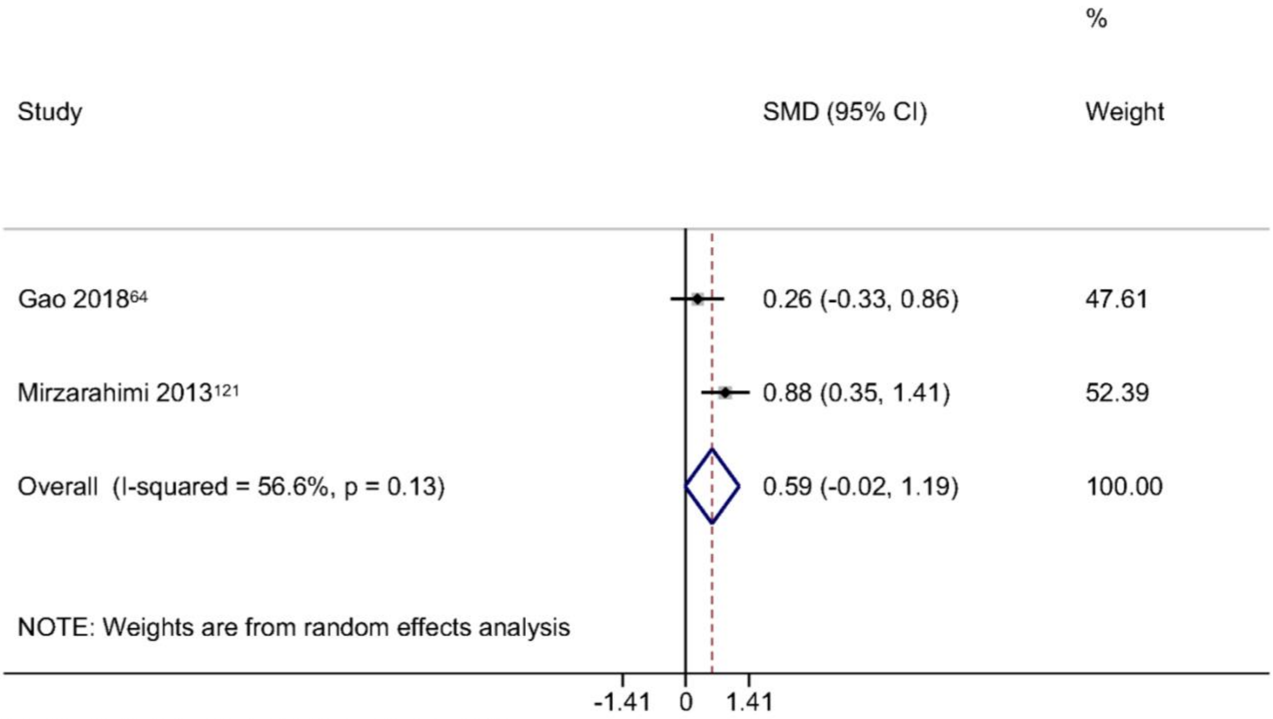

Note: P-value for test of standardised mean difference: 0.057

Abbreviations: CI=confidence interval, SMD=standardised mean difference

Supplementary Figure 28. Standardised mean differences and their confidence intervals for the comparison of non-nutritive sucking versus placebo or no intervention for PIPP

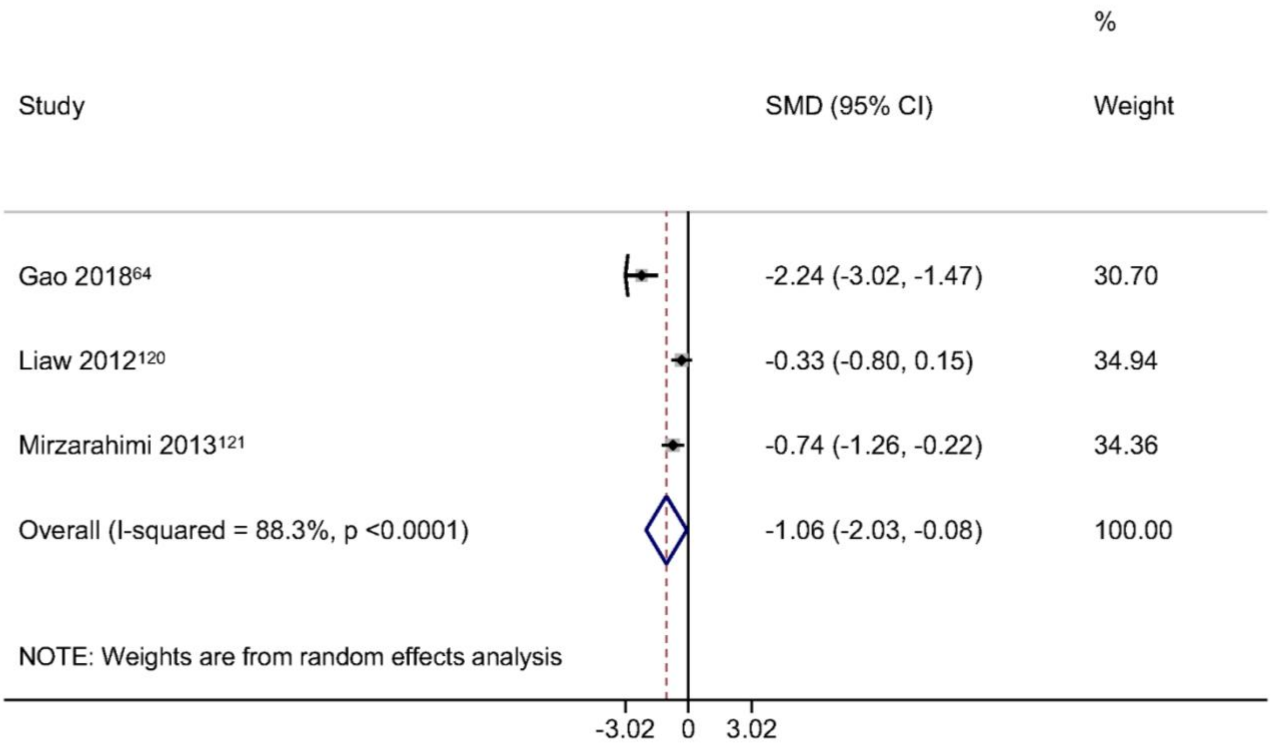

Note: P-value for test of standardised mean difference: 0.034

Abbreviations: CI=confidence interval, SMD=standardised mean difference, PIPP=Premature Infant Pain Profile

Supplementary Figure 29. Standardised mean differences and their confidence intervals for the comparison of non-nutritive sucking versus placebo or no intervention for DAN

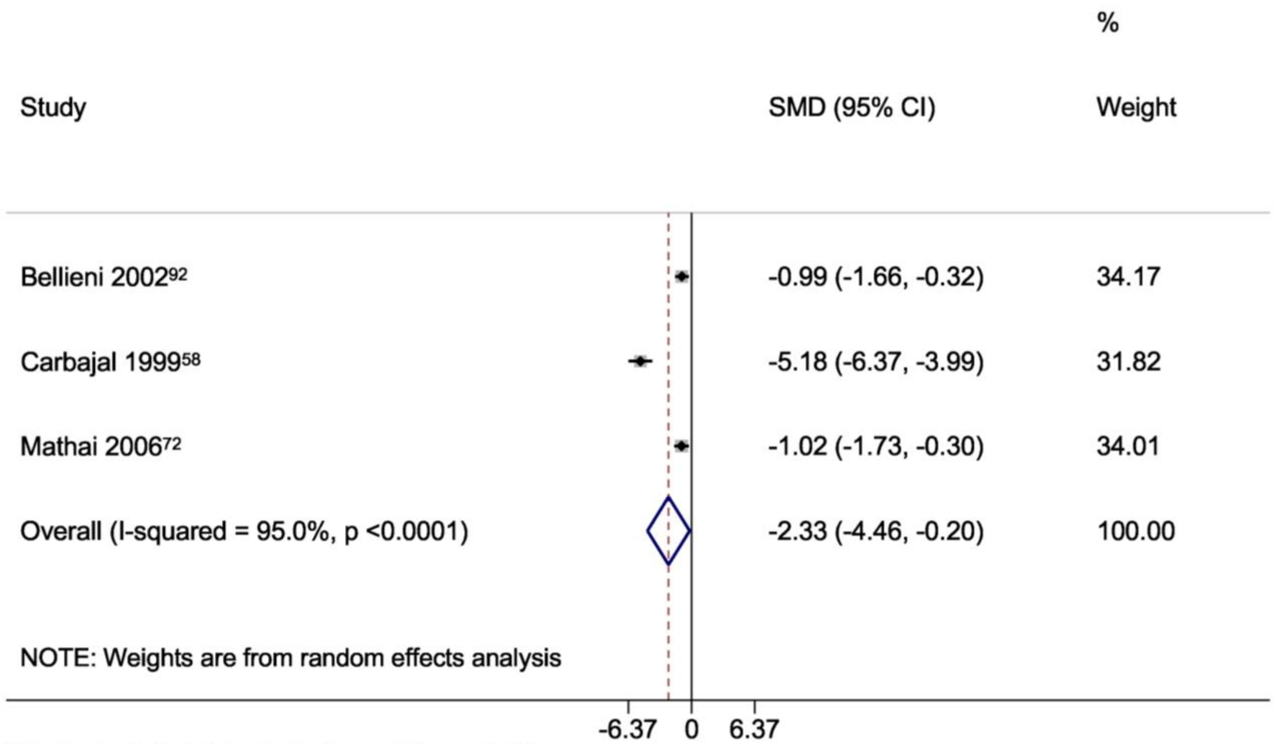

Note: P-value for test of standardised mean difference: 0.032

Abbreviations: CI=confidence interval, SMD=standardised mean difference, DAN= Douleur Aigue du Nouveau-né

Supplementary Figure 30. Standardised mean differences and their confidence intervals for the comparison of oral dextrose versus placebo or no intervention for heart rate

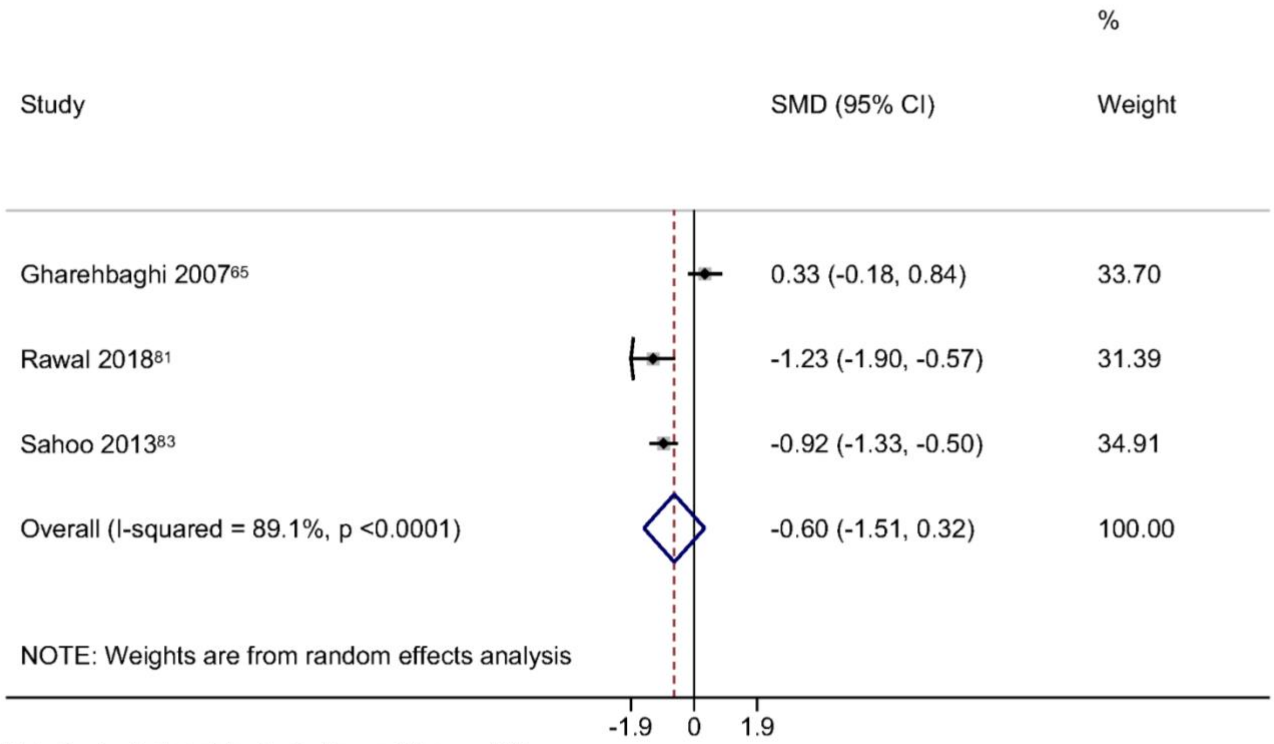

Note: P-value for test of standardised mean difference: 0.20

Abbreviations: CI=confidence interval, SMD=standardised mean difference

Supplementary Figure 31. Standardised mean differences and their confidence intervals for the comparison of oral glucose versus placebo or no intervention for heart rate

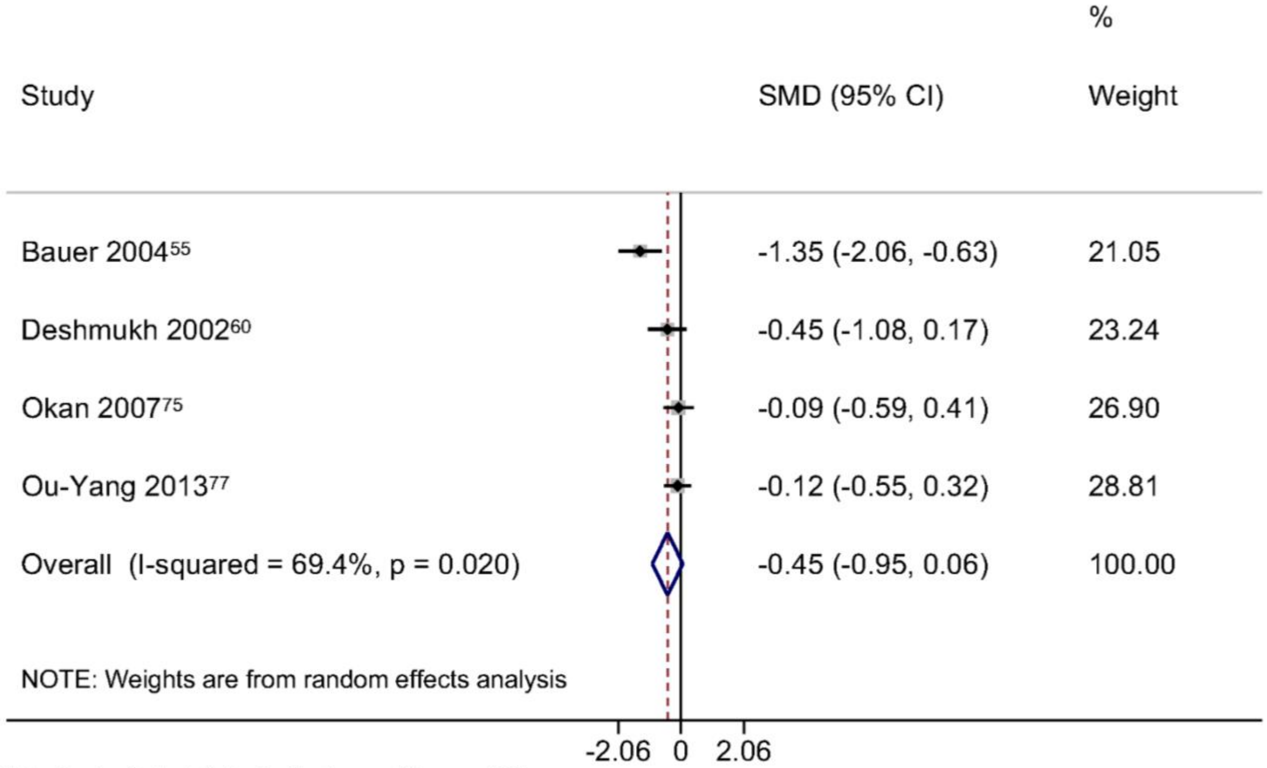

Note: P-value for test of standardised mean difference: 0.08

Abbreviations: CI=confidence interval, SMD=standardised mean difference

Supplementary Figure 32. Standardised mean differences and their confidence intervals for the comparison of oral sucrose versus placebo or no intervention for heart rate

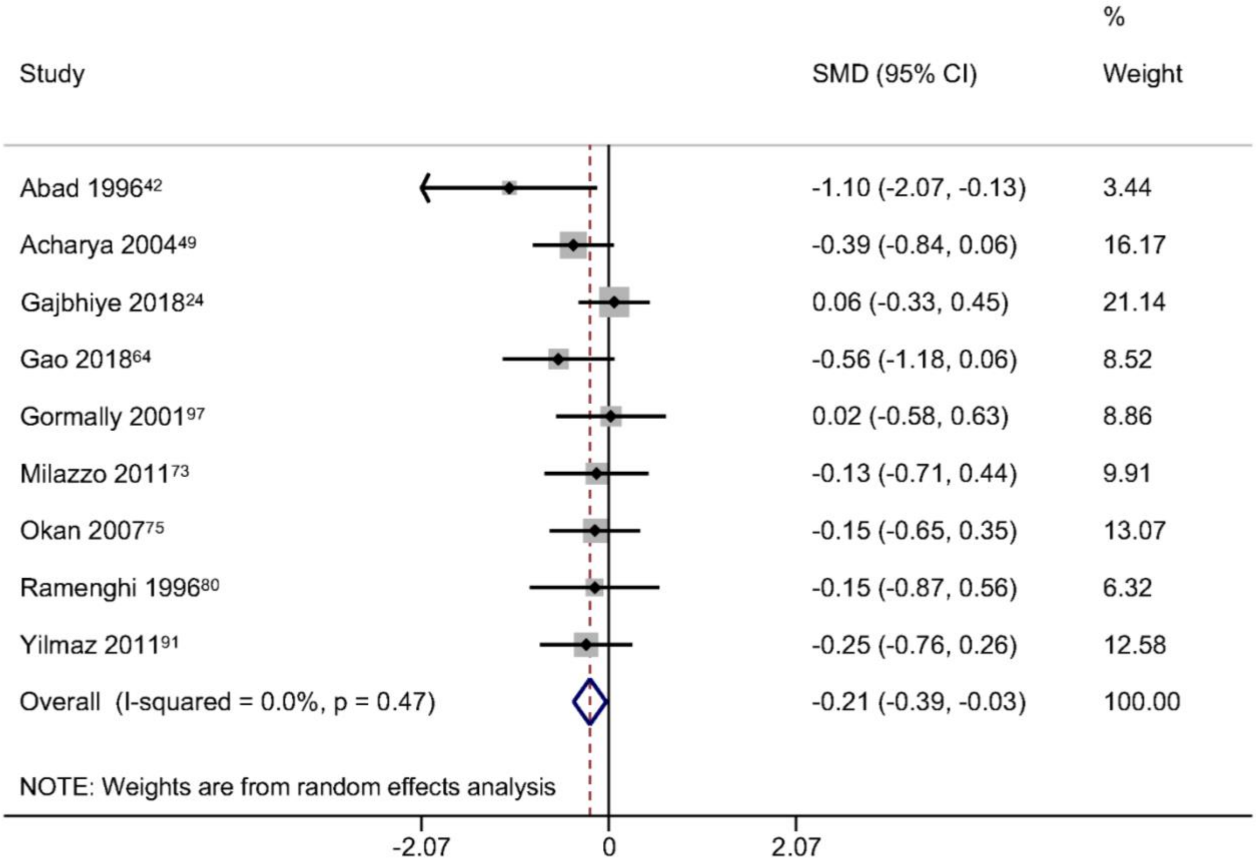

Note: P-value for test of standardised mean difference: 0.023
Abbreviations: CI=confidence interval, SMD=standardised mean difference

Supplementary Figure 33. Standardised mean differences and their confidence intervals for the comparison of oral dextrose versus placebo or no intervention for oxygen saturation

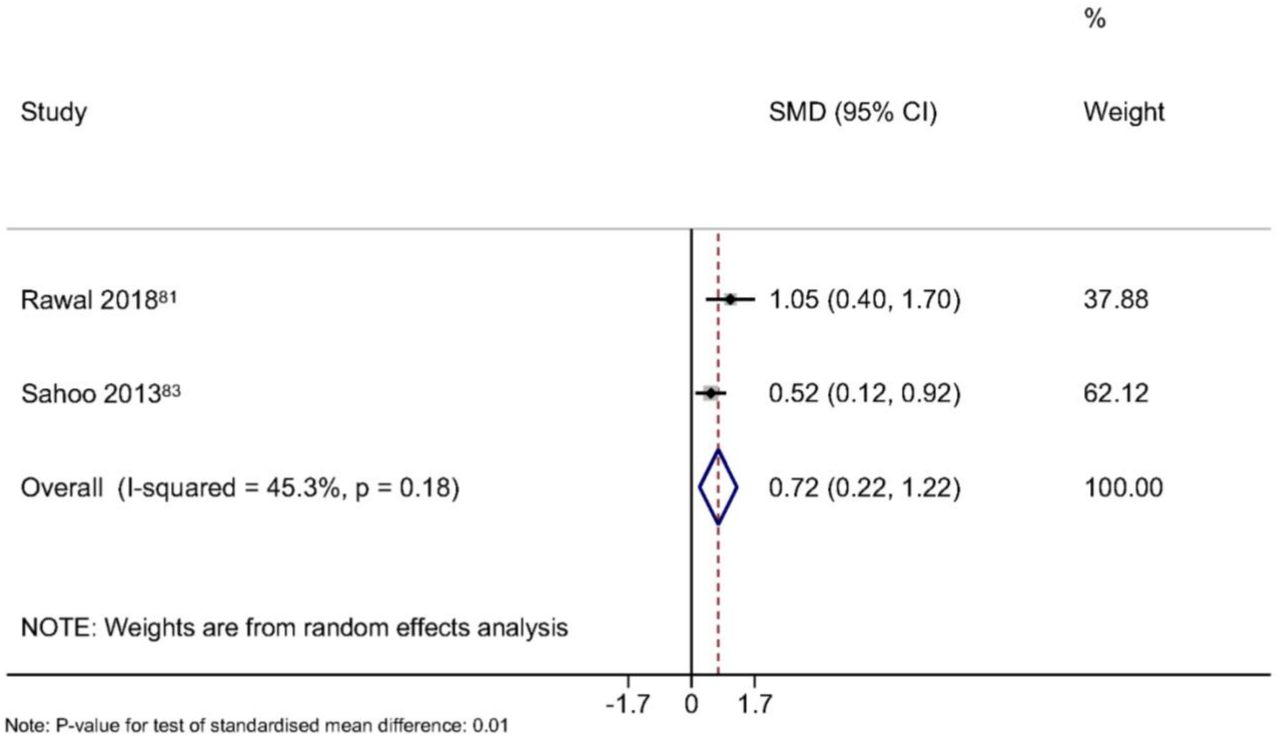

Note: P-value for test of standardised mean difference: 0.01

Abbreviations: CI=confidence interval, SMD=standardised mean difference

Supplementary Figure 34. Standardised mean differences and their confidence intervals for the comparison of oral glucose versus placebo or no intervention for oxygen saturation

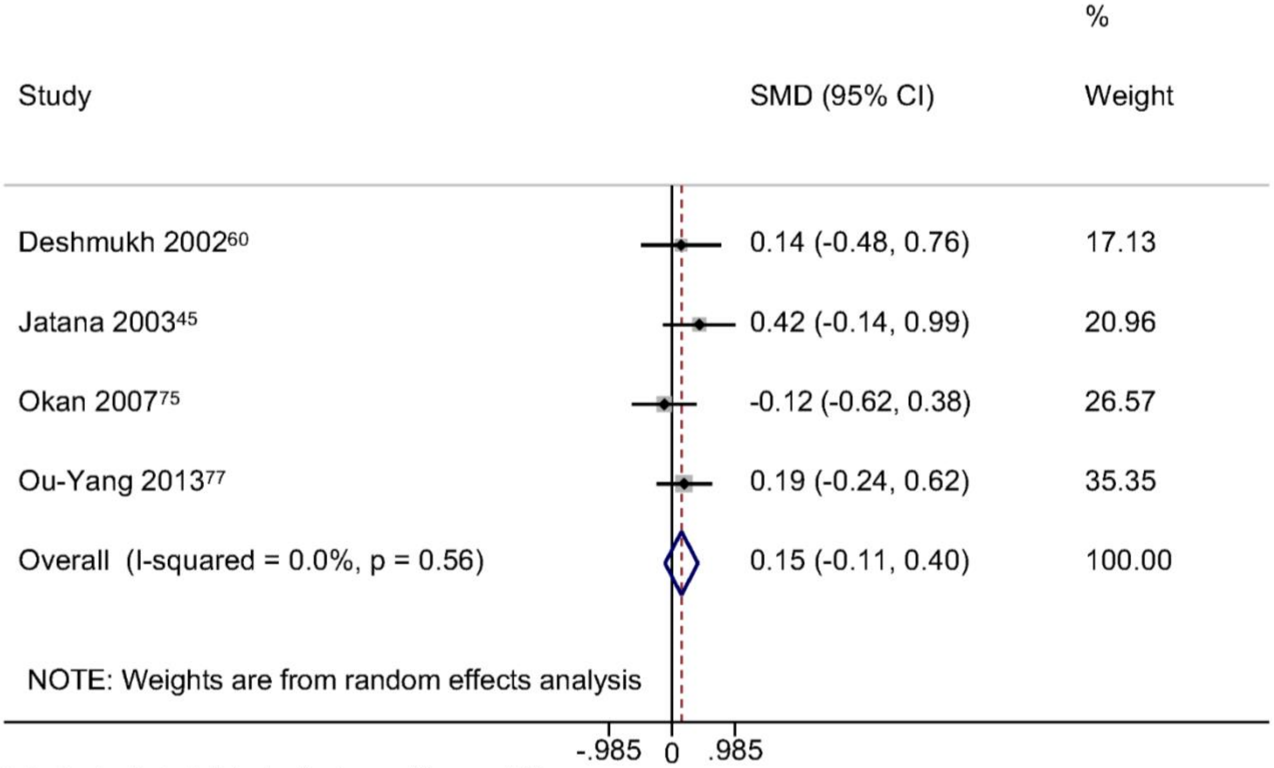

Note: P-value for test of standardised mean difference: 0.26

Abbreviations: CI=confidence interval, SMD=standardised mean difference

Supplementary Figure 35. Standardised mean differences and their confidence intervals for the comparison of oral sucrose versus placebo or no intervention for oxygen saturation

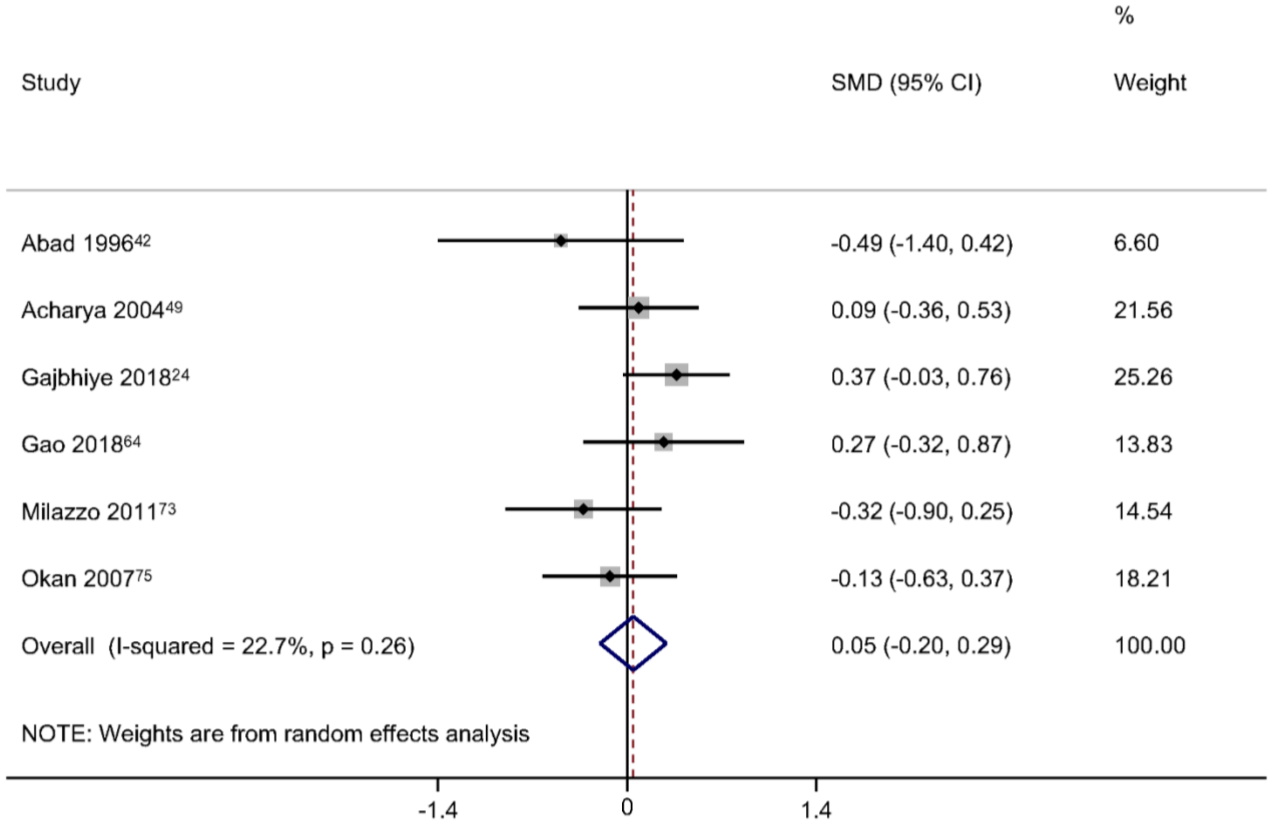

Note: P-value for test of standardised mean difference: 0.71

Abbreviations: CI=confidence interval, SMD=standardised mean difference

Supplementary Figure 36. Standardised mean differences and their confidence intervals for the comparison of oral dextrose versus placebo or no intervention for PIPP

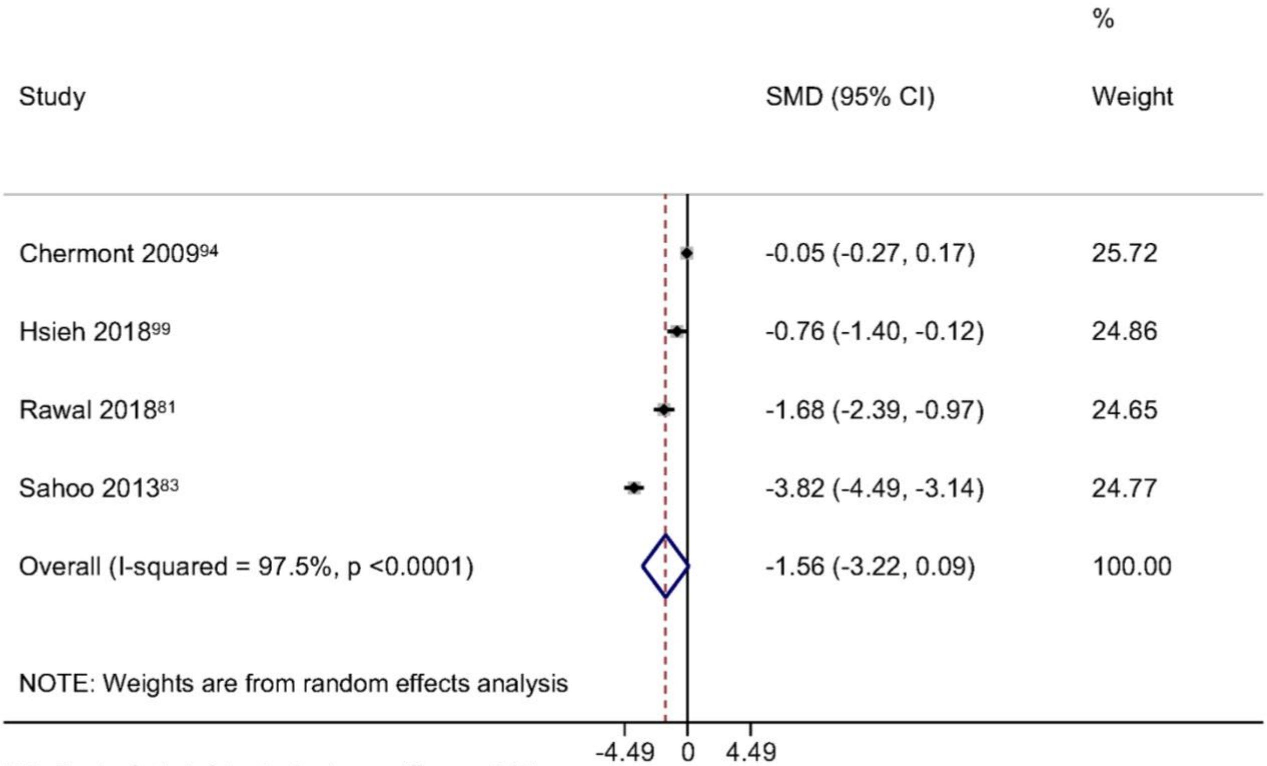

Note: P-value for test of standardised mean difference: 0.07

Abbreviations: CI=confidence interval, SMD=standardised mean difference, PIPP=Premature Infant Pain Profile

Supplementary Figure 37. Standardised mean differences and their confidence intervals for the comparison of oral glucose versus placebo or no intervention for PIPP

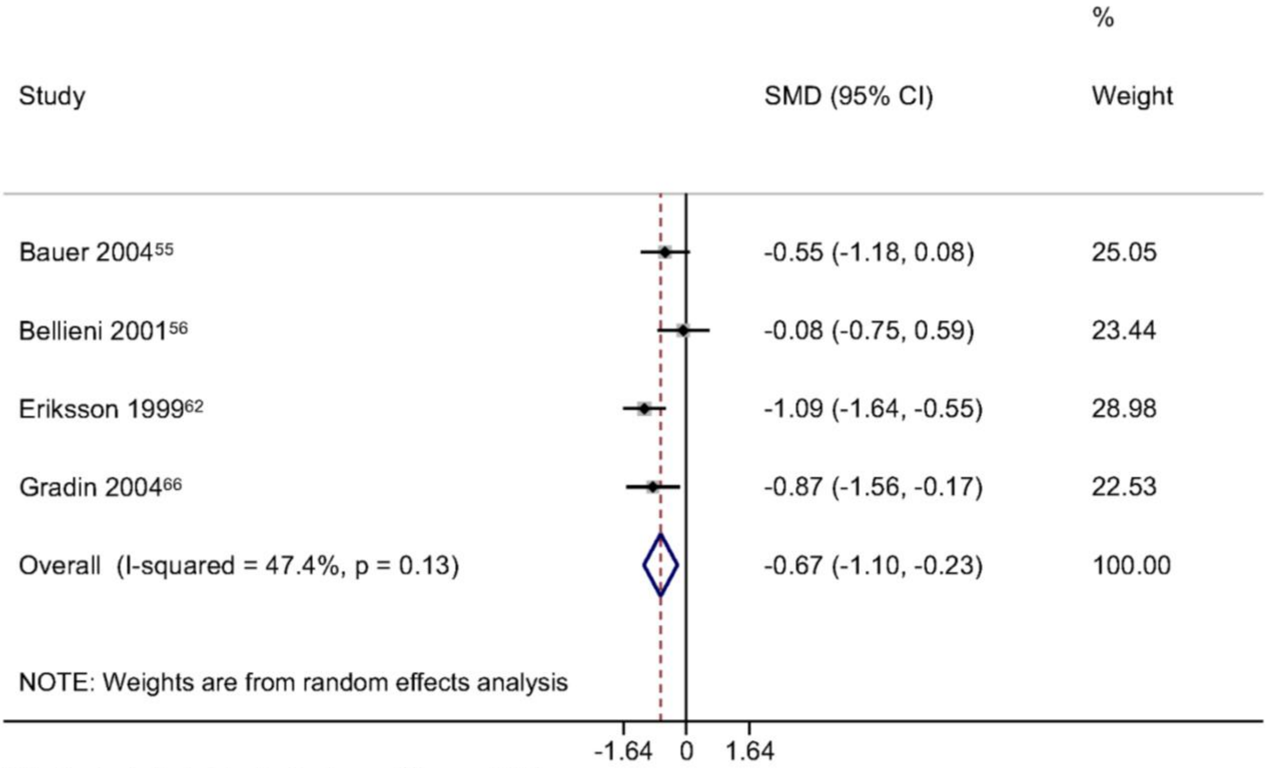

Note: P-value for test of standardised mean difference: 0.003

Abbreviations: CI=confidence interval, SMD=standardised mean difference, PIPP=Premature Infant Pain Profile

Supplementary Figure 38. Standardised mean differences and their confidence intervals for the comparison of oral sucrose versus placebo or no intervention for PIPP

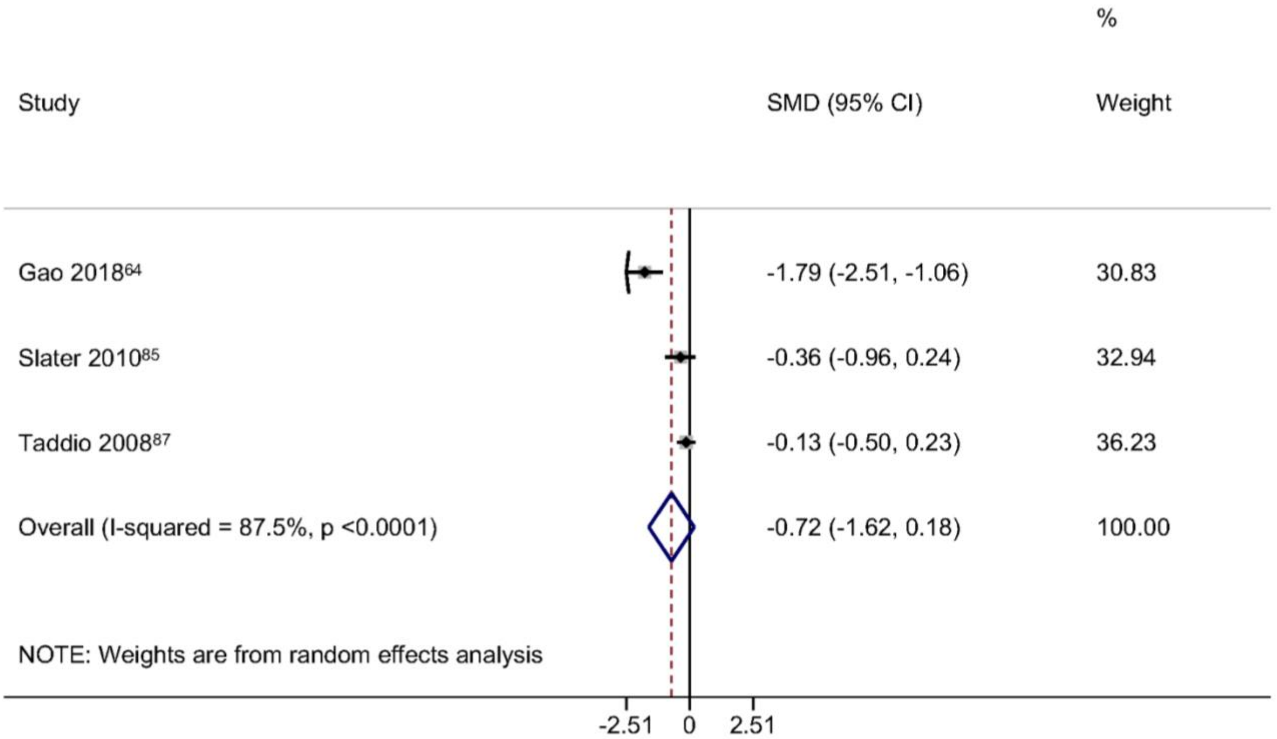

Note: P-value for test of standardised mean difference: 0.12

Abbreviations: CI=confidence interval, SMD=standardised mean difference, PIPP=Premature Infant Pain Profile

Supplementary Figure 39. Standardised mean differences and their confidence intervals for the comparison of oral sugar versus no intervention only for heart rate

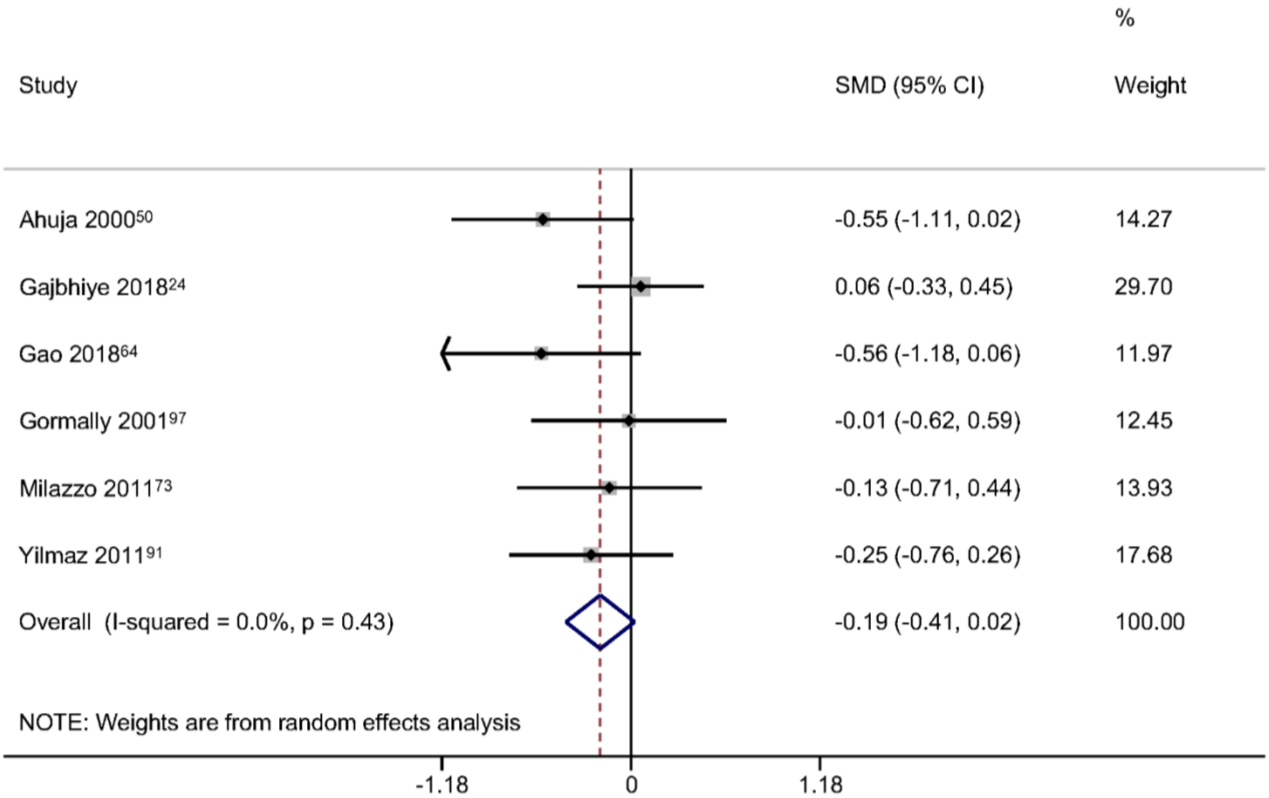

Note: P-value for test of standardised mean difference: 0.078

Abbreviations: CI=confidence interval, SMD=standardised mean difference

Supplementary Figure 40. Standardised mean differences and their confidence intervals for the comparison of oral sugar versus placebo only for heart rate

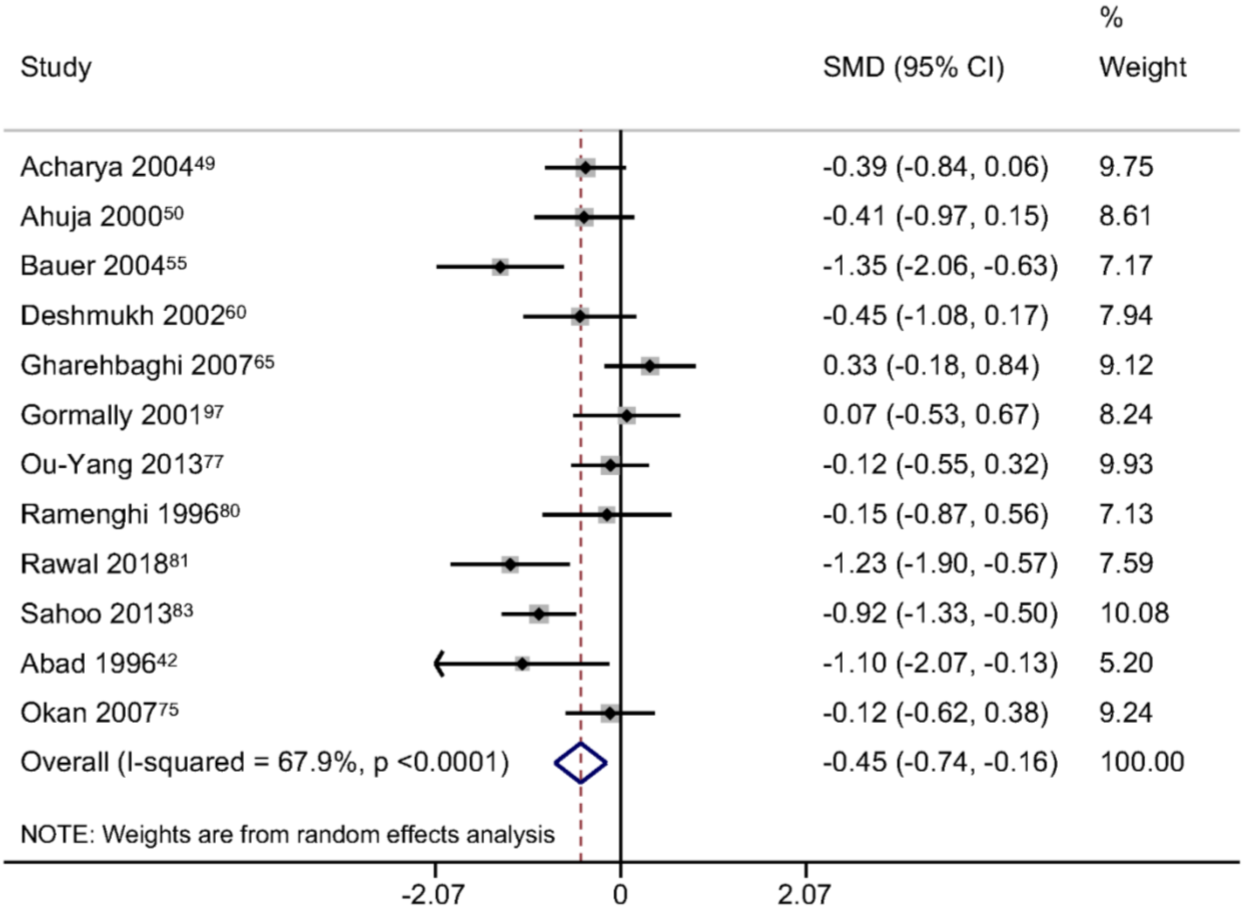

Note: P-value for test of standardised mean difference: 0.002
Abbreviations: CI=confidence interval, SMD=standardised mean difference

Supplementary Figure 41. Standardised mean differences and their confidence intervals for the comparison of oral sugar versus no intervention only for oxygen saturation

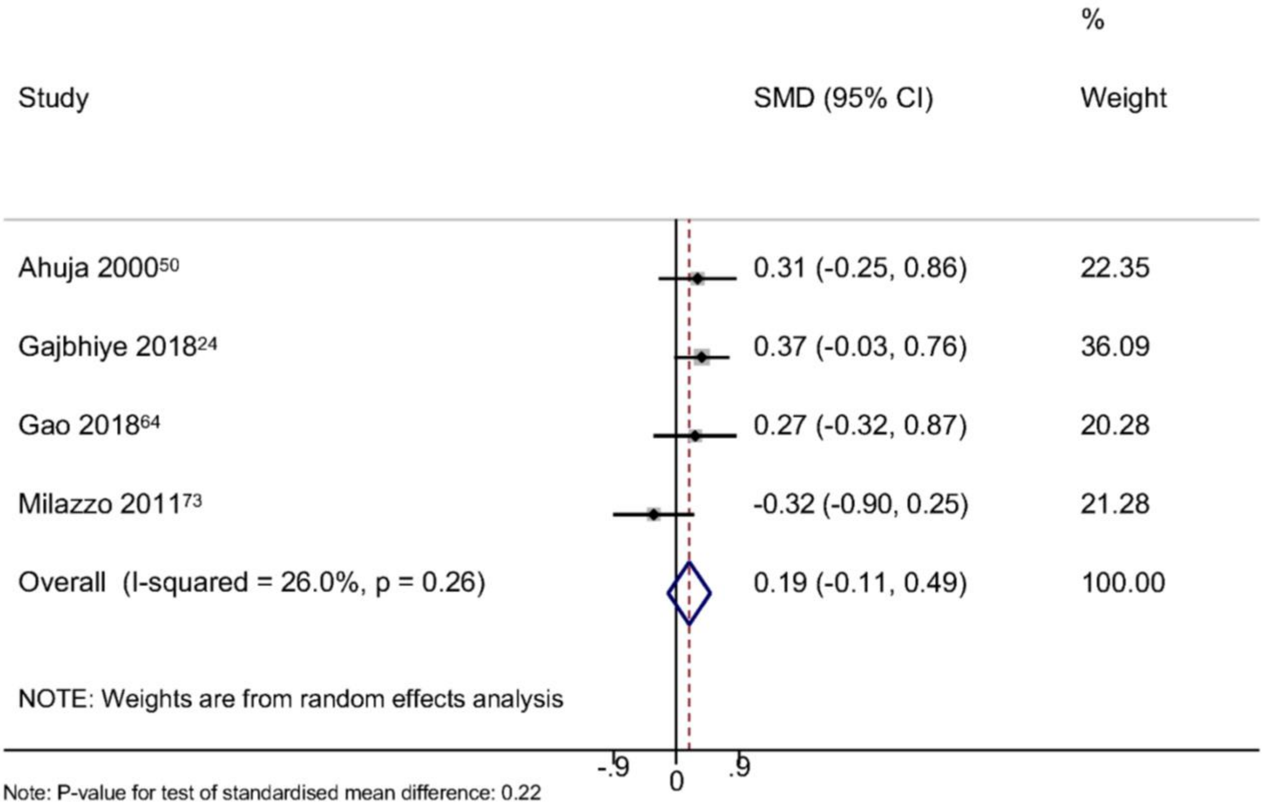

Note: P-value for test of standardised mean difference: 0.22

Abbreviations: CI=confidence interval, SMD=standardised mean difference

Supplementary Figure 42. Standardised mean differences and their confidence intervals for the comparison of oral sugar versus placebo only for oxygen saturation

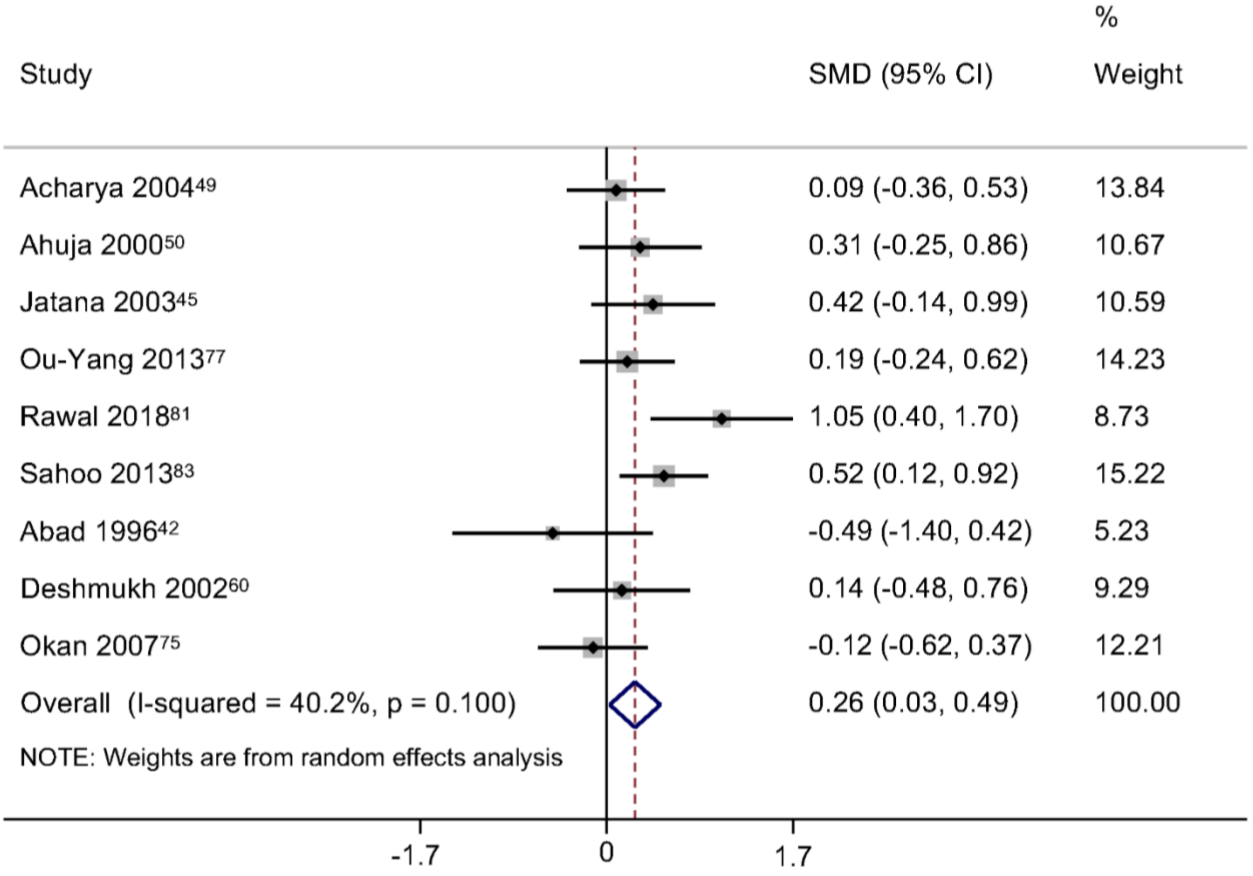

Note: P-value for test of standardised mean difference: 0.027

Abbreviations: CI=confidence interval, SMD=standardised mean difference

Supplementary Figure 43. Standardised mean differences and their confidence intervals for the comparison of oral sugar versus no intervention only for PIPP

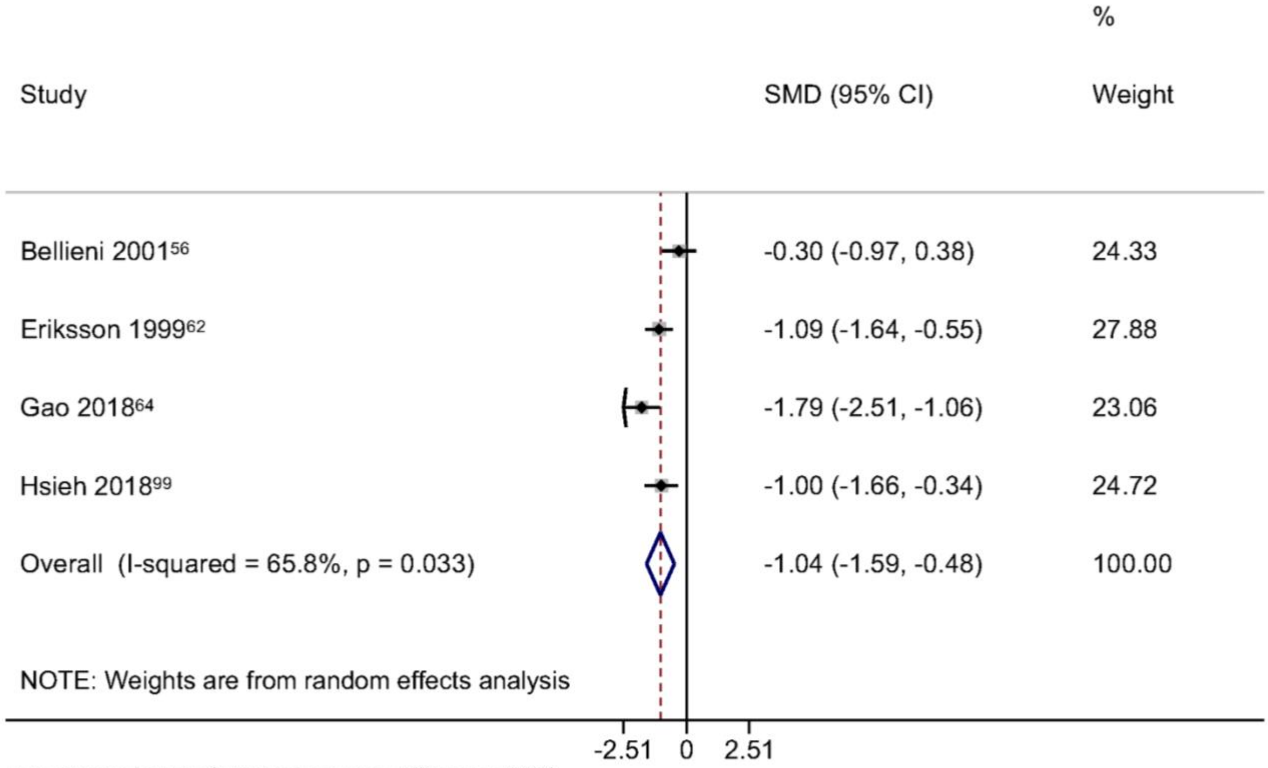

Note: P-value for test of standardised mean difference: <0.001

Abbreviations: CI=confidence interval, SMD=standardised mean difference, PIPP=Premature Infant Pain Profile

Supplementary Figure 44. Standardised mean differences and their confidence intervals for the comparison of oral sugar versus placebo only for PIPP

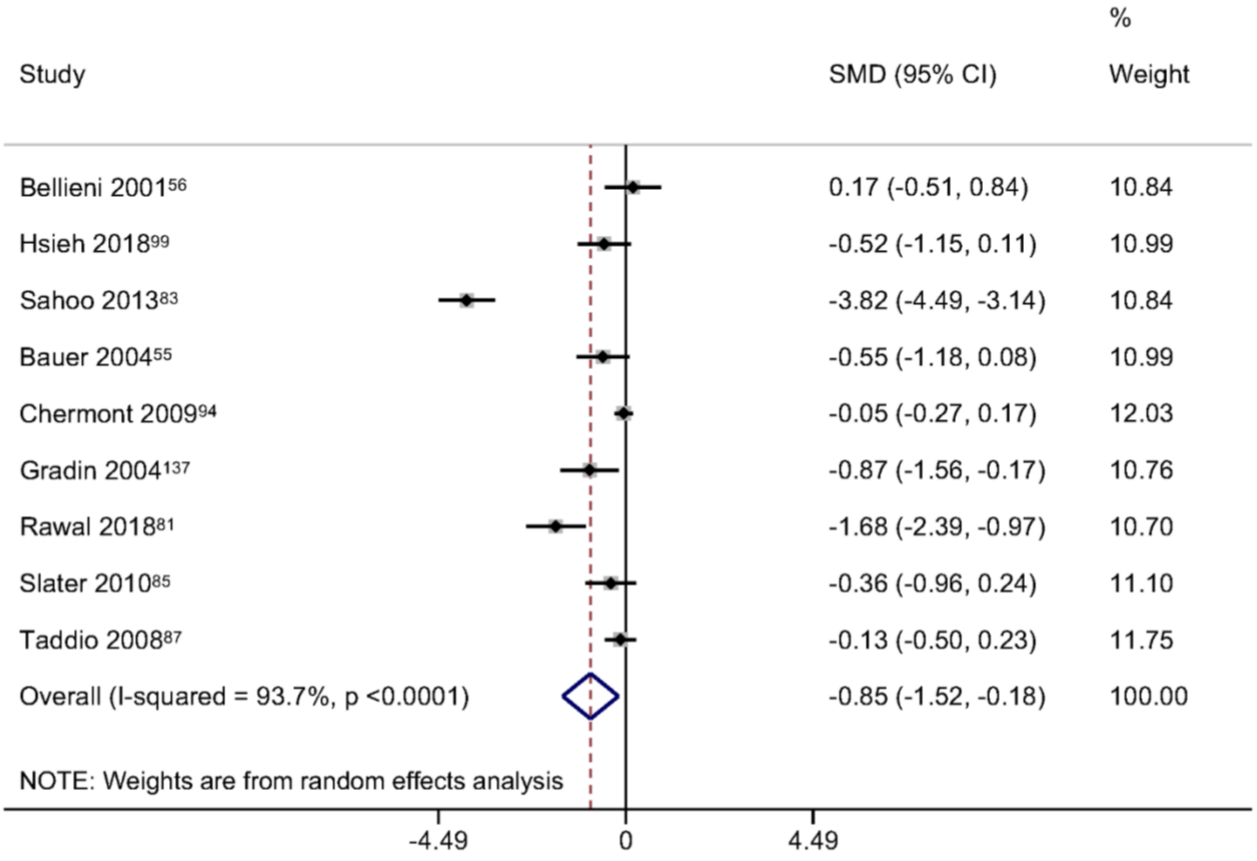

Note: P-value for test of standardised mean difference: 0.001

Abbreviations: CI=confidence interval, SMD=standardised mean difference, PIPP=Premature Infant Pain Profile

Supplementary Figure 45. Standardised mean differences and their confidence intervals for the comparison of oral sugar versus placebo or no intervention with heart rate measured ≤1 minute after procedure commencement

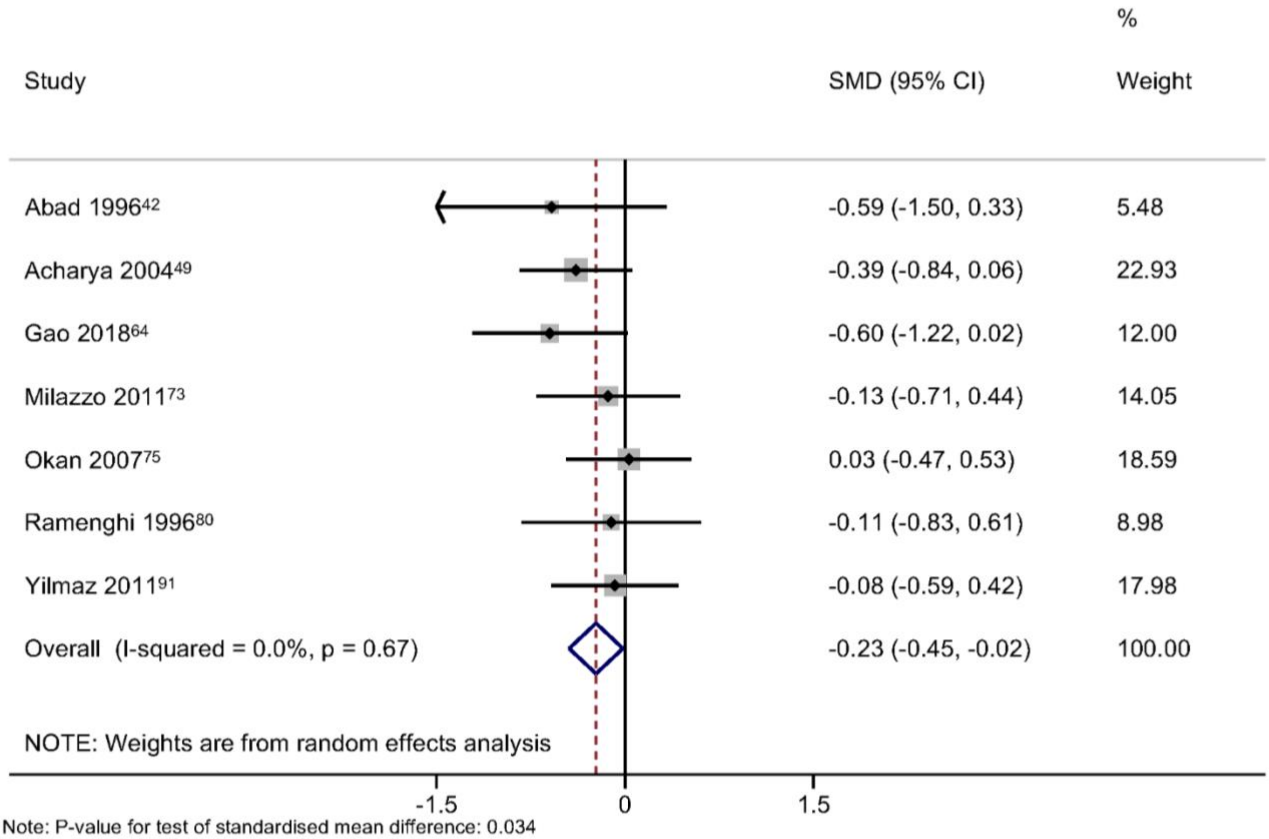

Note: P-value for test of standardised mean difference: 0.034

Abbreviations: CI=confidence interval, SMD=standardised mean difference

Supplementary Figure 46. Standardised mean differences and their confidence intervals for the comparison of oral sugar versus placebo or no intervention with heart rate measured >1 minute after procedure commencement

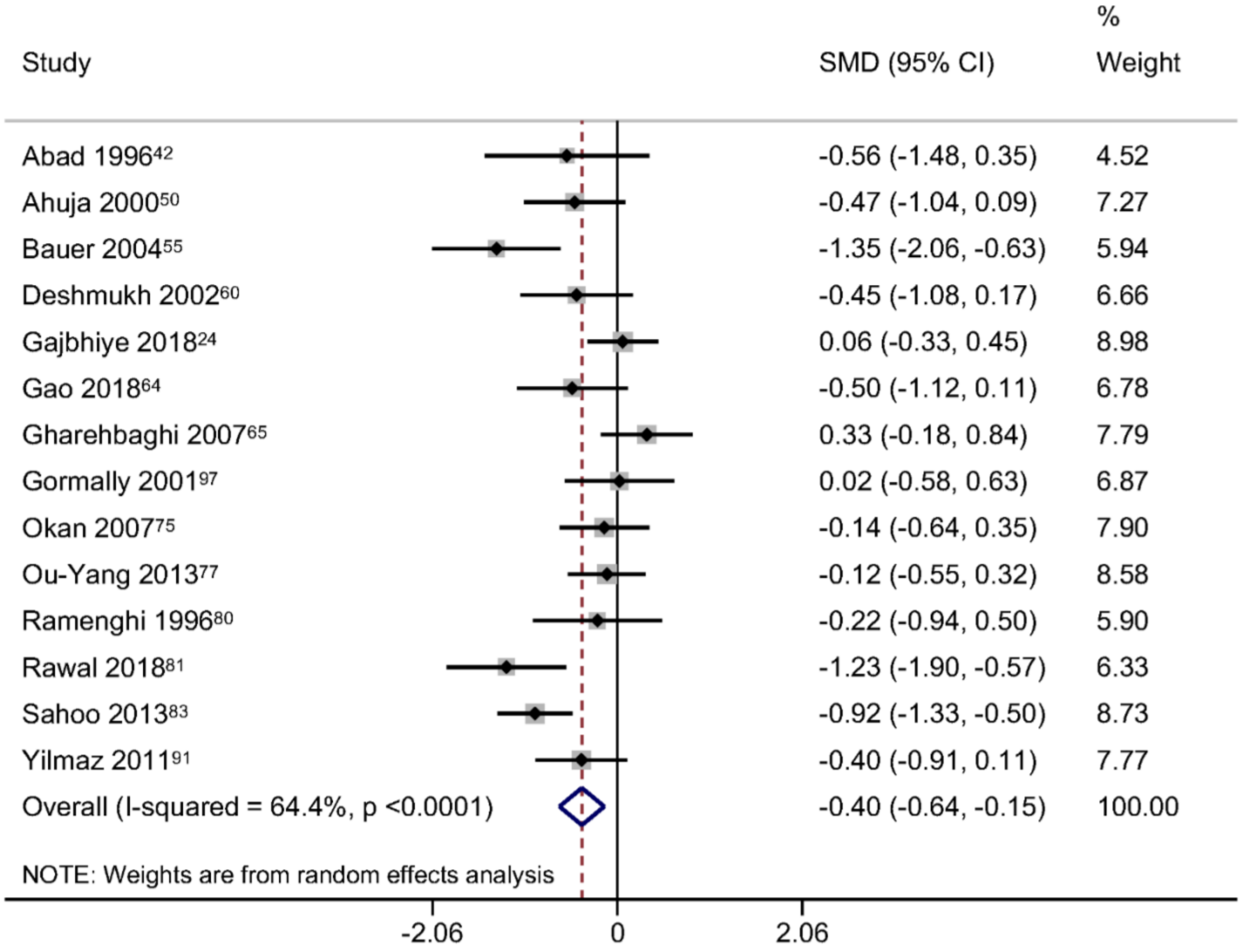

Note: P-value for test of standardised mean difference: 0.002
Abbreviations: CI=confidence interval, SMD=standardised mean difference

Supplementary Figure 47. Standardised mean differences and their confidence intervals for the comparison of oral sugar versus placebo or no intervention with oxygen saturation measured ≤1 minute after procedure commencement

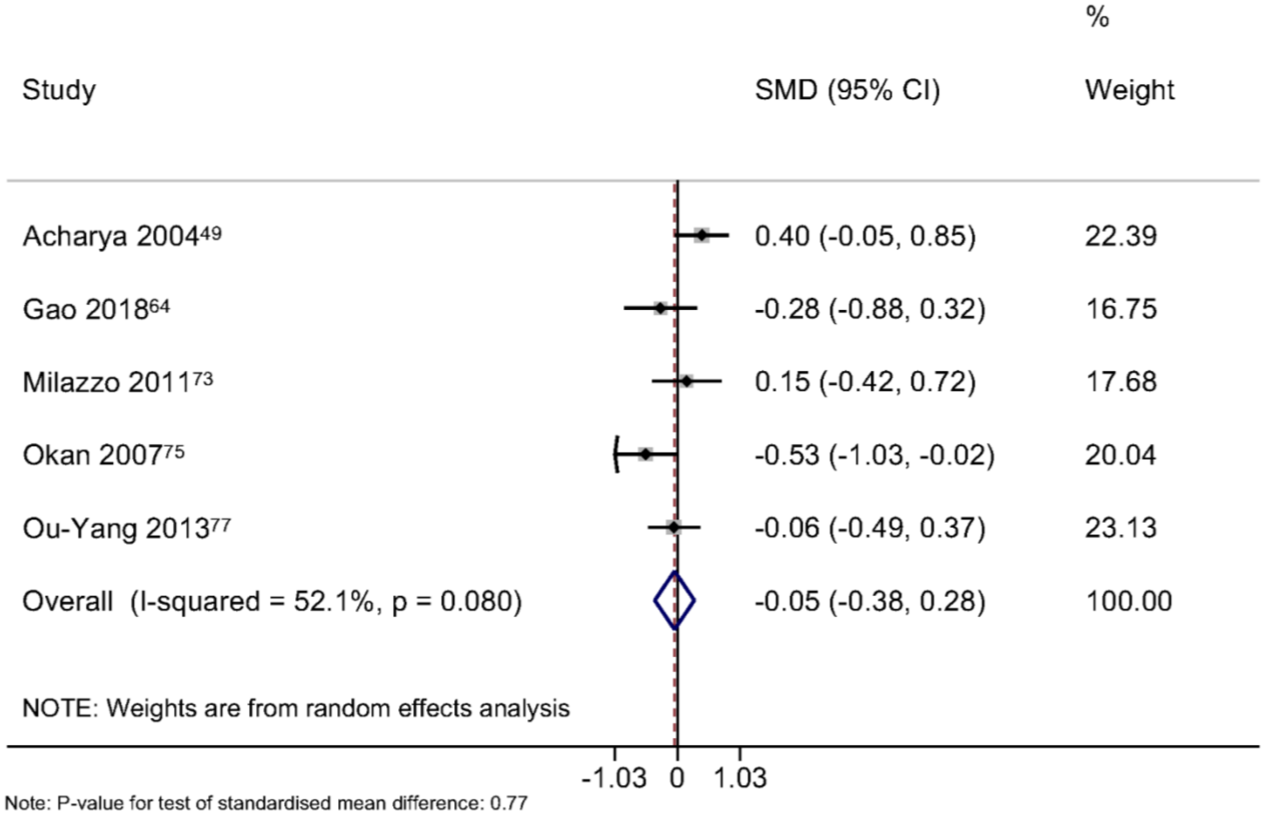

Note: P-value for test of standardised mean difference: 0.77

Abbreviations: CI=confidence interval, SMD=standardised mean difference

Supplementary Figure 48. Standardised mean differences and their confidence intervals for the comparison of oral sugar versus placebo or no intervention with oxygen saturation measured >1 minute after procedure commencement

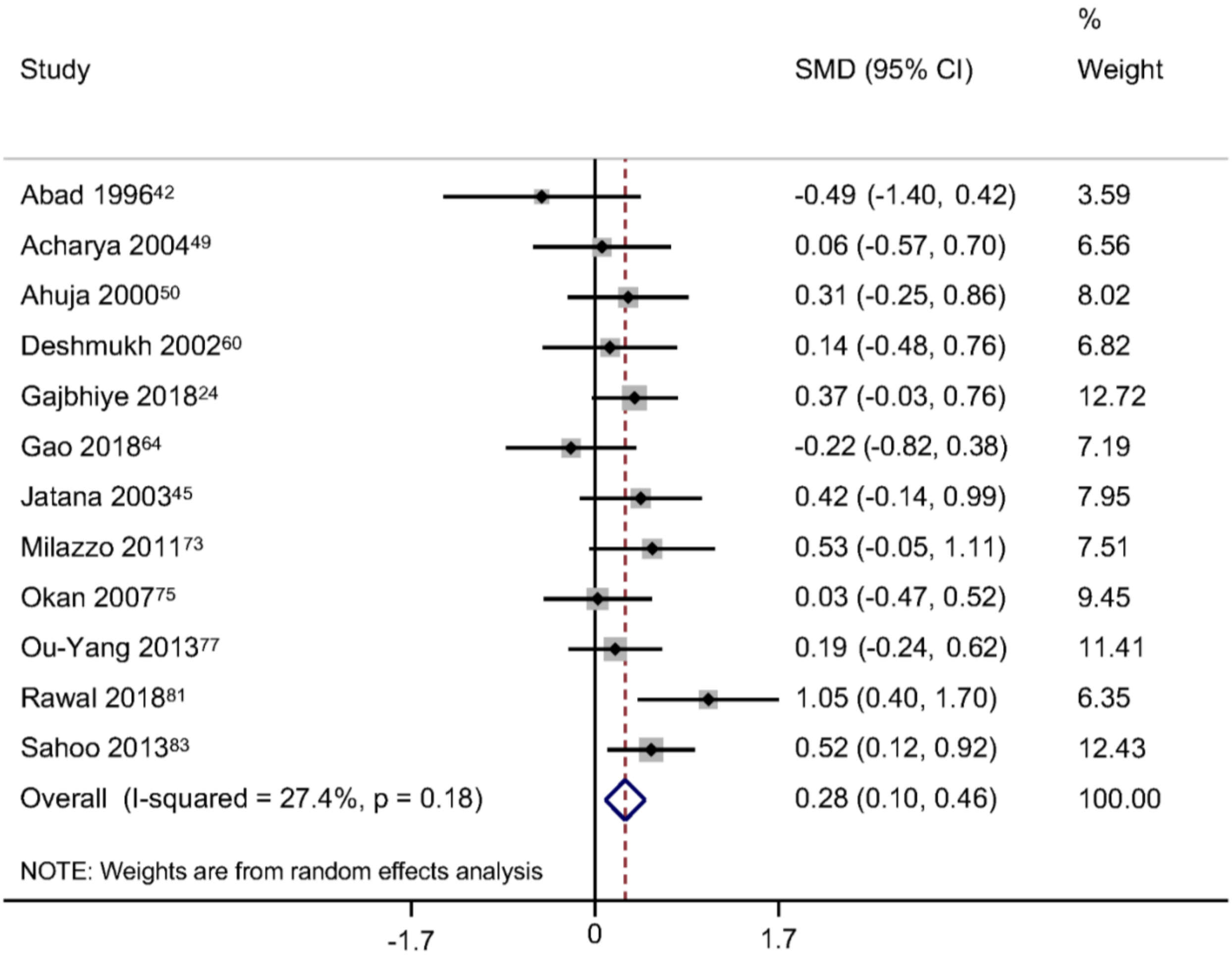

Note: P-value for test of standardised mean difference: 0.003

Abbreviations: CI=confidence interval, SMD=standardised mean difference

Supplementary Figure 49. Standardised mean differences and their confidence intervals for the comparison of oral sugar versus placebo or no intervention with PIPP measured ≤1 minute after procedure commencement

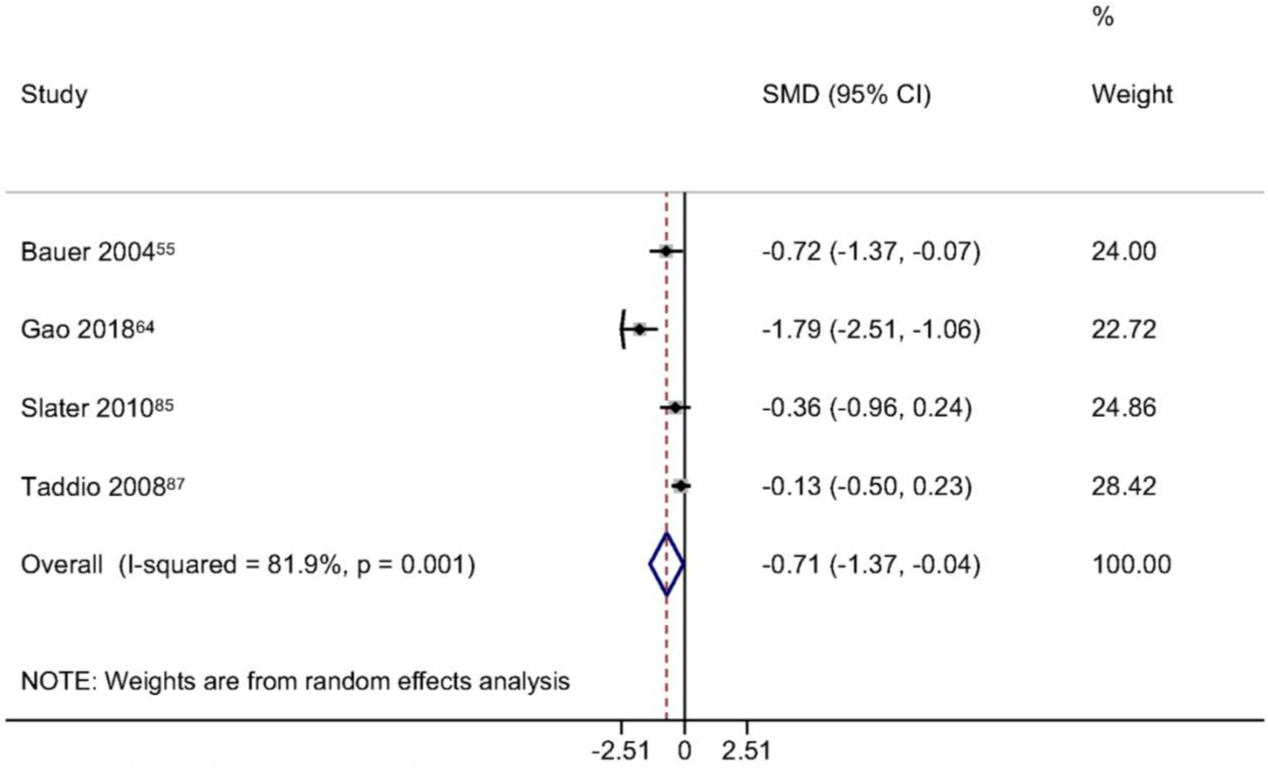

Note: P-value for test of standardised mean difference: 0.038

Abbreviations: CI=confidence interval, SMD=standardised mean difference, PIPP=Premature Infant Pain Profile

Supplementary Figure 50. Standardised mean differences and their confidence intervals for the comparison of oral sugar versus placebo or no intervention with PIPP measured >1 minute after procedure commencement

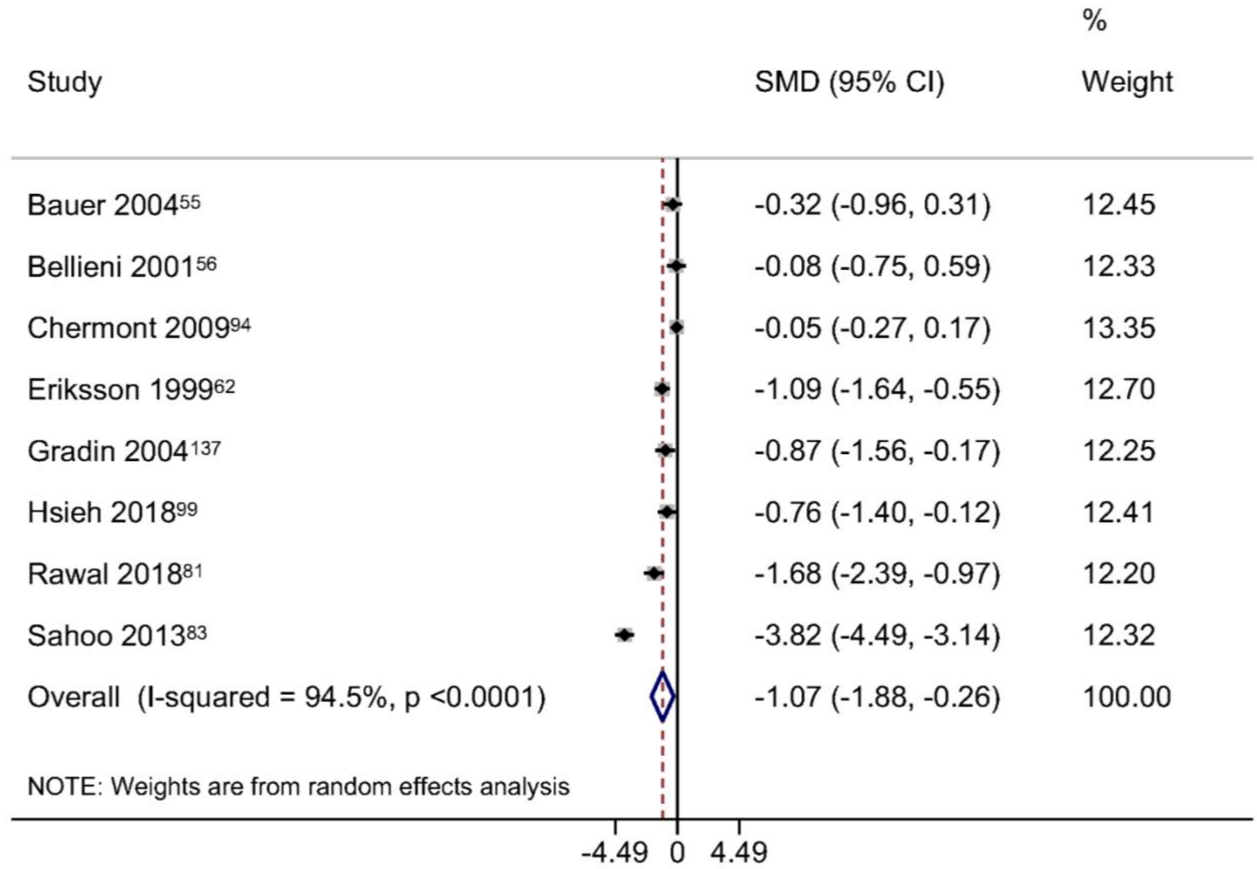

Note: P-value for test of standardised mean difference: 0.010

Abbreviations: CI=confidence interval, SMD=standardised mean difference, PIPP=Premature Infant Pain Profile

Supplementary Figure 51. Standardised mean differences and their confidence intervals for the comparison of oral sugar versus placebo or no intervention for heart rate by prematurity status

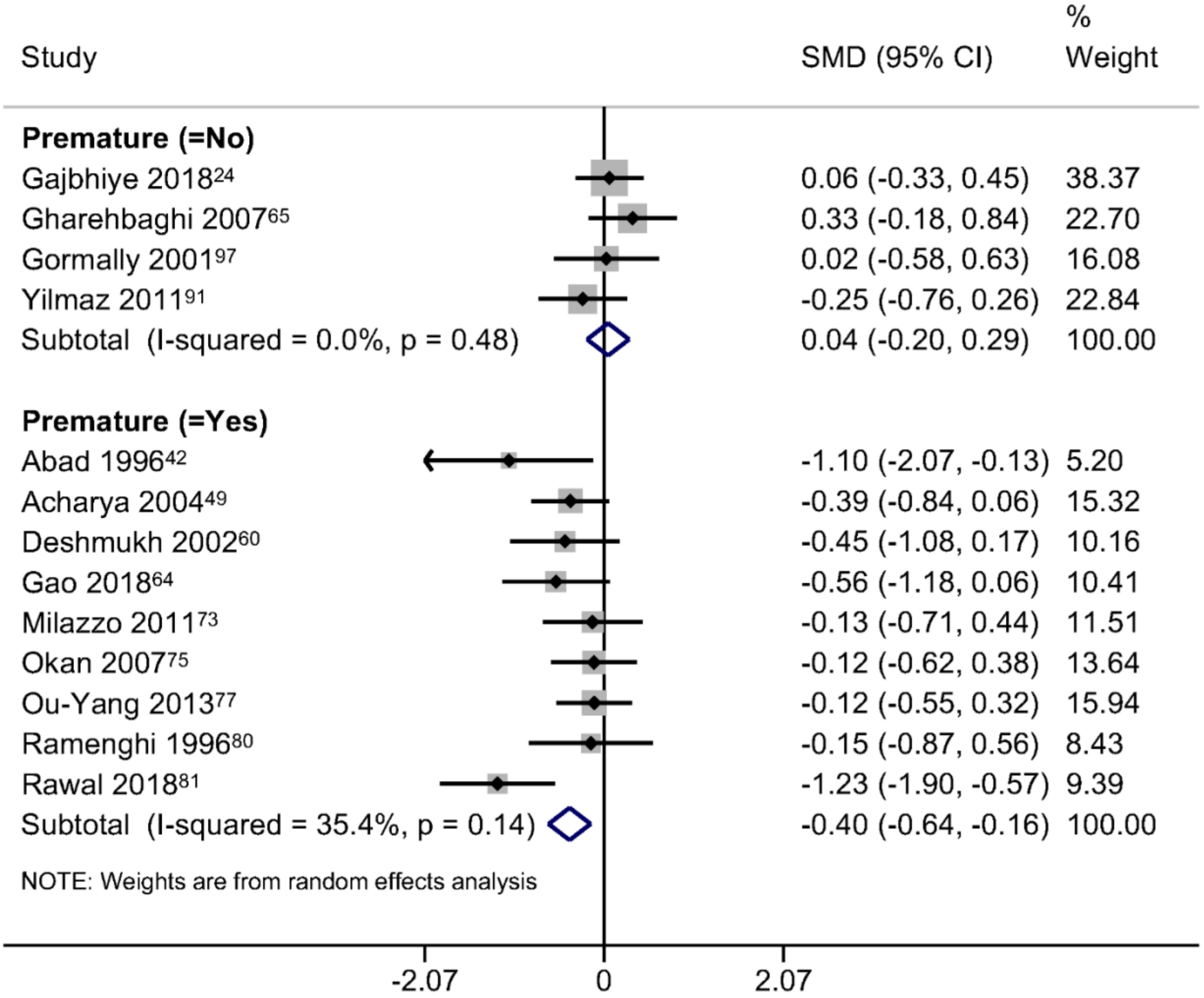

Note: P-value for interaction: 0.002

Abbreviations: CI=confidence interval, SMD=standardised mean difference

Supplementary Figure 52. Standardised mean differences and their confidence intervals for the comparison of oral sugar versus placebo or no intervention for oxygen saturation by prematurity status

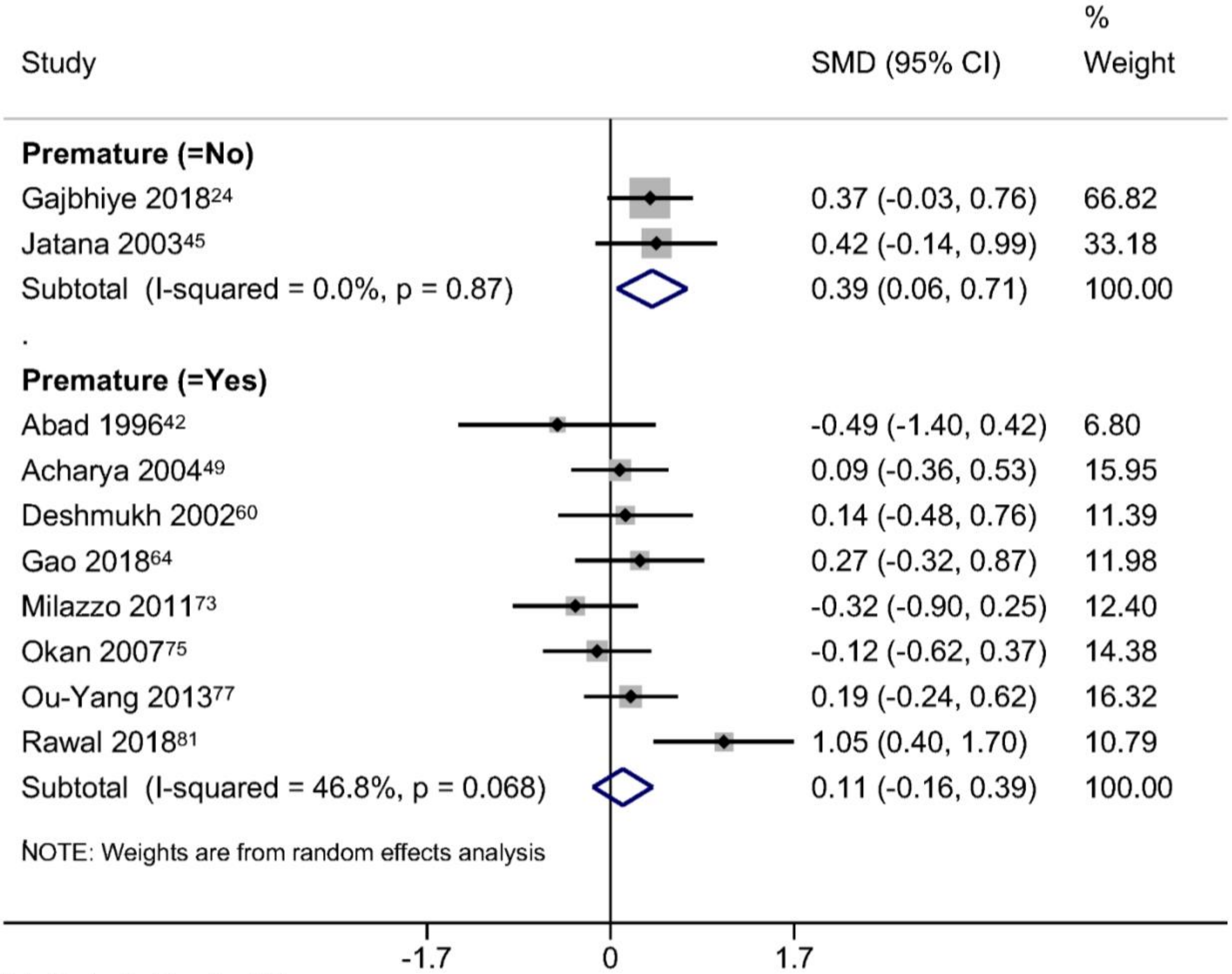

Note: P-value for interaction: 0.65

Abbreviations: CI=confidence interval, SMD=standardised mean difference

Supplementary Figure 53. Standardised mean differences and their confidence intervals for the comparison of oral sugar versus placebo or no intervention for PIPP by prematurity status

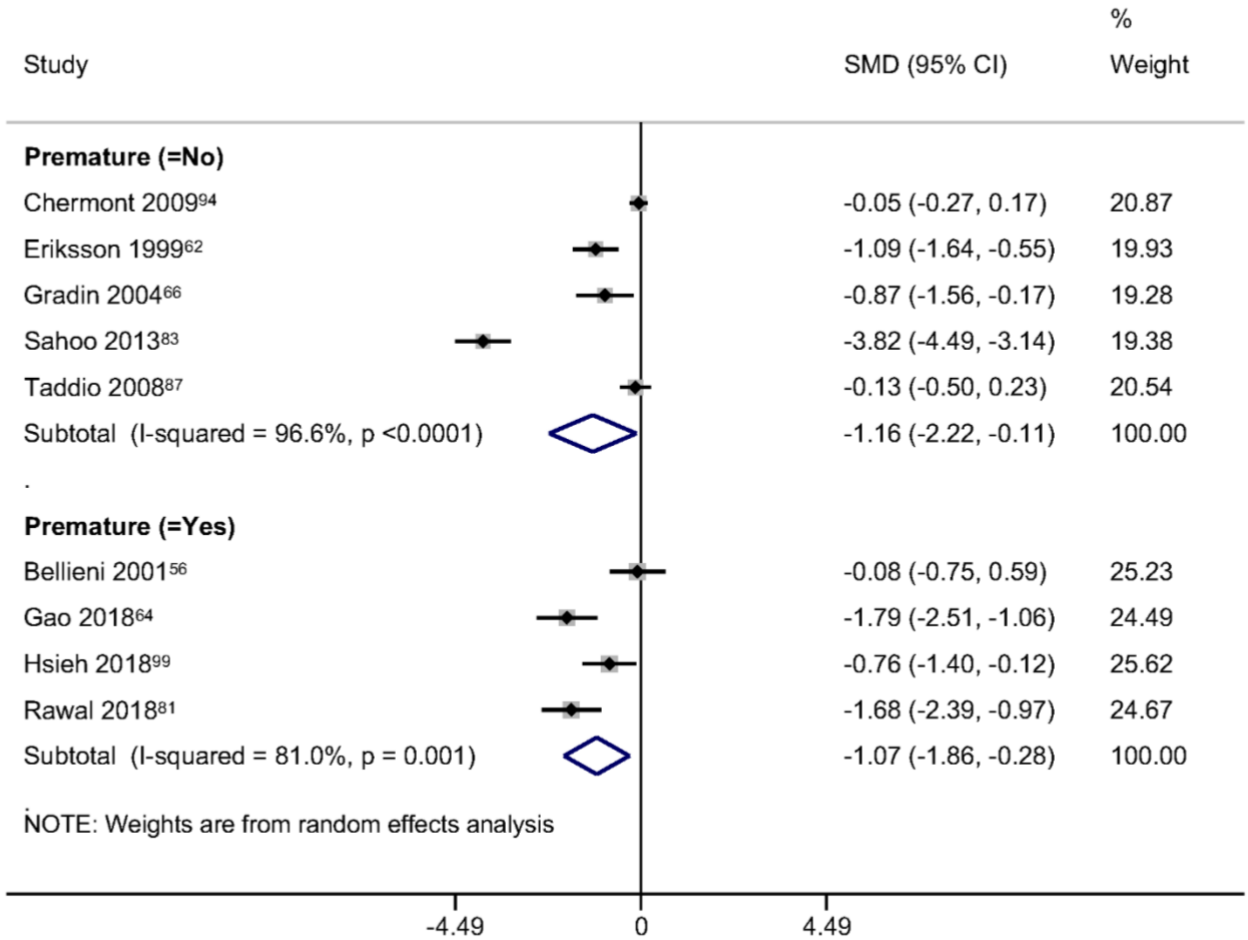

Note: P-value for interaction: 0.48

Abbreviations: CI=confidence interval, SMD=standardised mean difference, PIPP=Premature Infant Pain Profile

Supplementary Figure 54. Funnel plot (with pseudo 95% confidence limits) of studies for oral sugar versus placebo or no intervention using heart rate[24, 42, 49, 50, 55, 60, 64, 65, 73, 75, 77, 80, 81, 83, 91, 97]

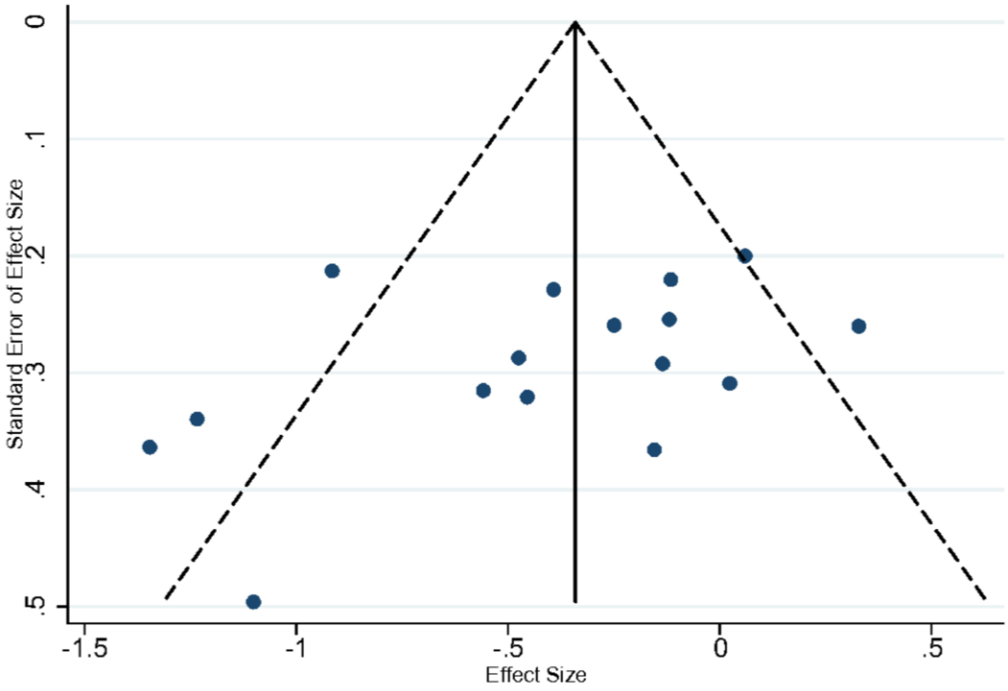

Supplementary Figure 55. Funnel plot (with pseudo 95% confidence limits) of studies for oral sugar versus placebo or no intervention using oxygen saturation[24, 42, 49, 50, 60, 64, 45, 73, 75, 77, 81, 83]

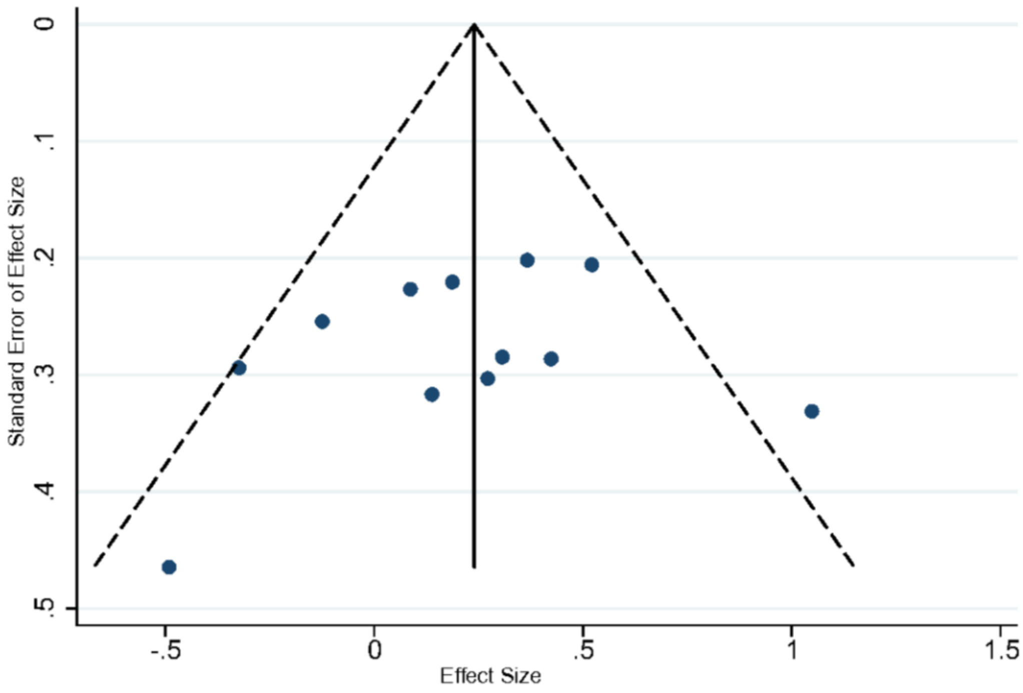

Supplementary Figure 56. Funnel plot (with pseudo 95% confidence limits) of studies for oral sugar versus placebo or no intervention using Premature Infant Pain Profile (PIPP)[55, 56, 62, 64, 66, 81, 83, 85, 87, 94, 99]

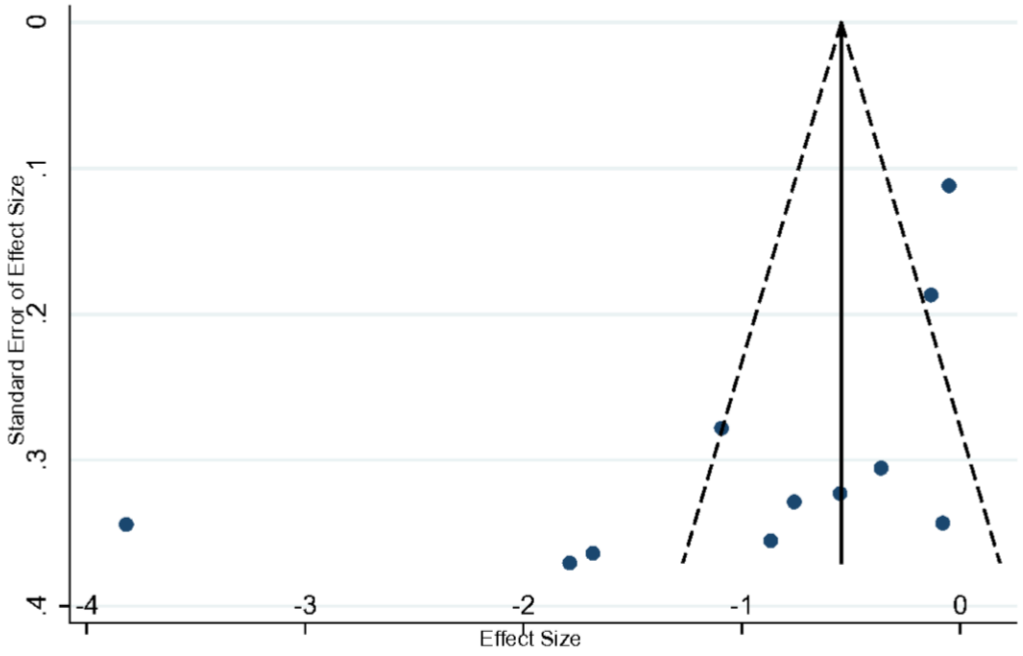

Further reading panel of references for PICO questions deprioritised by the Neonatal Pain Guideline Group

130 Acharya AB, Bustani PC, Phillips JD, Taub NA, Beattie RM. Randomised controlled trial of eutectic mixture of local anaesthetics cream for venepuncture in healthy preterm infants. Arch Dis Child Fetal Neonatal Ed 1998; 78: F138–42.
131 Badiee Z, Torcan N. Effects of high dose orally administered paracetamol for heel prick pain in premature infants. Saudi Med J 2009; 30: 1450–53.
132 Beken S, Hirfanoğlu IM, Gücüyener K, et al. Cerebral hemodynamic changes and pain perception during venipuncture: is glucose really effective? J Child Neurol 2014; 29: 617–22.
133 Bellieni CV, Aloisi AM, Ceccarelli D, et al. Intramuscular injections in newborns: analgesic treatment and sex-linked response. J Matern Fetal Neonatal Med 2013; 26: 419–22.
134 Biran V, Gourrier E, Cimerman P, Walter-Nicolet E, Mitanchez D, Carbajal R. Analgesic effects of EMLA cream and oral sucrose during venipuncture in preterm infants. Pediatrics 2011; 128: e63–70.
135 Bucher HU, Moser T, von Siebenthal K, Keel M, Wolf M, Duc G. Sucrose reduces pain reaction to heel lancing in preterm infants: a placebo-controlled, randomized and masked study. Pediatr Res 1995; 38: 332–35.
136 Butt ML, Kisilevsky BS. Music modulates behaviour of premature infants following heel lance. Can J Nurs Res 2000; 31: 17–39.
137 Campos RG. Soothing pain-elicited distress in infants with swaddling and pacifiers. Child Dev 1989; 60: 781–92.
138 Collados-G.mez L, Ferrera-Camacho P, Fernandez-Serrano E, et al. Randomised crossover trial showed that using breast milk or sucrose provided the same analgesic effect in preterm infants of at least 28 weeks. Acta Paediatr 2018; 107: 436–41.
139 De Bernardo G, Riccitelli M, Sordino D, et al. Oral 24% sucrose associated with nonnutritive sucking for pain control in healthy term newborns receiving venipuncture beyond the first week of life. J Pain Res 2019; 12: 299–305.
140 De Melo G, Cardoso M. Pain in preterm newborns submitted to music and 25% glucose intervention. Revista da Rede de Enfermagem do Nordeste 2017; 18: 3.
141 Erkut Z, Yildiz S. The effect of swaddling on pain, vital signs, and crying duration during heel lance in newborns. Pain Manag Nurs 2017; 18: 328–36.
142 Gerull R, Cignacco E, Stoffel L, Sellam G, Nelle M. Physiological parameters after nonpharmacological analgesia in preterm infants: a randomized trial. Acta Paediatr 2013; 102: e368–73.
143 Gibbins S, Stevens B, Hodnett E, Pinelli J, Ohlsson A, Darlington G. Efficacy and safety of sucrose for procedural pain relief in preterm and term neonates. Nurs Res 2002; 51: 375–82.
144 Gradin M, Eriksson M, Holmqvist G, Holstein A, Schollin J. Pain reduction at venipuncture in newborns: oral glucose compared with local anesthetic cream. Pediatrics 2002; 110: 1053–57.
145 Ho LP, Ho SS, Leung DY, So WK, Chan CW. A feasibility and efficacy randomised controlled trial of swaddling for controlling procedural pain in preterm infants. J Clin Nurs 2016; 25: 472–82.
146 Holsti L, Oberlander TF, Brant R. Does breastfeeding reduce acute procedural pain in preterm infants in the neonatal intensive care unit? A randomized clinical trial. Pain 2011; 152: 2575–81.
147 Huang CM, Tung WS, Kuo LL, Ying-Ju C. Comparison of pain responses of premature infants to the heelstick between containment and swaddling. J Nurs Res 2004; 12: 31–40.
148 Jain A, Rutter N. Does topical amethocaine gel reduce the pain of venepuncture in newborn infants? A randomised double blind controlled trial. Arch Dis Child Fetal Neonatal Ed 2000; 83: F207–10.
149 Johnston CC, Filion F, Campbell-Yeo M, et al. Kangaroo mother care diminishes pain from heel lance in very preterm neonates: a crossover trial. BMC Pediatr 2008; 8: 13.
150 Kaur G, Gupta P, Kumar A. A randomized trial of eutectic mixture of local anesthetics during lumbar puncture in newborns. Arch Pediatr Adolesc Med 2003; 157: 1065–70.
151 Kristoffersen L, Malahleha M, Duze Z, et al. Randomised controlled trial showed that neonates received better pain relief from a higher dose of sucrose during venepuncture. Acta Paediatr 2018; 107: 2071–78.
152 Kurdahi Badr L, Demerjian T, Daaboul T, Abbas H, Hasan Zeineddine M, Charafeddine L. Preterm infants exhibited less pain during a heel stick when they were played the same music their mothers listened to during pregnancy. Acta Paediatr 2017; 106: 438–45.
153 Larsson BA, Tannfeldt G, Lagercrantz H, Olsson GL. Alleviation of the pain of venepuncture in neonates. Acta Paediatr 1998; 87: 774–79.
154 Lemyre B, Hogan DL, Gaboury I, Sherlock R, Blanchard C, Moher D. How effective is tetracaine 4% gel, before a venipuncture, in reducing procedural pain in infants: a randomized double-blind placebo controlled trial. BMC Pediatr 2007; 7: 7.
155 Leng HY, Zheng XL, Zhang XH, et al. Combined non-pharmacological interventions for newborn pain relief in two degrees of pain procedures: A randomized clinical trial. Eur J Pain 2016; 20: 989–97.
156 Lindh V, Wiklund U, H.kansson S. Assessment of the effect of EMLA during venipuncture in the newborn by analysis of heart rate variability. Pain 2000; 86: 247–54.
157 Long CP, McCafferty DF, Sittlington NM, Halliday HL, Woolfson AD, Jones DS. Randomized trial of novel tetracaine patch to provide local anaesthesia in neonates undergoing venepuncture. Br J Anaesth 2003; 91: 514–18.
158 Marcatto JO, Vasconcelos PC, Ara.jo CM, Tavares EC, Pereira e Silva Y. EMLA versus glucose for PICC insertion: a randomised triple-masked controlled study. Arch Dis Child Fetal Neonatal Ed 2011; 96: F467–68.
159 Mar.n Gabriel M., del Rey Hurtado de Mendoza B, Jim.nez Figueroa L, et al. Analgesia with breastfeeding in addition to skin-to-skin contact during heel prick. Arch Dis Child Fetal Neonatal Ed 2013; 98: F499–503.
160 Marofi M, Nikobakht F, Badiee Z, Golchin M. The effect of melody on the physiological responses of heel sticks pain in neonates. Iran J Nurs Midwifery Res 2015; 20: 405–08.
161 Morrow C, Hidinger A, Wilkinson-Faulk D. Reducing neonatal pain during routine heel lance procedures. MCN Am J Matern Child Nurs 2010; 35: 346–54, quiz 354–56.
162 Mosayebi Z, Javidpour M, Rahmati M, Hagani H, Movahedian A. The effect of kangaroo mother care on pain from heel lance in preterm newborns admitted to neonatal intensive care unit: a crossover randomized clinical trial. J Compr Pediatr 2014; 5: e22214.
163 Noori Shadkam M, Lofti M. Pain reducing in icteric newborns while venipuncturing: comparison of local anesthetic cream with orally glucose. Acta Med Iran 2008; 46: 58–62.
164 Patel A, Czerniawski B, Gray S, Lui E. Does topical amethocaine gel reduce pain from heel prick blood sampling in premature infants? A randomized double-blind cross-over controlled study. Paediatr Child Health 2003; 8: 222–25.
165 Peng HF, Yin T, Yang L, et al. Non-nutritive sucking, oral breast milk, and facilitated tucking relieve preterm infant pain during heel-stick procedures: a prospective, randomized controlled trial. Int J Nurs Stud 2018; 77: 162–70.
166 Rossi A, Molinaro A, Savi E, et al. Music reduces pain perception in healthy newborns: a comparison between different music tracks and recoded heartbeat. Early Hum Dev 2018; 124: 7–10.

167 Shabani F, Nayeri ND, Karimi R, Zarei K, Chehrazi M. Effects of music therapy on pain responses induced by blood sampling in premature infants: a randomized cross-over trial. Iran J Nurs Midwifery Res 2016; 21: 391–96.
168 Shah SR, Kadage S, Sinn J. Trial of music, sucrose, and combination therapy for pain relief during heel prick procedures in neonates. J Pediatr 2017; 190: 153–58.e2.
169 Shu SH, Lee YL, Hayter M, Wang RH. Efficacy of swaddling and heel warming on pain response to heel stick in neonates: a randomised control trial. J Clin Nurs 2014; 23: 3107–14.
170 Shukla V, Chapla A, Uperiya J, Nimbalkar A, Phatak A, Nimbalkar S. Sucrose vs skin to skin care for preterm neonatal pain control-a randomized control trial. J Perinatol 2018; 38: 1365–69.
171 Shukla VV, Bansal S, Nimbalkar A, et al. Pain control interventions in preterm neonates: a randomized controlled trial. Indian Pediatr 2018; 55: 292–96.
172 Soliman H, Elsharkawy A, Abdel-Hady H. Does topical lidocaine reduce the pain associated with the insertion of nasal continuous positive airway pressure prongs in preterm infants?: a randomized, controlled pilot trial. Clin J Pain 2016; 32: 948–54.
173 Stevens B, Johnston C, Franck L, Petryshen P, Jack A, Foster G. The efficacy of developmentally sensitive interventions and sucrose for relieving procedural pain in very low birth weight neonates. Nurs Res 1999; 48: 35–43.
174 Stevens B, Johnston C, Taddio A, et al. Management of pain from heel lance with lidocaine-prilocaine (EMLA) cream: is it safe and efficacious in preterm infants? J Dev Behav Pediatr 1999; 20: 216–21.
175 Stevens B, Yamada J, Campbell-Yeo M, et al. The minimally effective dose of sucrose for procedural pain relief in neonates: a randomized controlled trial. BMC Pediatr 2018; 18: 85.
176 Sujatha S, Samson R, Amalraj C. Sundaresan. Sucrose and facilitated tucking for pain among neonates receiving vaccination, in Puducherry. Int J Pharm Clinl Res 2017; 9: 260–63.
177 Taddio A, Shah V, Stephens D, et al. Effect of liposomal lidocaine and sucrose alone and in combination for venipuncture pain in newborns. Pediatrics 2011; 127: e940–47.
